# Pet snakes illegally marketed in Brazil: Climatic viability and establishment risk

**DOI:** 10.1371/journal.pone.0183143

**Published:** 2017-08-17

**Authors:** Érica Fonseca, Mirco Solé, Dennis Rödder, Paulo de Marco

**Affiliations:** 1 Departamento de Biologia, Programa de Pós-Graduação em Biodiversidade Animal, Universidade Federal de Santa Maria, Santa Maria, Rio Grande do Sul, Brazil; 2 Departamento de Ciências Biológicas, Programa de Pós-Graduação em Zoologia, Universidade Estadual de Santa Cruz, Ilhéus, Bahia, Brazil; 3 Department of Herpetology, Zoological Research Museum Alexander Koenig, Bonn, Germany; 4 Departamento de Ecologia, Instituto de Ciências Biológicas, Universidade Federal de Goiás Campus II, Goiânia, Goiás, Brazil; Universitat Trier, GERMANY

## Abstract

Invasive species are one among many threats to biodiversity. Brazil has been spared, generically, of several destructive invasive species. Reports of invasive snakes’ populations are nonexistent, but the illegal pet trade might change this scenario. Despite the Brazilian laws forbid to import most animals, illegal trade is frequently observed and propagules are found in the wild. The high species richness within Brazilian biomes and accelerated fragmentation of natural reserves are a critical factors facilitating successful invasion. An efficient way to ease damages caused by invasive species is identifying potential invaders and consequent prevention of introductions. For the identification of potential invaders many factors need to be considered, including estimates of climate matching between areas (native vs. invaded). Ecological niche modelling has been widely used to predict potential areas for invasion and is an important tool for conservation biology. This study evaluates the potential geographical distribution and establishment risk of *Lampropeltis getula* (Linnaeus, 1766), *Lampropeltis triangulum* (Lacépède, 1789), *Pantherophis guttatus* (Linnaeus, 1766), *Python bivittatus* Kuhl, 1820 and *Python regius* (Shaw, 1802) through the Maximum Entropy modelling approach to estimate the potential distribution of the species within Brazil and qualitative evaluation of specific biological attributes. Our results suggest that the North and Midwest regions harbor major suitable areas. Furthermore, *P*. *bivittatus* and *P*. *guttatus* were suggested to have the highest invasive potential among the analyzed species. Potentially suitable areas for these species were predicted within areas which are highly relevant for Brazilian biodiversity, including several conservation units. Therefore, these areas require special attention and preventive measures should be adopted.

## Introduction

Biological invasions represent one of the principal threats to global biodiversity [1]. Either accidentally or intentionally, the introduction of exotic species [2] is responsible for the invasion of various species around the world, many of which have caused severe damage to the natural environment and wildlife [3–5]. Factors that determine which species will be successful invaders involve a number of complex interactions between the species and the biotic and abiotic components of the invaded region [6–8]. Thus, the identification of potential invaders needs additional evaluation, seeking to identify the main useful biotic and abiotic factors for the identification of target species. For reptiles and amphibians, there is a strong relationship between the success of establishment and specific attributes of the species [9–11], comprising the number of offspring, taxonomic group, history of establishment in other regions and climate matching between areas [11]. The latter, however, is recognized as the most important factor in cases of invasion [12].

Recent advances in modeling techniques and the development of free online databases of distribution have favored predictions about the potential distribution of species based on ecological niche modeling techniques (ENM) [13]. Several authors have used this concept for identification of potential distribution of invasive species [14–18]. The application of predictive models covers only part of the evaluation process of invasion risk [19], but provides valuable information which can be used to rank invasion potential for different species for a given area and to target control activities. Using this approach, it is possible to understand complex aspects of species invasion. For instance, considering species used as pets, a significant change occurs in the accessibility of novel habitats, since a species can virtually access any place in the world through pet trade. Thus, its establishment in new areas is now determined almost exclusively by biotic and abiotic factors without a clear limitation of access to new locations. On the other hand, and especially for exotic species, outside of their original habitat and on large scales, where climatic influences are dominant in species distribution, the impact of biotic interactions are minimized, so the interspecific interactions become less relevant [20–22].

Several species of reptiles have become pets in many parts of the world, with possible serious effects on the increase of invasive propagules outside of their native distributions [9,23–25]. For instance, there is an extensive list of snake species that have been introduced around the world, like *Python bivittatus*, *Boiga irregularis* (Bechstein 1802), *Acrochordus javanicus* Hornstedt, 1787, *Boa constrictor* Linnaeus 1758, *Lampropeltis getula*, *Pantherophis guttatus*, *Natrix maura* (Linnaeus, 1758), *Macroprotodon cucullatus* (Geoffroy-St-Hilaire, 1827), *Rhinechis scalaris* (Schinz, 1822), *Lycodon aulicus* (Linnaeus, 1758) and *Indotyphlops braminus* (Daudin, 1803) [26–40]. However, there is only little evidence related to the potential consequences of these introductions, wherein the largest body of evidence concern *Boiga irregularis* and *Python bivittatus* [41,42].

In Brazil, although there are records of other invasive herpetofauna elements [43–48], there are no reports of established invasive populations of snakes living in natural surroundings in the country. However, in the period between 1995 and 2000, 66 snakes of 16 exotic species were found only in the state of São Paulo [49]. These specimens are probably derived from the illegal trade of pets, which occurs freely in shopping portals and social networks on the Internet. From 2006 to 2012, it was possible to trace additional 44 reptile species being marketed in southeastern Brazil, in which more than half (26) were snakes [50]. Although the import of animals is prohibited by law (Ordinance 93/1998), some species have been entering the country and propagules have been found in natural areas [49–51] increasing establishment risk [52]. After successful establishment of those specimens, control and management costs become higher, and total eradication may be in most cases virtually impossible [53,54]. Therefore, the identification of potential invaders and the prevention of new introductions is the most economical and efficient way to deal with this problem [55].

In this paper we evaluated the potential geographical distribution and establishment risk of five species of exotic pet snakes in Brazil. For this, we identified areas of high bioclimatic suitability for establishment of populations of exotic snakes, calculated the probability of success of establishment of the species and identified the species with greatest establishment risk, through qualitative evaluation and comparison of specific biological attributes.

## Materials and methods

### Selection of species and occurrence data

The criteria catalogue which was used for the selection of target species was based on the list of exotic species sold illegally in southeastern Brazil, published by Magalhães and São-Pedro [50]. Only those species with the highest number of internet advertising available for sale were selected, considering that more traded species present a higher risk of escape or being released in the environment and consequently a higher propagules pressure of these species. Thus, the selected species comprised: *Lampropeltis getula*, *Lampropeltis triangulum Pantherophis guttatus*, *Python regius*, and *Python bivittatus*.

We compiled unique occurrence records of *L*. *getula* (1.156), *L*. *triangulum* (1.071), *P*. *guttatus* (385), *P*. *bivittatus* (82) and *P*. *regius* (29) from the literature and from the Global Biodiversity Information Facility–GBIF (Access on November 4, 2013) (S1 Datasets). Species records represent the native distribution of the selected species, with the exception of *P*. *guttatus*, for which records of established, non-native populations were also used.

Data obtained from biological collections may, in some cases, show signatures of geographic sampling bias limiting the predictive capacity of the model (see [56, 57]). To avoid potential effects of sampling bias, the species records were divided into 10 random subgroups each randomly split in training (70% of occurrence point data) and test points (30% of occurrence point data) that was used for validation. In each of the 10 groups, the points are at least 100 kilometers apart from each other to remove spatial autocorrelation. The entire procedure was performed at the Matrix Laboratory—MATLAB 7.6.0.

### Environmental variables

Since potential distributions are a projection of the climate conditions at the species records within the species’ native ranges, it is not possible to say that the variables that limit the species are necessarily the same in different regions. In this way, we used all 20 environmental variables, spatial resolution of 10 arc min (approximately 18 km^2^), available on Worldclim. The set of environmental data was subjected to Principal Components Analysis (PCA). PCA’s objective is to reduce a large number of measures in a few synthetic variables, enabling their use to describe the system, without much loss of information [58]. Thus, in order to capture major parts of the environmental variation with the fewest possible axes, we selected the axes representing at least 95% of the total variation for being used as new variables (S1 Tables). Projections in non-analogous climates might raise extrapolation and interpolation errors [59], so to provide an uncertainty measure for extrapolation we calculated the Multivariate Environmental Similarity Surfaces (MESS). MESS maps indicate areas which climate condition exceed those of the training areas and hence where results must be interpreted carefully due to extrapolation [60]. Data from this study have been deposited in figshare (DOI: 10.6084/m9.figshare.5268058).

### Analytical method, modeling procedures and evaluation

We used Maxent—Maximum Entropy [61]—to develop the models. Maxent estimates the potential geographical distribution of species from presence data and random background information based on the maximum entropy probability, i.e., seeking a distribution closer to uniformity (maximum state of disruption). Some studies have shown that Maxent produces better results than other comparable methods [62–64], and has an excellent performance in the prediction of appropriate areas for invasive species [12,16,65]. To avoid excessive parameterizing, all models were generated using the linear and quadratic features only, as discussed by Syfert et al. [66]. In the Maxent configurations, only random source options were selected, and duplicate species records were removed. In the advanced settings, 1000 interactions and otherwise default settings were used.

As suggested by Liu et al. [67], the evaluation of the performance of the models was based on True Skill Statistics (TSS) [68]. The TSS is a measure of precision that takes into account false negatives and false positives, assigning equal weight to sensitivity (real presences correctly predicted) and specificity (actual absences correctly predicted), providing a measure dependent on the precision threshold [68]. It indicates how the model is in agreement with reality, with values ranging from -1 to 1. Values close to 0 or less indicate a performance not much better than random. Values greater than 0.7 are considered to indicate excellent models. According to Allouche et al. [68], the TSS is insensitive to the prevalence (number of presence points by area), being a suitable alternative to replace AUC (area under the ROC curve).

To meet the goals of the study, and aiming at a practical application of the models, ordinal logistic Maxent scores were transformed into presence-absence predictions through the ROC threshold, “Maximum training sensitivity plus specificity”. The ROC threshold was chosen since it balances the errors of false negative and false positives, producing smaller, more reliable potential distribution. All calculations were performed on the R Development Core Team [69] using the PresenceAbsence package [70]. A total of 50 model were generated—10 for each species. These models were then evaluated and subsequently added (via GIS tools) so that one model was generated for each species. In every procedure, distributions were predicted for the region of origin of each species (Asia, Africa and North America) and later designed for the area of interest. Finally, after the insertion of the thresholds, the models were combined to generate a consensus model, which identifies how many models predict the presence of species in each location. In this model, it is possible to identify areas of greater fitness, where most (or all) models indicated that the establishment of those species was propitious.

### Evaluation of invasion risk

To calculate the probability of success of establishing and assessing the potential of invasive species, we used the method developed by van Wilgen and Richardson [71], here called SPHE Model (Success Probability of the Herpetofauna Establishment). These authors used data of introduced non-native reptiles and amphibians in California and Florida to identify which factors are most influential in the establishment success of the herpetofauna. According to the contribution of the variables, they developed a model to indicate the probability of herpetofauna establishment, in which an establishment probability scores category ranging from very low (1 < score < 2) to extremely high (9 < score ≤ 10) (for more details, see [71]). Due to the lack of information on the phylogenetic distance between these species and the Brazilian species, we used the second via of the spreadsheet to answer the following questions: What are the natural history characteristics of the species? What is the climate average for the species in the area of interest? What is the degree of relatedness of the species concerning native species? How many presentations of propagules will be made over the next five years? How old are the species when they reach reproductive maturity? How many offspring do the species produce per year? The climate average calculation was performed according to van Wilgen et al. [72]. Due to the lack of information and the difficulty of predicting the number of introductions that will be made over the next five years, we used a score of five for all species, as suggested by van Wilgen and Richardson [71].

Additionally, we used the results of potential distribution modeling and other factors that may influence the success of establishment of exotic species to analyze qualitatively the invasion risk of the species. According to Bomford et al. [11], although the relationship between these attributes and establishment success is properly proven, they should be considered in qualitative evaluations on invasion risk. The attributes examined were: Exceptional dispersion characteristics, high body mass index, high fertility, invasion history, general diet, ability to live in disturbed habitats and human commensalism, elsewhere damage report, disease vector and female’s ability to colonize alone. These attributes were selected based on the work of Bomford et al. [11] and Reed et al. [73].

## Results

**Fig 1:**
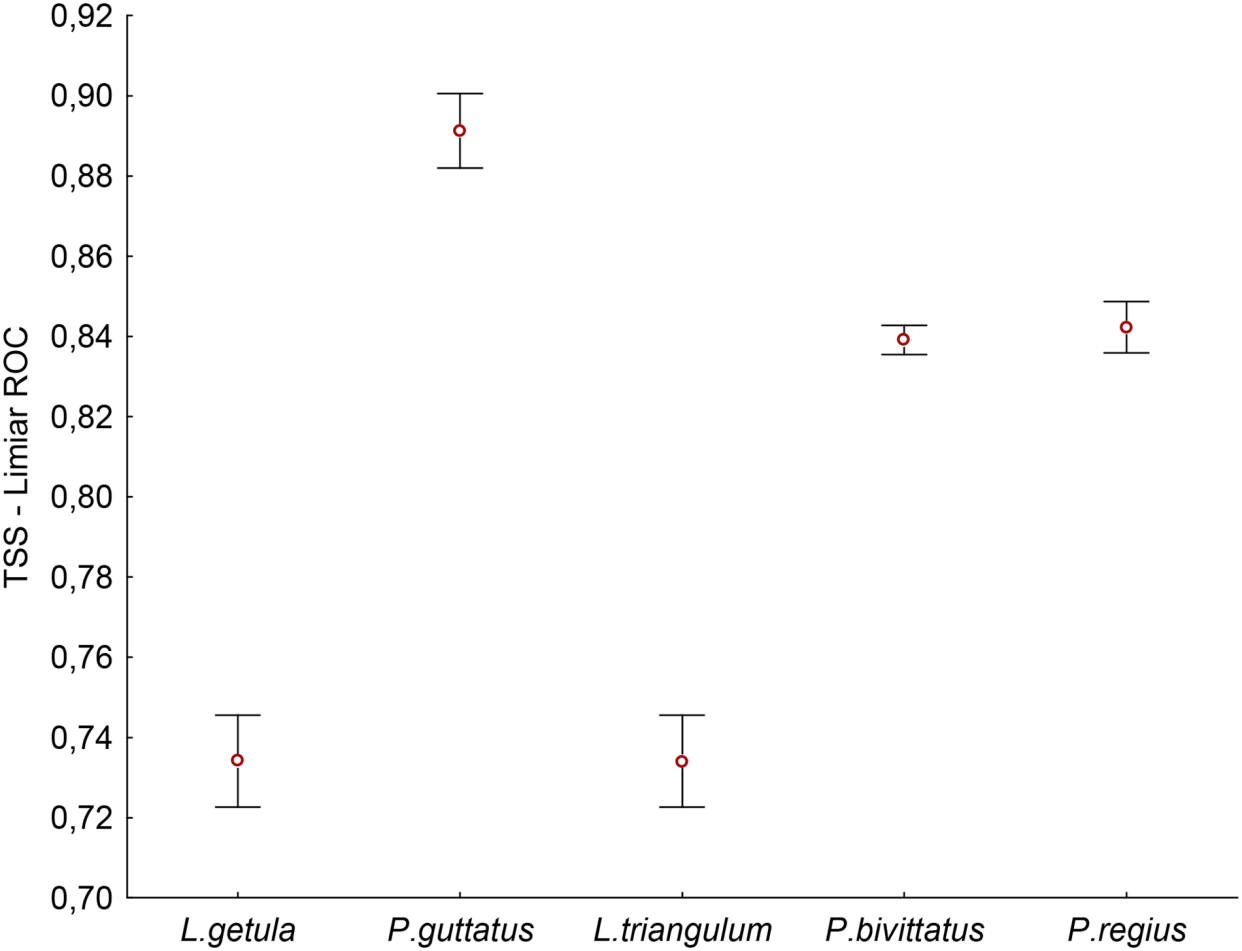
TSS values. TSS values (ROC threshold) for all models, with their respective means and 95% confidence interval.

**Fig 2:**
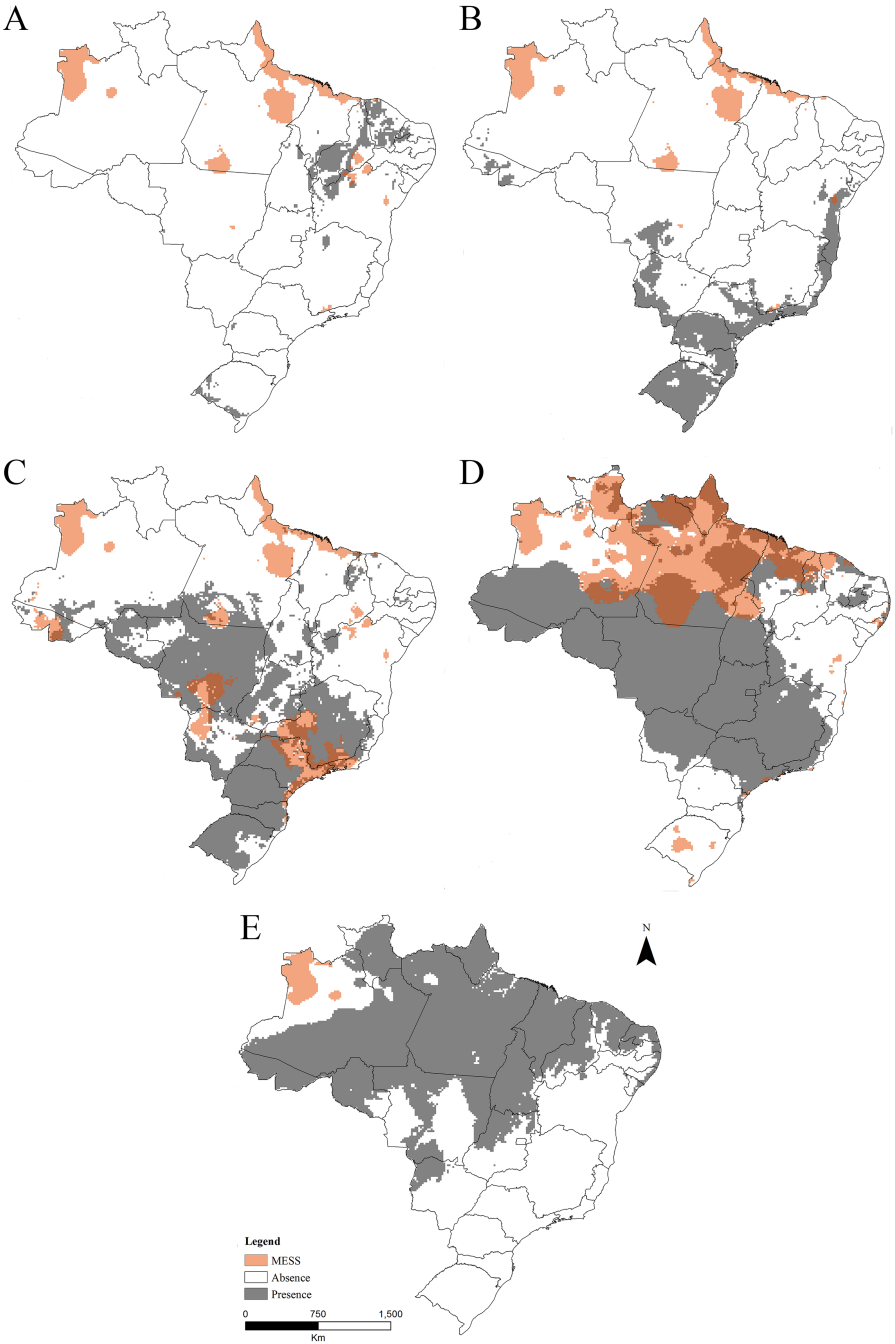
Potential distribution of the five species. (A) Potential distribution of *Lampropeltis getula*. (B) Potential distribution of *Pantherophis guttatus*. (C) Potential distribution of *Lampropeltis triangulum*. (D) Potential distribution of *Python bivittatus*. (E) Potential distribution of *Python regius*. Orange areas represent MESS regions with non-analogue climate.

Our results indicate that the performance of the models was satisfactory for all species, with TSS values always greater than 0.7 (Fig 1). TSS values were always smaller for *L*. *getula* and *L*. *triangulum* and greater for *P*. *guttatus*, which presented the best predictive performance. According to arrays of presence and absence, the potential distribution of *L*. *getula* was expected in some parts of the Northeast and Southern regions of Brazil, with small appropriate areas in the North and Southeast (Fig 2A). Appropriate areas for *P*. *guttatus* were found in regions of the Brazilian coast from the South to the Northeast, with some appropriate areas in Midwest, and a small portion in the Northwest (Fig 2B).

**Fig 3:**
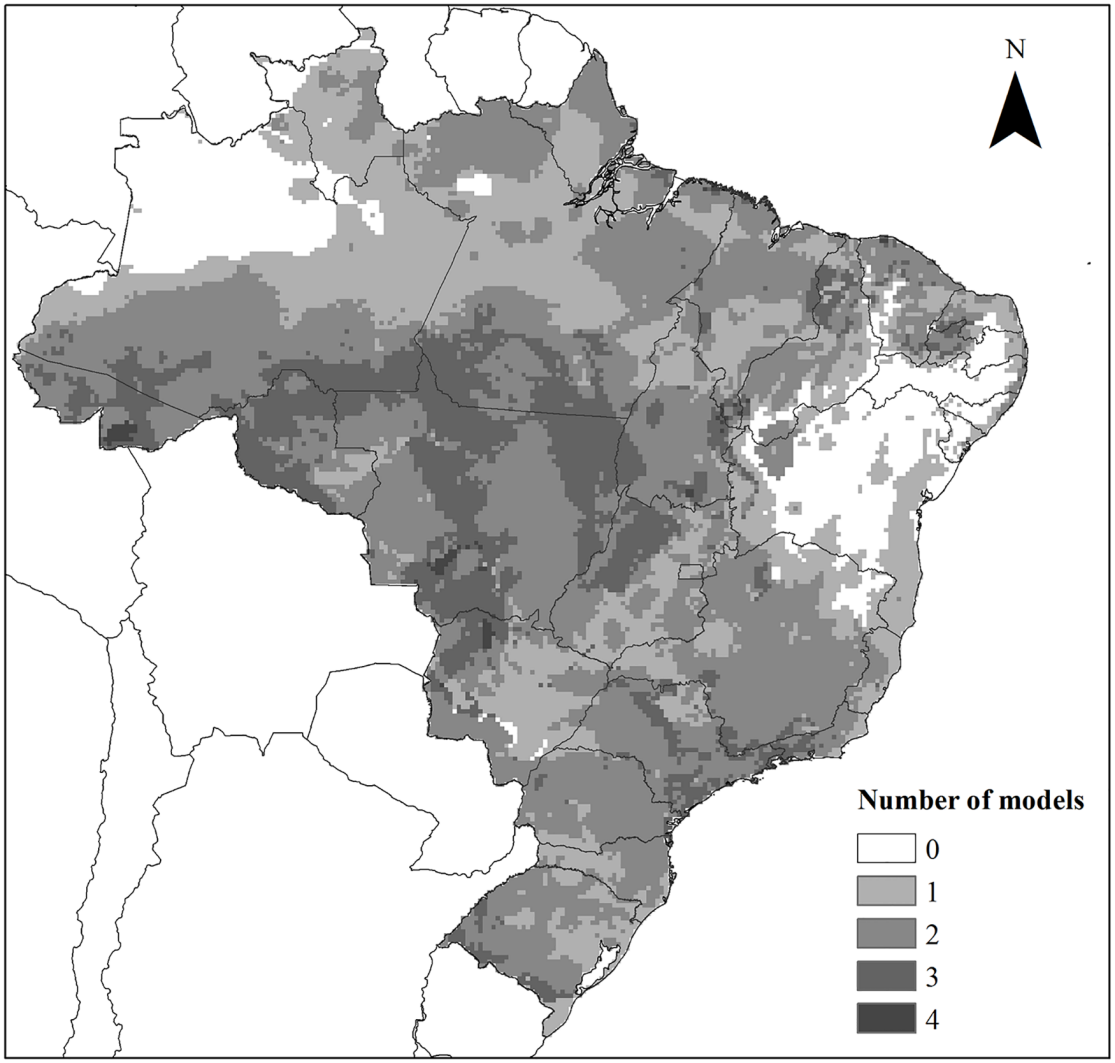
Consensus model of the potential distribution of the five species.

Our models indicated that the potential distribution of *L*. *triangulum* was higher for the South, Southeast and Midwest regions, with small fractions in the North and Northeast (Fig 2C). For *P*. *bivittatus*, these areas were referred to most of the Midwest, North and Southeast of the country, with some appropriate spots in the Northeast (Fig 2D). *Python regius*, on its turn, showed a broad potential distribution, located in the northern portion of Brazil and comprises mainly the Northern, Midwest and Northeast regions to a lesser proportion (Fig 2E). Appropriate locations for *P*. *regius* in the South and Southeast regions were not observed. The most propitious areas to establishment by these species are listed in the consensus model (Fig 3). Areas of greater suitability can be found in all Brazilian geographic regions, but mainly in the North and Midwest. Localities in the northwest regions, near the borders with Colombia and Venezuela, and much of the areas of Caatinga of the Brazilian Northeastern region, were considered areas of absence for all species.

The results of the SPHE Model indicate that the probability of establishment of the analyzed species ranged from high (4< score <7) to very high (7< score <8). This indicates that species identified with a high establishment probability have from 31 to 50% of chance of establishment. Those indicated with a very high probability had about 51 to 70% of chance of establishment. The information used in the model, references and total score of each species can be seen in Table 1.

**Table 1:** Success Probability of the Herpetofauna Establishment Model.

Species	Life form	Average climate match	Average phylogenetic distance	Number of introductions	Age at maturity (months)	Clutch size	TOTAL SCORE	References
*P .regius*	Snake	0.62	6	5	29	6	**7.36**	[74,75]
*P. bivittatus*	Snake	0.52	6	5	30	18	**7.18**	[73]
*L. getula*	Snake	0.28	2	5	36	13	**5.20**	[76,77]
*L. triangulum*	Snake	0.56	2	5	42	8	**5.56**	[78,79]
*P. guttatus*	Snake	0.49	2	5	20	11	**6.10**	[36]

Values used in the calculation and result of each species in the SPHE model.

Concerning the qualitative assessment of specific attributes, *P*. *bivitattus* and *P*. *gutattus* were the species with the highest number of attributes associated with a greater success of establishment, followed by *L*. *getula*, *L*. *triangulum* and *P*. *regius*, respectively (Table 2).

**Table 2:** Qualitative analysis of the establishment risk.

Attributes	Species				
	*P*. *regius*	*P*. *bivittatus*	*L*. *getula*	*L*. *triangulum*	*P*. *guttatus*
Ability to live in disturbed habitats and human commensalism	X	X		X	X
High body		X			
Broad diet		X	X	X	X
Features exceptional dispersion		X			
High fertility		X			
Single female able to colonize alone		X			
Invasion history		X	X		X
Negative impacts in other place		X	X		X
Vector diseases		X			X

## Discussion

### Model performance

The lower TSS values for *L*. *getula* and *L*. *triangulum* may be related to the broad geographic distribution of both species. Generalist species possess a variety of ecological attributes that characterize their niche and, therefore, the contrast between the areas of presence and absence becomes less pronounced, reducing the statistical accuracy of the model [80]. Similar results were found in other studies with other species of wide distribution, indicating that it is a common trend in niche modeling [80–82].

In contrast, the models of *P*. *guttatus* had the best predictive performance. The models of *P*. *guttatus* were the only ones in which also occurrence records from invaded areas were used, which may have improved the models’ performance. Some species are not able to disperse to suitable areas outside of their native realized distribution due to the presence of geographic barriers or due to biological interactions [83]. In these cases, their realized distribution does not include all of the areas with similar characteristics to the conditions of their fundamental niche. Thus, according to Jiménez-Valverde et al. [84], the use of all available information enables a more comprehensive characterization of the fundamental niche of the species, improving the prediction of the model. This may be therefore, the best way to predict the climatic viability for invasion of exotic species.

### Climatic viability and establishment risk

In *P*. *bivitattus* and *L*. *triagulum* suitable areas partially overlap MESS areas, indicating that in these regions at least one variable exceeded the training range. Predictions in these areas represent extrapolation and need to be interpreted with caution. The colonization, establishment and dispersion in potentially suitable areas involve a number of environmental and intrinsic factors of the species, many of which are not yet fully clarified [85]. On a local scale, factors such as environmental alterations, biotic interactions and the ability of dispersion may prevent the establishment of these species in climatically suitable areas [6,8]. Consequently, possible differences between realized and potential distributions can be observed. In addition, it is important to stress that some regions were suggested by the models as inadequate (areas of potential absence) which does not necessarily mean that the species can be deliberately introduced without any risk of invasion.

#### Lampropeltis getula

*Lampropeltis getula* is typically terrestrial and inhabits open forests, dams and coastal swamps, with some subspecies restricted to arid areas [86,87]. The areas where its presence was predicted cover three different Brazilian biomes (Pampa, Cerrado and Caatinga), which match their preference for open areas. Despite presenting the smallest potential distribution, the predicted area covers biologically important regions of Brazil, including a number of priority areas for conservation of the biological diversity of the Caatinga, some of extreme importance [88]. These areas harbour a high variety of species, high endemism rates, and presence of rare or endangered species [88].

According to the SPHE model, the success of establishment of this species is considered high (Score 5.2). However, it suggests the smallest size in terms of the potential distribution, and has few attributes related to establishment success. This species is an invader on the Gran Canaria Island in Spain [36], but little is known about its impact on the island. Mateo et al. [36] pointed out predation of endemic reptiles and small introduced mammals. This species has an opportunistic diet consisting of all vertebrate groups, mainly other snakes, including the venomous ones such as coral snakes (*Micrurus* spp.) and rattlesnakes (*Crotalus* spp.) [77]. Representatives of the two genera can be found in places where the potential distribution of *L*. *getula* was predicted [89].

#### Pantherophis guttatus

Its potential distribution was predicted mainly to Brazilian forested regions, most within the Atlantic forest domain. Within the area predicted for *P*. *guttatus*, the Serra do Mar is an extremely species rich region which is of great importance for the conservation of native and endemic species, as well as the Atlantic forest domain [90,91]. According to Eterovic and Duarte [49], *P*. *guttatus* has similar niche concerning diet, habits and reproduction to other snakes, such as *Bothrops jararaca* (Wied, 1824), *Oxyrhopus clathratus* Duméril, Bribon & Duméril 1854, and *Tropidodryas striaticeps* (Cope, 1869), which also inhabit the Serra do Mar [92], as well as invader species of the region, as *Crotalus durissus* Linnaeus, 1758 and *Oxyrhopus guibei* Hoge & Romano, 1977 [93]. This indicates that in this region, not only the climate appears to be appropriate, as there are also resources which are required by this species.

The result of the SPHE model indicated high probability of establishment of *P*. *guttatus* (Score 6.1). The ability to adapt to diverse environments was responsible for *P*. *guttatus* being taken to various parts of the world, through pet trade [36]. This snake invaded and established itself on the Caribbean Islands, and propagules have been found in Australia, South Africa, Brazil, Spain, Hawaii and the Bahamas [23,36,51,94–99]. In invaded environments, its presence could be a natural threat to populations of native birds [22,95], and other snakes due to the transmission of the parasite *Cryptosporidium* sp. [100]. It is known that individuals of this species are able to survive in urban and agricultural environments [36].

In Brazil, most of the breeding of these animals in captivity takes place within areas identified as suitable for *P*. *guttatus* [50]. According to Magalhães and São Pedro [50], *P*. *guttatus* has the highest percentage (43.37%) of pet advertisements sold illegally on the Internet in southeastern Brazil, with the largest amount of vendors detected between 2006 and 2012. Consequently, the risk of escape or release of propagules in these environments is greater, which requires the attention of the responsible agencies, especially regarding the monitoring of these areas.

#### Lampropeltis Triangulum

Appropriate areas for this species include mainly the biomes of the Atlantic forest, Cerrado and Pampa, and include various sustainable use conservation and full protection units, for instance, the Integral Protection Area (APA) of Ibirapuitã, one of two national protected areas of Pampa [101]; the APA of Islands and Valleys of the Paraná River, one of the largest of the Atlantic forest; and the APA of Rio Preto in the Cerrado. *Lampropeltis triangulum* has an extremely wide natural distribution [102,103] including a number of recognized subspecies, enabling greater adaptation to different types of habitats, which can be an aggravating factor in cases of introduction. In its native distribution, this species inhabits areas of tropical and subtropical moist forest, dry plains, rocky hillsides and agricultural areas [102,103].

The probability of establishment of this species in Brazil was considered high (Score: 5.56). The second largest percentage (9.4%) of ads of pets sold illegally on the Internet in southeastern Brazil belong to *L*. *triangulum*, with 39 sellers detected between 2006 and 2012 [50]. This species has some similarities to *Boiga irregularis* like: It is a terrestrial snake, of twilight and nocturnal habits [78]. It forages in search of small mammals, birds and their eggs, lizards, and smaller snakes and their eggs [104,105]. Additionally, like *P*. *guttatus*, it has niches similar to that of some species of Brazilian snakes, as some species of the genus *Micrurus*, *Apostolepis* and *Erythrolamprus* [49].

#### Python bivittatus

This species can be found in anthropogenic modified environments, including pastures [106], and has a generalist diet [107]. Its potential distribution includes Northern and Midwestern regions and covers virtually all river basins in the country, with the exception of the Uruguay and South Atlantic basins. This is a very important factor for the dispersal of this species which has terrestrial and semi-aquatic habits with high dispersal capacity through water [25]. The ability to disperse through aquatic environments appears to be related to a greater success of invasion for some animals [108].

The establishment probability was considered very high according to the SPHE model (Score: 7.18). *Python bivittatus* has practically all attributes related to the establishment success of the invasive populations analyzed in this study. It is an animal with high body mass, and high fecundity and longevity, factors that may be linked to a greater establishment success [25,73,107,109]. The species shows parental care with its eggs, which decreases the chances of predation and increases the survival of offspring [110]. Females of *P*. *bivittatus* are able to reproduce by parthenogenesis [111]. For this species, this type of reproduction contributes to the reduction of the number of viable propagules required to establish a population. The ability to reproduce by parthenogenesis has been singled out as the determining factor for the success of the invasion of species like *Indotyphlops braminus*, *Anolis sagrei* Duméril & Bibron, 1837 and *Lepidodactylus lugubris* (Duméril & Bibron, 1836) [108,112,113].

Additionally, *P*. *bivittatus* has a history of invasion and settlement in the Everglades National Park, Florida, USA [114]. The first record was made in 1979, and it is currently possible to observe the impacts of its introduction, as the intense predation on mammals and native birds [106,115]. In addition to increased predation, *P*. *bivittatus* also may be responsible for the transmission of exotic ticks [116]. In view of the potential distribution, establishment probability results, the attributes analyzed in this work and its popularity in pet trade, *P*. *bivittatus* was considered the species with greatest potential for invasion among all analyzed species.

#### Python regius

According to the ENM, appropriate areas were found mostly in the Amazonian domain. The Amazon has the largest biodiversity in the world [117], with the greatest number of reptiles of Brazil, the endemic majority of the region [118,119]. The presence of a new competitor or increased predation can be detrimental to these endemic species. Competitive displacement was observed in some invasions by reptiles, which led to damage to native species [120–124]. A similar situation occurs in relation to the impacts of predation by invasive reptiles, which can lead to a reduction in the rate of survival of native prey species [11]. As well as for *P*. *bivittatus*, the probability of establishment was regarded as very high (Score 7.36), the highest score among the analyzed species. Despite not having so many attributes linked to establishment success of invasive populations as its congener, its high score was mainly determined by the average value of the climate and the correspondence degree of kinship in relation to native species. According to van Wilgen and Richardson [71], reptiles more distantly related to native species have a higher probability to establish invasive populations. It is argued that not closely related species can benefit from a smaller influence of direct competitors; lower probability of predation and diseases; or even a higher possibility to occupy unexplored niches.

*Python regius* is typically terrestrial, feeding mostly on rodents and occasionally on birds and lizards [125]. It can coexist with the human presence [126], resulting in its wide dissemination as a pet, leading to the status “endangered” in its original habitat [126]. Due to this type of use, *P*. *regius* is one of the species with the highest number of illegal Internet sales ads in southeastern Brazil, with a total of 29 detected sellers from 2006 to 2012 [50]. In a niche analysis, Eterovic and Duarte [49] suggested that exotic Boidae and Pythonidae can be as successful as other invader snakes, and would use the same types of resources as native Viperidae.

### Potential areas of invasion

Areas more likely to be invaded by these species are: part of the Pantanal, Cerrado and Amazon, regions in which the anthropic action has been intensified in recent years [127–129]. This human interference can cause great damage to native species by favoring, indirectly, the invasion of species [130–133]. The exotic herpetofauna of Florida is directly associated to altered habitats, including agricultural and urban areas [9,134].

Environmental degradation makes the environment less resistant and impoverished, offering opportunities for the establishment of exotic species [135–137]. Following this same logic, richer and more stable environments seem to be harder to invade because of their biotic components, which are adapted to those conditions and have higher position to compete with possible invaders [136]. Some studies have shown that, for the herpetofauna, there seems to be a positive relationship between the richness of native congeners and the success of invasion [138,139,140]. In general, invasive species are more successful in places where the number of congeners is higher, probably because of the availability of resources and the similarities between native and non-native species, which allows for a pre-adaptation to the new local conditions [138]. In contrast, van Wilgen and Richardson [77] and Case and Bolger [141], found support for the hypothesis that the success of establishment decreases in environments where native congeners are present, since it would compete to explore similar niches. While there is no consensus on the effects of the presence of native species on the success of establishment of non-native congeners, the introductions of reptiles in South America did not show support for any of the two hypotheses [138]. The Northern and Midwestern regions are the main sectors for Brazilian agriculture and livestock farming [142]. This setting threatens local biodiversity due to the increased loss of habitat resulting from the expansion of these sectors [127–129]. In addition, like most releases occur in populated areas and most establishments start in disturbed areas [11] such as agricultural areas, it is possible to predict that this regional setting is an aggravating factor, since the environment is fragile and local species assemblages are likely disturbed.

To prevent the establishment of these exotic species, we suggest that the locations where most of the models pointed as suitable the permanence and invasion of these species receive special attention regarding monitoring. Moreover, in places where the potential distribution of species overlaps with areas of Conservation Units, it is necessary to train managers to recognize exotic species in order to identify the problem in advance. In addition to these measures, other care need to be taken, such as to discourage the breeding of exotic species, raise awareness about the risk of loosening, and intensify surveillance at the Brazilian borders and postal agencies, ports and airports. It is worth mentioning that the invasion of species does not occur solely through animals from other countries, but also from another region or biome [143,144,145]. In Brazil, despite breeding of some wild animals is allowed, the introduction of species beyond their original distribution area is prohibited by Decree (No. 6.514/2008).

Finally, even though there are no records of invasion of these species in Brazil, records of propagules of these species have been more frequent in the last years (unfortunately, mostly of them are not documented), showing that there is a propagule pressure whose total numbers are yet unknown, represented mainly by escapes or releases of animals used in the illegal pet trade. Data from an ongoing project suggests an unknown invasion on course or an imminent risk at certain regions of the country. Over the last 10 years, there are records of at least 329 individuals of these species in Brazil, 111 only in the state of São Paulo (Fonseca, unpublished data). The high pressure of propagules is crucial on sustaining an introduced population, enabling maintenance conditions for small populations, through the increasing genetic diversity and adaptation chances, as well as offering these individuals an opportunity to find a favorable environment to their establishment [146].

## Conclusion

The environmentally most suitable areas for the five analyzed snakes were found in the Northern and Midwestern Brazilian regions, including much of the Cerrado and the Amazon. Such regions rich in species numbers, which suffer severe degradation due to human actions. To prevent the introduction in these locations, an increase control and monitoring is necessary, mainly concerning *P*. *regius*, *P*. *guttatus* and *P*. *bivittatus*, identified with high invasion risk. The last two have practically all the requirements to become successful invaders, and are responsible for the decline of species in the environments invaded by them in other parts of the world. Appropriate areas for these snakes were predicted in regions which are extremely important for the Brazilian biodiversity, as the Atlantic forest and the Amazon, where attention must be intensified.

Our study provides a vision of the potential distribution of exotic species of snakes to Brazil, along with an assessment of their invasion risk. This approach provides an orientation to prevent the entry of potential invaders and prevent invasions in specific regions. The used procedure can be easily applied to other biological groups and should be used for risk analysis of invasion and for support of public policies.

## Supporting information

S1 Datasets Occurrence records of the five species (geographic coordinates). The dataset of occurrence records of *Lampropeltis getula* in North America and Central America (S1A Dataset). Records of *Pantherophis guttatus* in North America and occurrence records from invaded areas (S1B Dataset). Records of *Lampropeltis triangulum* in the Americas (S1C Dataset). Records of *Python bivittatus* in Asia (S1D Dataset). Occurrence records of *Python regius* in Africa (S1E Dataset).
(DOCX)

S1 Tables Environmental variables and their respective PCA axes used as environmental layers. S1A Table—Environmental variables and their respective PCA axes used as environmental layers for American species. S1B Table—Environmental variables and their respective PCA axes used as environmental layers for the Asian species. S1C Table—Environmental variables and their respective PCA axes used as environmental layers for the African species.
(DOCX)
