# Supplementary material for: Pet snakes illegally marketed in Brazil: Climatic viability and establishment risk
Source: PLoS One. 2017 Aug 17;12(8):e0183143. doi: 10.1371/journal.pone.0183143 (PMC5560532; doi:10.1371/journal.pone.0183143)
Supplement: S1 Datasets — The dataset of occurrence records of Lampropeltis getula in North America and Central America (S1A Dataset). Records of Pantherophis guttatus in North America and occurrence records from invaded areas (S1B Dataset). Records of Lampropeltis triangulum in the Americas (S1C Dataset). Records of Python bivittatus in Asia (S1D Dataset). Occurrence records of Python regius in Africa (S1E Dataset). (DOCX) [file pone.0183143.s001.docx]

**S1 Datasets - Occurrence records of the five species (geographic coordinates).**

**S1A Dataset**

Occurrence records of *Lampropeltis getula* in North America and Central America.

| Specie | Longitude | Latitude |
| --- | --- | --- |
| *Lampropeltis getula* | -99.16 | 20.1 |
| *Lampropeltis getula* | -109.70375 | 23.05569 |
| *Lampropeltis getula* | -99.62 | 23.261 |
| *Lampropeltis getula* | -106.11667 | 23.26667 |
| *Lampropeltis getula* | -109.77444 | 23.36694 |
| *Lampropeltis getula* | -109.84391 | 23.39038 |
| *Lampropeltis getula* | -109.70672 | 23.44289 |
| *Lampropeltis getula* | -110.01822 | 23.44843 |
| *Lampropeltis getula* | -110.21667 | 23.45 |
| *Lampropeltis getula* | -109.71667 | 23.48333 |
| *Lampropeltis getula* | -110.067 | 23.50139 |
| *Lampropeltis getula* | -109.65842 | 23.62103 |
| *Lampropeltis getula* | -110.09937 | 23.6604 |
| *Lampropeltis getula* | -100.39 | 23.69 |
| *Lampropeltis getula* | -109.94 | 23.75 |
| *Lampropeltis getula* | -110.03 | 23.76 |
| *Lampropeltis getula* | -110.02287 | 23.78715 |
| *Lampropeltis getula* | -110.116667 | 23.8125 |
| *Lampropeltis getula* | -110.25 | 23.82 |
| *Lampropeltis getula* | -110.11667 | 23.82556 |
| *Lampropeltis getula* | -110.26111 | 23.90833 |
| *Lampropeltis getula* | -110.259722 | 23.941667 |
| *Lampropeltis getula* | -110.310833 | 24.090278 |
| *Lampropeltis getula* | -104.605548 | 24.096764 |
| *Lampropeltis getula* | -110.38 | 24.1 |
| *Lampropeltis getula* | -110.31083 | 24.12528 |
| *Lampropeltis getula* | -104.7025 | 24.18843 |
| *Lampropeltis getula* | -104 | 24.52 |
| *Lampropeltis getula* | -80.6375 | 25.3505 |
| *Lampropeltis getula* | -80.3507 | 25.6877 |
| *Lampropeltis getula* | -80.94029 | 25.74828 |
| *Lampropeltis getula* | -81.03272 | 25.76007 |
| *Lampropeltis getula* | -80.3816 | 25.7606 |
| *Lampropeltis getula* | -81.00054 | 25.76062 |
| *Lampropeltis getula* | -80.87238 | 25.76085 |
| *Lampropeltis getula* | -80.7527 | 25.7619 |
| *Lampropeltis getula* | -108.9 | 25.79 |
| *Lampropeltis getula* | -111.5447 | 25.81846 |
| *Lampropeltis getula* | -102.97 | 25.82 |
| *Lampropeltis getula* | -81.1017 | 25.8625 |
| *Lampropeltis getula* | -81.3036 | 25.9011 |
| *Lampropeltis getula* | -80.43722 | 26.07134 |
| *Lampropeltis getula* | -80.106483 | 26.148644 |
| *Lampropeltis getula* | -109.03 | 26.33 |
| *Lampropeltis getula* | -104.04 | 26.42 |
| *Lampropeltis getula* | -104.16 | 26.55 |
| *Lampropeltis getula* | -81.7188 | 26.5849 |
| *Lampropeltis getula* | -109.17 | 26.59 |
| *Lampropeltis getula* | -81.886 | 26.664 |
| *Lampropeltis getula* | -80.693196 | 26.692511 |
| *Lampropeltis getula* | -80.57076 | 26.69676 |
| *Lampropeltis getula* | -80.036419 | 26.705639 |
| *Lampropeltis getula* | -80.55455 | 26.70702 |
| *Lampropeltis getula* | -109.32542 | 26.77009 |
| *Lampropeltis getula* | -81.0657 | 26.8122 |
| *Lampropeltis getula* | -81.02288 | 26.82648 |
| *Lampropeltis getula* | -109.43 | 26.98 |
| *Lampropeltis getula* | -102.05 | 26.99 |
| *Lampropeltis getula* | -101.94 | 27 |
| *Lampropeltis getula* | -109.510774 | 27.014524 |
| *Lampropeltis getula* | -108.93333 | 27.01667 |
| *Lampropeltis getula* | -108.95 | 27.03 |
| *Lampropeltis getula* | -80.9885 | 27.0482 |
| *Lampropeltis getula* | -109.44 | 27.05 |
| *Lampropeltis getula* | -109.37 | 27.07 |
| *Lampropeltis getula* | -109.32 | 27.08 |
| *Lampropeltis getula* | -109.43364 | 27.08083 |
| *Lampropeltis getula* | -109.1 | 27.11667 |
| *Lampropeltis getula* | -99.654 | 27.209 |
| *Lampropeltis getula* | -112.89583 | 27.28194 |
| *Lampropeltis getula* | -113.02 | 27.28239 |
| *Lampropeltis getula* | -99.536 | 27.38 |
| *Lampropeltis getula* | -112.94167 | 27.39333 |
| *Lampropeltis getula* | -98.649017 | 27.496838 |
| *Lampropeltis getula* | -99.05225 | 27.64674 |
| *Lampropeltis getula* | -80.651039 | 27.676186 |
| *Lampropeltis getula* | -97.44735 | 27.70281 |
| *Lampropeltis getula* | -82.443 | 27.724 |
| *Lampropeltis getula* | -82.67961 | 27.77112 |
| *Lampropeltis getula* | -80.475158 | 27.817187 |
| *Lampropeltis getula* | -82.675 | 27.899 |
| *Lampropeltis getula* | -82.36847 | 27.90797 |
| *Lampropeltis getula* | -82.74122 | 27.91631 |
| *Lampropeltis getula* | -82.4402 | 27.97333 |
| *Lampropeltis getula* | -97.054511 | 28.020597 |
| *Lampropeltis getula* | -81.94981 | 28.03946 |
| *Lampropeltis getula* | -97.36795 | 28.10713 |
| *Lampropeltis getula* | -97.509 | 28.14492 |
| *Lampropeltis getula* | -81.408192 | 28.289782 |
| *Lampropeltis getula* | -113.80967 | 28.3068 |
| *Lampropeltis getula* | -106.13 | 28.57 |
| *Lampropeltis getula* | -112.759 | 28.596 |
| *Lampropeltis getula* | -112.84 | 28.644 |
| *Lampropeltis getula* | -96.665134 | 28.647879 |
| *Lampropeltis getula* | -110.9964 | 28.6667 |
| *Lampropeltis getula* | -106.18 | 28.67 |
| *Lampropeltis getula* | -112.882 | 28.683 |
| *Lampropeltis getula* | -106.148741 | 28.727559 |
| *Lampropeltis getula* | -82.29676 | 28.74999 |
| *Lampropeltis getula* | -81.27312 | 28.80055 |
| *Lampropeltis getula* | -98.547004 | 28.813589 |
| *Lampropeltis getula* | -82.61667 | 28.83333 |
| *Lampropeltis getula* | -105.85 | 28.86 |
| *Lampropeltis getula* | -99.717003 | 28.938128 |
| *Lampropeltis getula* | -98.5724 | 28.96459 |
| *Lampropeltis getula* | -81.444 | 29.057 |
| *Lampropeltis getula* | -105.54 | 29.12 |
| *Lampropeltis getula* | -103.58235 | 29.15323 |
| *Lampropeltis getula* | -102.955 | 29.1799 |
| *Lampropeltis getula* | -98.68172 | 29.20964 |
| *Lampropeltis getula* | -103.01852 | 29.21199 |
| *Lampropeltis getula* | -82.05782 | 29.21646 |
| *Lampropeltis getula* | -89.993606 | 29.235175 |
| *Lampropeltis getula* | -113.34564 | 29.24467 |
| *Lampropeltis getula* | -90.739506 | 29.281923 |
| *Lampropeltis getula* | -90.702445 | 29.288189 |
| *Lampropeltis getula* | -95.127964 | 29.399895 |
| *Lampropeltis getula* | -82.22 | 29.42 |
| *Lampropeltis getula* | -106.33048 | 29.46218 |
| *Lampropeltis getula* | -99.53233 | 29.4675 |
| *Lampropeltis getula* | -106.40168 | 29.4802 |
| *Lampropeltis getula* | -95.913499 | 29.527124 |
| *Lampropeltis getula* | -82.52 | 29.55 |
| *Lampropeltis getula* | -82.25336 | 29.5655 |
| *Lampropeltis getula* | -82.253366 | 29.565505 |
| *Lampropeltis getula* | -98.47917 | 29.57648 |
| *Lampropeltis getula* | -82.3351 | 29.5774 |
| *Lampropeltis getula* | -82.312629 | 29.577939 |
| *Lampropeltis getula* | -114.56574 | 29.59122 |
| *Lampropeltis getula* | -110.94 | 29.63 |
| *Lampropeltis getula* | -103.07476 | 29.63755 |
| *Lampropeltis getula* | -89.939762 | 29.64633 |
| *Lampropeltis getula* | -82.32448 | 29.6514 |
| *Lampropeltis getula* | -82.3525 | 29.6818 |
| *Lampropeltis getula* | -90.793168 | 29.696106 |
| *Lampropeltis getula* | -101.20242 | 29.69988 |
| *Lampropeltis getula* | -90.925926 | 29.700881 |
| *Lampropeltis getula* | -82.3932 | 29.70686 |
| *Lampropeltis getula* | -95.062126 | 29.779769 |
| *Lampropeltis getula* | -93.489158 | 29.792426 |
| *Lampropeltis getula* | -114.41045 | 29.81212 |
| *Lampropeltis getula* | -94.033785 | 29.825948 |
| *Lampropeltis getula* | -111.06355 | 29.82818 |
| *Lampropeltis getula* | -111.1 | 29.83 |
| *Lampropeltis getula* | -91.424754 | 29.849079 |
| *Lampropeltis getula* | -84.990501 | 29.870992 |
| *Lampropeltis getula* | -89.987357 | 29.902424 |
| *Lampropeltis getula* | -90.150433 | 29.916531 |
| *Lampropeltis getula* | -106.50438 | 29.92497 |
| *Lampropeltis getula* | -81.34098 | 29.95848 |
| *Lampropeltis getula* | -90.07007 | 29.9622 |
| *Lampropeltis getula* | -106.37 | 29.98 |
| *Lampropeltis getula* | -90.378331 | 29.99215 |
| *Lampropeltis getula* | -90.4413 | 30.0019 |
| *Lampropeltis getula* | -84.502781 | 30.002588 |
| *Lampropeltis getula* | -90.326679 | 30.005563 |
| *Lampropeltis getula* | -91.013006 | 30.008385 |
| *Lampropeltis getula* | -90.404632 | 30.031362 |
| *Lampropeltis getula* | -90.560553 | 30.042284 |
| *Lampropeltis getula* | -115.73333 | 30.05 |
| *Lampropeltis getula* | -90.673538 | 30.093779 |
| *Lampropeltis getula* | -115.66111 | 30.095 |
| *Lampropeltis getula* | -94.64475 | 30.131767 |
| *Lampropeltis getula* | -96.249277 | 30.136744 |
| *Lampropeltis getula* | -103.2375 | 30.1648 |
| *Lampropeltis getula* | -115.78333 | 30.18333 |
| *Lampropeltis getula* | -85.659905 | 30.193892 |
| *Lampropeltis getula* | -89.087 | 30.219 |
| *Lampropeltis getula* | -91.986573 | 30.221612 |
| *Lampropeltis getula* | -115.78861 | 30.23056 |
| *Lampropeltis getula* | -89.647805 | 30.236006 |
| *Lampropeltis getula* | -99.87 | 30.25 |
| *Lampropeltis getula* | -102.74893 | 30.26978 |
| *Lampropeltis getula* | -94.49831 | 30.28995 |
| *Lampropeltis getula* | -91.501447 | 30.311253 |
| *Lampropeltis getula* | -91.786426 | 30.31421 |
| *Lampropeltis getula* | -106.51 | 30.33 |
| *Lampropeltis getula* | -88.148 | 30.347 |
| *Lampropeltis getula* | -92.300936 | 30.349824 |
| *Lampropeltis getula* | -114.64 | 30.35 |
| *Lampropeltis getula* | -107.9918 | 30.365543 |
| *Lampropeltis getula* | -90.57383 | 30.37829 |
| *Lampropeltis getula* | -91.165417 | 30.403476 |
| *Lampropeltis getula* | -89.03147 | 30.40686 |
| *Lampropeltis getula* | -87.217 | 30.42 |
| *Lampropeltis getula* | -84.204569 | 30.428283 |
| *Lampropeltis getula* | -91.419487 | 30.437947 |
| *Lampropeltis getula* | -91.253853 | 30.443632 |
| *Lampropeltis getula* | -84.32948 | 30.45911 |
| *Lampropeltis getula* | -115.9107 | 30.46047 |
| *Lampropeltis getula* | -90.101567 | 30.475028 |
| *Lampropeltis getula* | -90.693168 | 30.503157 |
| *Lampropeltis getula* | -92.390442 | 30.513371 |
| *Lampropeltis getula* | -103.7889 | 30.5167 |
| *Lampropeltis getula* | -93.056682 | 30.517182 |
| *Lampropeltis getula* | -115.96472 | 30.53464 |
| *Lampropeltis getula* | -115.96472 | 30.53464 |
| *Lampropeltis getula* | -91.829349 | 30.556899 |
| *Lampropeltis getula* | -104.45945 | 30.55713 |
| *Lampropeltis getula* | -90.425094 | 30.557563 |
| *Lampropeltis getula* | -84.292696 | 30.561739 |
| *Lampropeltis getula* | -104.50312 | 30.59008 |
| *Lampropeltis getula* | -91.239855 | 30.612914 |
| *Lampropeltis getula* | -94.104995 | 30.623482 |
| *Lampropeltis getula* | -96.334 | 30.628 |
| *Lampropeltis getula* | -89.2357 | 30.6356 |
| *Lampropeltis getula* | -112.21 | 30.64 |
| *Lampropeltis getula* | -96.31392 | 30.66693 |
| *Lampropeltis getula* | -87.75306 | 30.69034 |
| *Lampropeltis getula* | -91.300134 | 30.724421 |
| *Lampropeltis getula* | -90.973259 | 30.724425 |
| *Lampropeltis getula* | -85.683461 | 30.792425 |
| *Lampropeltis getula* | -87.907517 | 30.805445 |
| *Lampropeltis getula* | -82.39194 | 30.81806 |
| *Lampropeltis getula* | -92.266212 | 30.818821 |
| *Lampropeltis getula* | -89.508008 | 30.81891 |
| *Lampropeltis getula* | -93.28906 | 30.84629 |
| *Lampropeltis getula* | -81.454336 | 30.854156 |
| *Lampropeltis getula* | -115.88 | 30.89 |
| *Lampropeltis getula* | -102.88303 | 30.8904 |
| *Lampropeltis getula* | -102.86105 | 30.89303 |
| *Lampropeltis getula* | -83.984304 | 30.910298 |
| *Lampropeltis getula* | -103.79086 | 30.92773 |
| *Lampropeltis getula* | -116.01667 | 30.95 |
| *Lampropeltis getula* | -101.16139 | 30.96019 |
| *Lampropeltis getula* | -115.86032 | 30.96628 |
| *Lampropeltis getula* | -91.55396 | 30.967192 |
| *Lampropeltis getula* | -115.74 | 30.97167 |
| *Lampropeltis getula* | -116.15 | 30.98333 |
| *Lampropeltis getula* | -92.055929 | 30.984005 |
| *Lampropeltis getula* | -95.567293 | 31.003927 |
| *Lampropeltis getula* | -92.285662 | 31.014295 |
| *Lampropeltis getula* | -82.73506 | 31.025152 |
| *Lampropeltis getula* | -116.20162 | 31.08217 |
| *Lampropeltis getula* | -87.49389 | 31.09589 |
| *Lampropeltis getula* | -115.77495 | 31.09599 |
| *Lampropeltis getula* | -116.13167 | 31.12667 |
| *Lampropeltis getula* | -105.67025 | 31.14033 |
| *Lampropeltis getula* | -105.6841 | 31.14825 |
| *Lampropeltis getula* | -83.546019 | 31.151997 |
| *Lampropeltis getula* | -94.427154 | 31.158754 |
| *Lampropeltis getula* | -89.73309 | 31.17416 |
| *Lampropeltis getula* | -81.392 | 31.177 |
| *Lampropeltis getula* | -105.79798 | 31.23817 |
| *Lampropeltis getula* | -99.85995 | 31.244109 |
| *Lampropeltis getula* | -86.127654 | 31.276699 |
| *Lampropeltis getula* | -116.394 | 31.288 |
| *Lampropeltis getula* | -105.8519 | 31.28803 |
| *Lampropeltis getula* | -88.14818 | 31.29042 |
| *Lampropeltis getula* | -116.22692 | 31.30676 |
| *Lampropeltis getula* | -105.89799 | 31.31703 |
| *Lampropeltis getula* | -116.24378 | 31.32375 |
| *Lampropeltis getula* | -81.448217 | 31.329359 |
| *Lampropeltis getula* | -84.33028 | 31.32996 |
| *Lampropeltis getula* | -116.435 | 31.338 |
| *Lampropeltis getula* | -108.66 | 31.34 |
| *Lampropeltis getula* | -84.25415 | 31.351 |
| *Lampropeltis getula* | -112.61 | 31.37 |
| *Lampropeltis getula* | -110.03413 | 31.37943 |
| *Lampropeltis getula* | -110.16926 | 31.37993 |
| *Lampropeltis getula* | -110.11083 | 31.38 |
| *Lampropeltis getula* | -109.54514 | 31.38164 |
| *Lampropeltis getula* | -108.9 | 31.4 |
| *Lampropeltis getula* | -109.5005 | 31.40623 |
| *Lampropeltis getula* | -108.56 | 31.42 |
| *Lampropeltis getula* | -100.446718 | 31.456471 |
| *Lampropeltis getula* | -109.45762 | 31.45658 |
| *Lampropeltis getula* | -109.6115 | 31.4578 |
| *Lampropeltis getula* | -109.365108 | 31.461614 |
| *Lampropeltis getula* | -83.5063 | 31.4654 |
| *Lampropeltis getula* | -88.2301 | 31.4702 |
| *Lampropeltis getula* | -110.630899 | 31.470947 |
| *Lampropeltis getula* | -81.22 | 31.484 |
| *Lampropeltis getula* | -108.87 | 31.49 |
| *Lampropeltis getula* | -110.85444 | 31.49472 |
| *Lampropeltis getula* | -86.98283 | 31.50163 |
| *Lampropeltis getula* | -109.2868 | 31.5398 |
| *Lampropeltis getula* | -116.657 | 31.542 |
| *Lampropeltis getula* | -116.2031 | 31.55218 |
| *Lampropeltis getula* | -116.41571 | 31.56389 |
| *Lampropeltis getula* | -86.286935 | 31.571356 |
| *Lampropeltis getula* | -109.25075 | 31.58395 |
| *Lampropeltis getula* | -83.862714 | 31.603324 |
| *Lampropeltis getula* | -106.23548 | 31.6136 |
| *Lampropeltis getula* | -86.13662 | 31.65155 |
| *Lampropeltis getula* | -91.51856 | 31.65556 |
| *Lampropeltis getula* | -109.1617 | 31.6646 |
| *Lampropeltis getula* | -110.8867 | 31.6727 |
| *Lampropeltis getula* | -105.37109 | 31.68728 |
| *Lampropeltis getula* | -82.80421 | 31.68981 |
| *Lampropeltis getula* | -116.55304 | 31.6999 |
| *Lampropeltis getula* | -100.067967 | 31.701919 |
| *Lampropeltis getula* | -111.17378 | 31.70987 |
| *Lampropeltis getula* | -109.1143 | 31.7162 |
| *Lampropeltis getula* | -116.618 | 31.717 |
| *Lampropeltis getula* | -111.13694 | 31.72214 |
| *Lampropeltis getula* | -108.80667 | 31.728287 |
| *Lampropeltis getula* | -108.46 | 31.73 |
| *Lampropeltis getula* | -81.441403 | 31.744597 |
| *Lampropeltis getula* | -111.48235 | 31.74478 |
| *Lampropeltis getula* | -109.68843 | 31.74576 |
| *Lampropeltis getula* | -110.10354 | 31.74817 |
| *Lampropeltis getula* | -116.58536 | 31.75442 |
| *Lampropeltis getula* | -105.36076 | 31.7602 |
| *Lampropeltis getula* | -99.452999 | 31.763903 |
| *Lampropeltis getula* | -105.48269 | 31.77692 |
| *Lampropeltis getula* | -109.0706 | 31.7782 |
| *Lampropeltis getula* | -105.51589 | 31.78802 |
| *Lampropeltis getula* | -109.944505 | 31.790901 |
| *Lampropeltis getula* | -106.5707 | 31.81422 |
| *Lampropeltis getula* | -85.355023 | 31.817095 |
| *Lampropeltis getula* | -107.65153 | 31.82427 |
| *Lampropeltis getula* | -109.0482 | 31.8338 |
| *Lampropeltis getula* | -110.176611 | 31.836017 |
| *Lampropeltis getula* | -107.90639 | 31.83632 |
| *Lampropeltis getula* | -105.75381 | 31.83662 |
| *Lampropeltis getula* | -106.61298 | 31.85435 |
| *Lampropeltis getula* | -106.05181 | 31.86815 |
| *Lampropeltis getula* | -109.0406 | 31.87062 |
| *Lampropeltis getula* | -106.63133 | 31.87115 |
| *Lampropeltis getula* | -106.64929 | 31.87171 |
| *Lampropeltis getula* | -111.3962 | 31.87815 |
| *Lampropeltis getula* | -116.32033 | 31.8867 |
| *Lampropeltis getula* | -108.80667 | 31.887934 |
| *Lampropeltis getula* | -90.410992 | 31.890631 |
| *Lampropeltis getula* | -106.04823 | 31.89818 |
| *Lampropeltis getula* | -80.982836 | 31.905442 |
| *Lampropeltis getula* | -104.8 | 31.91 |
| *Lampropeltis getula* | -109.0319 | 31.9135 |
| *Lampropeltis getula* | -109.0592 | 31.9136 |
| *Lampropeltis getula* | -109.1408 | 31.9223 |
| *Lampropeltis getula* | -102.693427 | 31.927023 |
| *Lampropeltis getula* | -109 | 31.93 |
| *Lampropeltis getula* | -108.81 | 31.93 |
| *Lampropeltis getula* | -81.047028 | 31.934464 |
| *Lampropeltis getula* | -108.32998 | 31.93843 |
| *Lampropeltis getula* | -108.9425 | 31.93972 |
| *Lampropeltis getula* | -108.97 | 31.94 |
| *Lampropeltis getula* | -109.90958 | 31.94868 |
| *Lampropeltis getula* | -108.798118 | 31.94889 |
| *Lampropeltis getula* | -98.73734 | 31.949 |
| *Lampropeltis getula* | -108.76 | 31.95 |
| *Lampropeltis getula* | -106.603 | 31.95117 |
| *Lampropeltis getula* | -109.03849 | 31.97301 |
| *Lampropeltis getula* | -109.85421 | 31.98663 |
| *Lampropeltis getula* | -81.283982 | 31.986913 |
| *Lampropeltis getula* | -82.64215 | 31.99264 |
| *Lampropeltis getula* | -108.8 | 32 |
| *Lampropeltis getula* | -80.846914 | 32.001799 |
| *Lampropeltis getula* | -108.85 | 32.01 |
| *Lampropeltis getula* | -106.44476 | 32.03788 |
| *Lampropeltis getula* | -112.84069 | 32.06031 |
| *Lampropeltis getula* | -104.49 | 32.08 |
| *Lampropeltis getula* | -81.0965 | 32.0823 |
| *Lampropeltis getula* | -108.87861 | 32.08833 |
| *Lampropeltis getula* | -81.28718 | 32.094345 |
| *Lampropeltis getula* | -87.869753 | 32.095303 |
| *Lampropeltis getula* | -93.698188 | 32.098599 |
| *Lampropeltis getula* | -116.81048 | 32.10278 |
| *Lampropeltis getula* | -111.0073 | 32.1073 |
| *Lampropeltis getula* | -106.69621 | 32.11167 |
| *Lampropeltis getula* | -111.10987 | 32.13342 |
| *Lampropeltis getula* | -107.75 | 32.14 |
| *Lampropeltis getula* | -111.15071 | 32.14055 |
| *Lampropeltis getula* | -81.11378 | 32.15048 |
| *Lampropeltis getula* | -109.1748 | 32.1533 |
| *Lampropeltis getula* | -115.79333 | 32.155 |
| *Lampropeltis getula* | -110.97675 | 32.1575 |
| *Lampropeltis getula* | -92.131364 | 32.159037 |
| *Lampropeltis getula* | -111.2172 | 32.173 |
| *Lampropeltis getula* | -109.6659 | 32.18304 |
| *Lampropeltis getula* | -108.30602 | 32.1926 |
| *Lampropeltis getula* | -89.40852 | 32.19907 |
| *Lampropeltis getula* | -104.2 | 32.2 |
| *Lampropeltis getula* | -109.7686 | 32.2026 |
| *Lampropeltis getula* | -108.94974 | 32.20289 |
| *Lampropeltis getula* | -112.5899 | 32.2077 |
| *Lampropeltis getula* | -116.9 | 32.209 |
| *Lampropeltis getula* | -110.915249 | 32.210871 |
| *Lampropeltis getula* | -88.144093 | 32.216692 |
| *Lampropeltis getula* | -106.25 | 32.217 |
| *Lampropeltis getula* | -106.56845 | 32.2209 |
| *Lampropeltis getula* | -104.08 | 32.23 |
| *Lampropeltis getula* | -111.2172 | 32.2327 |
| *Lampropeltis getula* | -110.75173 | 32.23553 |
| *Lampropeltis getula* | -110.91944 | 32.23917 |
| *Lampropeltis getula* | -92.71378 | 32.24092 |
| *Lampropeltis getula* | -107.49289 | 32.24279 |
| *Lampropeltis getula* | -107.529 | 32.243 |
| *Lampropeltis getula* | -93.971985 | 32.250812 |
| *Lampropeltis getula* | -110.0192 | 32.2528 |
| *Lampropeltis getula* | -106.83804 | 32.27825 |
| *Lampropeltis getula* | -110.92365 | 32.28558 |
| *Lampropeltis getula* | -111.042045 | 32.285864 |
| *Lampropeltis getula* | -109.87577 | 32.28665 |
| *Lampropeltis getula* | -110.76211 | 32.28722 |
| *Lampropeltis getula* | -107.74 | 32.29 |
| *Lampropeltis getula* | -83.486619 | 32.294233 |
| *Lampropeltis getula* | -109.80843 | 32.29817 |
| *Lampropeltis getula* | -92.7013 | 32.29998 |
| *Lampropeltis getula* | -117.019 | 32.301 |
| *Lampropeltis getula* | -92.64517 | 32.30856 |
| *Lampropeltis getula* | -107.20707 | 32.31149 |
| *Lampropeltis getula* | -106.812123 | 32.312215 |
| *Lampropeltis getula* | -106.77778 | 32.31222 |
| *Lampropeltis getula* | -106.949461 | 32.312273 |
| *Lampropeltis getula* | -107.67 | 32.32 |
| *Lampropeltis getula* | -85.174938 | 32.324437 |
| *Lampropeltis getula* | -92.80436 | 32.3313 |
| *Lampropeltis getula* | -109.87653 | 32.33138 |
| *Lampropeltis getula* | -92.70249 | 32.34072 |
| *Lampropeltis getula* | -90.370922 | 32.341037 |
| *Lampropeltis getula* | -112.8249 | 32.3551 |
| *Lampropeltis getula* | -104.10471 | 32.35709 |
| *Lampropeltis getula* | -107.63 | 32.36 |
| *Lampropeltis getula* | -107.63 | 32.36 |
| *Lampropeltis getula* | -104.17 | 32.36 |
| *Lampropeltis getula* | -85.758251 | 32.362393 |
| *Lampropeltis getula* | -84.9651 | 32.3629 |
| *Lampropeltis getula* | -117.0571 | 32.37061 |
| *Lampropeltis getula* | -111.0917 | 32.375 |
| *Lampropeltis getula* | -82.316545 | 32.390217 |
| *Lampropeltis getula* | -117.0511 | 32.39891 |
| *Lampropeltis getula* | -117.083 | 32.399 |
| *Lampropeltis getula* | -93.05044 | 32.401159 |
| *Lampropeltis getula* | -81.81248 | 32.42313 |
| *Lampropeltis getula* | -109.97799 | 32.43039 |
| *Lampropeltis getula* | -81.414146 | 32.433232 |
| *Lampropeltis getula* | -91.746676 | 32.44392 |
| *Lampropeltis getula* | -86.459748 | 32.464809 |
| *Lampropeltis getula* | -91.70579 | 32.47374 |
| *Lampropeltis getula* | -82.249861 | 32.478782 |
| *Lampropeltis getula* | -111.063094 | 32.489116 |
| *Lampropeltis getula* | -108.57 | 32.49 |
| *Lampropeltis getula* | -103.16 | 32.49 |
| *Lampropeltis getula* | -110.89497 | 32.49028 |
| *Lampropeltis getula* | -81.493047 | 32.495056 |
| *Lampropeltis getula* | -106.93865 | 32.49825 |
| *Lampropeltis getula* | -81.0347 | 32.5018 |
| *Lampropeltis getula* | -85.620712 | 32.502998 |
| *Lampropeltis getula* | -91.99049 | 32.50348 |
| *Lampropeltis getula* | -92.11943 | 32.50996 |
| *Lampropeltis getula* | -110.88586 | 32.52391 |
| *Lampropeltis getula* | -92.036644 | 32.525715 |
| *Lampropeltis getula* | -92.63883 | 32.53155 |
| *Lampropeltis getula* | -92.24516 | 32.5337 |
| *Lampropeltis getula* | -106.9903 | 32.53405 |
| *Lampropeltis getula* | -117.11917 | 32.53528 |
| *Lampropeltis getula* | -85.894668 | 32.544076 |
| *Lampropeltis getula* | -92.92405 | 32.55129 |
| *Lampropeltis getula* | -92.02992 | 32.55354 |
| *Lampropeltis getula* | -93.3957 | 32.56176 |
| *Lampropeltis getula* | -117.08421 | 32.57592 |
| *Lampropeltis getula* | -109.95318 | 32.58228 |
| *Lampropeltis getula* | -80.34748 | 32.58738 |
| *Lampropeltis getula* | -109.8545 | 32.58826 |
| *Lampropeltis getula* | -110.770542 | 32.589083 |
| *Lampropeltis getula* | -92.08433 | 32.58971 |
| *Lampropeltis getula* | -109.88992 | 32.60003 |
| *Lampropeltis getula* | -110.835408 | 32.60843 |
| *Lampropeltis getula* | -117.06755 | 32.61038 |
| *Lampropeltis getula* | -107.28 | 32.62 |
| *Lampropeltis getula* | -107.03656 | 32.62581 |
| *Lampropeltis getula* | -116.9292 | 32.63775 |
| *Lampropeltis getula* | -112.35065 | 32.64046 |
| *Lampropeltis getula* | -116.78142 | 32.64425 |
| *Lampropeltis getula* | -116.85664 | 32.65354 |
| *Lampropeltis getula* | -117.06738 | 32.65466 |
| *Lampropeltis getula* | -117.03003 | 32.65784 |
| *Lampropeltis getula* | -116.09225 | 32.65955 |
| *Lampropeltis getula* | -107.14316 | 32.66045 |
| *Lampropeltis getula* | -116.81509 | 32.66405 |
| *Lampropeltis getula* | -114.75869 | 32.66483 |
| *Lampropeltis getula* | -115.68535 | 32.679167 |
| *Lampropeltis getula* | -115.60416 | 32.67959 |
| *Lampropeltis getula* | -117.05989 | 32.68342 |
| *Lampropeltis getula* | -109.00097 | 32.68655 |
| *Lampropeltis getula* | -117.24615 | 32.68741 |
| *Lampropeltis getula* | -91.30042 | 32.69051 |
| *Lampropeltis getula* | -91.87109 | 32.69136 |
| *Lampropeltis getula* | -114.59852 | 32.69827 |
| *Lampropeltis getula* | -116.49656 | 32.71375 |
| *Lampropeltis getula* | -116.76159 | 32.71517 |
| *Lampropeltis getula* | -117.15639 | 32.71528 |
| *Lampropeltis getula* | -114.62438 | 32.72535 |
| *Lampropeltis getula* | -116.9551 | 32.72809 |
| *Lampropeltis getula* | -117.03419 | 32.72871 |
| *Lampropeltis getula* | -117.00344 | 32.73164 |
| *Lampropeltis getula* | -109.71626 | 32.73693 |
| *Lampropeltis getula* | -117.06503 | 32.73977 |
| *Lampropeltis getula* | -117.24621 | 32.74352 |
| *Lampropeltis getula* | -114.70801 | 32.74492 |
| *Lampropeltis getula* | -117.21481 | 32.74635 |
| *Lampropeltis getula* | -117.0294 | 32.75442 |
| *Lampropeltis getula* | -97.797 | 32.759 |
| *Lampropeltis getula* | -79.952 | 32.764 |
| *Lampropeltis getula* | -116.99007 | 32.77143 |
| *Lampropeltis getula* | -117.14046 | 32.77333 |
| *Lampropeltis getula* | -82.2377 | 32.7824 |
| *Lampropeltis getula* | -117.17267 | 32.78435 |
| *Lampropeltis getula* | -117.11057 | 32.78496 |
| *Lampropeltis getula* | -115.73348 | 32.79107 |
| *Lampropeltis getula* | -117.07869 | 32.79283 |
| *Lampropeltis getula* | -117.18926 | 32.79621 |
| *Lampropeltis getula* | -116.9328 | 32.79958 |
| *Lampropeltis getula* | -96.75412 | 32.80082 |
| *Lampropeltis getula* | -117.24816 | 32.80403 |
| *Lampropeltis getula* | -116.98941 | 32.80515 |
| *Lampropeltis getula* | -79.97715 | 32.807877 |
| *Lampropeltis getula* | -116.8634 | 32.81116 |
| *Lampropeltis getula* | -109.61252 | 32.8128 |
| *Lampropeltis getula* | -117.21131 | 32.81478 |
| *Lampropeltis getula* | -117.05343 | 32.82636 |
| *Lampropeltis getula* | -116.16551 | 32.82675 |
| *Lampropeltis getula* | -117.00728 | 32.83243 |
| *Lampropeltis getula* | -117.26861 | 32.83257 |
| *Lampropeltis getula* | -114.55611 | 32.8328 |
| *Lampropeltis getula* | -116.73224 | 32.83291 |
| *Lampropeltis getula* | -86.568669 | 32.834473 |
| *Lampropeltis getula* | -83.65577 | 32.83712 |
| *Lampropeltis getula* | -116.68346 | 32.83843 |
| *Lampropeltis getula* | -116.7995 | 32.84327 |
| *Lampropeltis getula* | -116.64883 | 32.84928 |
| *Lampropeltis getula* | -87.66666 | 32.85 |
| *Lampropeltis getula* | -116.91268 | 32.85838 |
| *Lampropeltis getula* | -117.252 | 32.859 |
| *Lampropeltis getula* | -117.19995 | 32.86268 |
| *Lampropeltis getula* | -116.86062 | 32.86395 |
| *Lampropeltis getula* | -116.87859 | 32.86444 |
| *Lampropeltis getula* | -81.109539 | 32.870965 |
| *Lampropeltis getula* | -117.15001 | 32.87304 |
| *Lampropeltis getula* | -116.44724 | 32.88727 |
| *Lampropeltis getula* | -117.14124 | 32.88999 |
| *Lampropeltis getula* | -109.486 | 32.89559 |
| *Lampropeltis getula* | -85.539592 | 32.895647 |
| *Lampropeltis getula* | -109.837474 | 32.895979 |
| *Lampropeltis getula* | -105.9 | 32.9 |
| *Lampropeltis getula* | -117.09753 | 32.90894 |
| *Lampropeltis getula* | -116.87846 | 32.92131 |
| *Lampropeltis getula* | -116.9866 | 32.9269 |
| *Lampropeltis getula* | -117.12917 | 32.94111 |
| *Lampropeltis getula* | -117.26333 | 32.94923 |
| *Lampropeltis getula* | -116.58 | 32.95 |
| *Lampropeltis getula* | -117.23859 | 32.9509 |
| *Lampropeltis getula* | -101.22663 | 32.95491 |
| *Lampropeltis getula* | -107.49 | 32.96 |
| *Lampropeltis getula* | -117.035 | 32.962778 |
| *Lampropeltis getula* | -116.83195 | 32.96905 |
| *Lampropeltis getula* | -117.23529 | 32.97426 |
| *Lampropeltis getula* | -99.18635 | 32.9768 |
| *Lampropeltis getula* | -86.88704 | 32.983461 |
| *Lampropeltis getula* | -87.29369 | 32.98382 |
| *Lampropeltis getula* | -81.369225 | 32.989448 |
| *Lampropeltis getula* | -116.83159 | 32.98953 |
| *Lampropeltis getula* | -115.05297 | 32.99128 |
| *Lampropeltis getula* | -116.5695 | 32.99342 |
| *Lampropeltis getula* | -117.11546 | 32.99692 |
| *Lampropeltis getula* | -87.63 | 32.9975 |
| *Lampropeltis getula* | -84.0888 | 32.9997 |
| *Lampropeltis getula* | -108.6 | 33 |
| *Lampropeltis getula* | -104 | 33 |
| *Lampropeltis getula* | -112.66637 | 33.00067 |
| *Lampropeltis getula* | -116.97069 | 33.00867 |
| *Lampropeltis getula* | -116.45318 | 33.01326 |
| *Lampropeltis getula* | -89.585004 | 33.014672 |
| *Lampropeltis getula* | -116.91405 | 33.01717 |
| *Lampropeltis getula* | -80.35254 | 33.01911 |
| *Lampropeltis getula* | -116.89568 | 33.02343 |
| *Lampropeltis getula* | -104.36 | 33.03 |
| *Lampropeltis getula* | -117.12941 | 33.031 |
| *Lampropeltis getula* | -112.3593 | 33.032456 |
| *Lampropeltis getula* | -112.3593 | 33.03246 |
| *Lampropeltis getula* | -117.11292 | 33.0334 |
| *Lampropeltis getula* | -116.82971 | 33.03654 |
| *Lampropeltis getula* | -117.15971 | 33.03917 |
| *Lampropeltis getula* | -117.1973 | 33.0421 |
| *Lampropeltis getula* | -117.25858 | 33.04308 |
| *Lampropeltis getula* | -116.8708 | 33.0451 |
| *Lampropeltis getula* | -117.29698 | 33.0481 |
| *Lampropeltis getula* | -107.28 | 33.05 |
| *Lampropeltis getula* | -117.06648 | 33.05187 |
| *Lampropeltis getula* | -112.047617 | 33.05501 |
| *Lampropeltis getula* | -117.08651 | 33.06131 |
| *Lampropeltis getula* | -116.81229 | 33.06322 |
| *Lampropeltis getula* | -89.55403 | 33.06392 |
| *Lampropeltis getula* | -116.74717 | 33.06962 |
| *Lampropeltis getula* | -96.577622 | 33.069671 |
| *Lampropeltis getula* | -116.54057 | 33.07223 |
| *Lampropeltis getula* | -116.90131 | 33.07226 |
| *Lampropeltis getula* | -117.11925 | 33.07284 |
| *Lampropeltis getula* | -116.80174 | 33.07494 |
| *Lampropeltis getula* | -116.60113 | 33.07862 |
| *Lampropeltis getula* | -117.03211 | 33.08272 |
| *Lampropeltis getula* | -116.50895 | 33.08332 |
| *Lampropeltis getula* | -116.57384 | 33.0857 |
| *Lampropeltis getula* | -117.26619 | 33.09117 |
| *Lampropeltis getula* | -116.6358 | 33.09508 |
| *Lampropeltis getula* | -116.6826 | 33.1067 |
| *Lampropeltis getula* | -116.73985 | 33.10825 |
| *Lampropeltis getula* | -116.448534 | 33.109423 |
| *Lampropeltis getula* | -117.21817 | 33.12797 |
| *Lampropeltis getula* | -116.36483 | 33.13399 |
| *Lampropeltis getula* | -116.29847 | 33.13531 |
| *Lampropeltis getula* | -116.85069 | 33.1398 |
| *Lampropeltis getula* | -116.53548 | 33.14083 |
| *Lampropeltis getula* | -116.15327 | 33.14953 |
| *Lampropeltis getula* | -87.56917 | 33.15189 |
| *Lampropeltis getula* | -116.18317 | 33.15319 |
| *Lampropeltis getula* | -117.349722 | 33.158056 |
| *Lampropeltis getula* | -117.03106 | 33.15911 |
| *Lampropeltis getula* | -116.55733 | 33.16295 |
| *Lampropeltis getula* | -93.299292 | 33.17543 |
| *Lampropeltis getula* | -87.45167 | 33.18944 |
| *Lampropeltis getula* | -116.70603 | 33.19218 |
| *Lampropeltis getula* | -117.26294 | 33.19425 |
| *Lampropeltis getula* | -117.173501 | 33.1958 |
| *Lampropeltis getula* | -87.48835 | 33.20103 |
| *Lampropeltis getula* | -117.24264 | 33.20515 |
| *Lampropeltis getula* | -85.580478 | 33.205637 |
| *Lampropeltis getula* | -116.4268 | 33.20691 |
| *Lampropeltis getula* | -87.56917 | 33.20972 |
| *Lampropeltis getula* | -117.0342 | 33.21837 |
| *Lampropeltis getula* | -117.0342 | 33.218373 |
| *Lampropeltis getula* | -97.396244 | 33.224071 |
| *Lampropeltis getula* | -116.33882 | 33.22687 |
| *Lampropeltis getula* | -117.394 | 33.227 |
| *Lampropeltis getula* | -87.59451 | 33.22889 |
| *Lampropeltis getula* | -116.686017 | 33.24255 |
| *Lampropeltis getula* | -83.922138 | 33.247446 |
| *Lampropeltis getula* | -117.413 | 33.249 |
| *Lampropeltis getula* | -81.7477 | 33.2497 |
| *Lampropeltis getula* | -116.37508 | 33.25652 |
| *Lampropeltis getula* | -107.26 | 33.27 |
| *Lampropeltis getula* | -117.23612 | 33.27089 |
| *Lampropeltis getula* | -117.37265 | 33.2769 |
| *Lampropeltis getula* | -116.635567 | 33.2775 |
| *Lampropeltis getula* | -116.63622 | 33.27757 |
| *Lampropeltis getula* | -94.439328 | 33.285078 |
| *Lampropeltis getula* | -87.68423 | 33.28537 |
| *Lampropeltis getula* | -116.39751 | 33.2873 |
| *Lampropeltis getula* | -116.95754 | 33.28811 |
| *Lampropeltis getula* | -81.64889 | 33.29251 |
| *Lampropeltis getula* | -116.68755 | 33.293883 |
| *Lampropeltis getula* | -117.44465 | 33.31136 |
| *Lampropeltis getula* | -85.655678 | 33.312464 |
| *Lampropeltis getula* | -116.35024 | 33.31971 |
| *Lampropeltis getula* | -117.23629 | 33.32797 |
| *Lampropeltis getula* | -116.95541 | 33.33486 |
| *Lampropeltis getula* | -118.327103 | 33.342822 |
| *Lampropeltis getula* | -117.337029 | 33.354351 |
| *Lampropeltis getula* | -117.22673 | 33.35594 |
| *Lampropeltis getula* | -117.1068 | 33.35979 |
| *Lampropeltis getula* | -103.78 | 33.36 |
| *Lampropeltis getula* | -117.41576 | 33.36136 |
| *Lampropeltis getula* | -117.371964 | 33.3638 |
| *Lampropeltis getula* | -117.07469 | 33.37045 |
| *Lampropeltis getula* | -117.25032 | 33.37643 |
| *Lampropeltis getula* | -116.77236 | 33.38433 |
| *Lampropeltis getula* | -117.49216 | 33.38443 |
| *Lampropeltis getula* | -81.614452 | 33.385517 |
| *Lampropeltis getula* | -111.95652 | 33.385586 |
| *Lampropeltis getula* | -111.95652 | 33.38559 |
| *Lampropeltis getula* | -117.263 | 33.3975 |
| *Lampropeltis getula* | -103.7 | 33.4 |
| *Lampropeltis getula* | -84.47072 | 33.40768 |
| *Lampropeltis getula* | -115.18692 | 33.43068 |
| *Lampropeltis getula* | -83.7333 | 33.4666 |
| *Lampropeltis getula* | -117.4797 | 33.46833 |
| *Lampropeltis getula* | -106.06 | 33.48 |
| *Lampropeltis getula* | -85.947995 | 33.482475 |
| *Lampropeltis getula* | -82.314269 | 33.491841 |
| *Lampropeltis getula* | -112.9364 | 33.4936 |
| *Lampropeltis getula* | -117.66261 | 33.50173 |
| *Lampropeltis getula* | -116.06477 | 33.5079 |
| *Lampropeltis getula* | -117.5522 | 33.52707 |
| *Lampropeltis getula* | -116.09334 | 33.53985 |
| *Lampropeltis getula* | -117.781 | 33.545 |
| *Lampropeltis getula* | -83.136371 | 33.5577 |
| *Lampropeltis getula* | -85.1171 | 33.5666 |
| *Lampropeltis getula* | -117.81445 | 33.56666 |
| *Lampropeltis getula* | -116.11253 | 33.56932 |
| *Lampropeltis getula* | -116.02358 | 33.5697 |
| *Lampropeltis getula* | -117.06571 | 33.57954 |
| *Lampropeltis getula* | -86.49083 | 33.59083 |
| *Lampropeltis getula* | -117.67063 | 33.59645 |
| *Lampropeltis getula* | -117.87 | 33.603 |
| *Lampropeltis getula* | -114.66623 | 33.60666 |
| *Lampropeltis getula* | -117.72343 | 33.61111 |
| *Lampropeltis getula* | -111.6821 | 33.6127 |
| *Lampropeltis getula* | -85.83532 | 33.61412 |
| *Lampropeltis getula* | -117.08907 | 33.61795 |
| *Lampropeltis getula* | -110.9217 | 33.61939 |
| *Lampropeltis getula* | -96.413 | 33.622 |
| *Lampropeltis getula* | -117.84667 | 33.63701 |
| *Lampropeltis getula* | -96.60817 | 33.64008 |
| *Lampropeltis getula* | -85.83167 | 33.65972 |
| *Lampropeltis getula* | -86.561235 | 33.661207 |
| *Lampropeltis getula* | -98.845397 | 33.664325 |
| *Lampropeltis getula* | -117.59492 | 33.66668 |
| *Lampropeltis getula* | -104.4 | 33.67 |
| *Lampropeltis getula* | -96.90567 | 33.67489 |
| *Lampropeltis getula* | -117.01158 | 33.67528 |
| *Lampropeltis getula* | -117.08373 | 33.6807 |
| *Lampropeltis getula* | -84.79 | 33.6861 |
| *Lampropeltis getula* | -117.992 | 33.687 |
| *Lampropeltis getula* | -104.4 | 33.7 |
| *Lampropeltis getula* | -117.35088 | 33.70168 |
| *Lampropeltis getula* | -117.02242 | 33.70748 |
| *Lampropeltis getula* | -118.023 | 33.711 |
| *Lampropeltis getula* | -116.21557 | 33.72059 |
| *Lampropeltis getula* | -111.84634 | 33.73567 |
| *Lampropeltis getula* | -118.33056 | 33.73656 |
| *Lampropeltis getula* | -117.66247 | 33.73957 |
| *Lampropeltis getula* | -118.31027 | 33.74386 |
| *Lampropeltis getula* | -118.387958 | 33.745446 |
| *Lampropeltis getula* | -116.97201 | 33.74752 |
| *Lampropeltis getula* | -94.6239 | 33.74776 |
| *Lampropeltis getula* | -84.5813 | 33.7493 |
| *Lampropeltis getula* | -84.6176 | 33.751 |
| *Lampropeltis getula* | -116.20464 | 33.75103 |
| *Lampropeltis getula* | -117.76753 | 33.76766 |
| *Lampropeltis getula* | -117.7301 | 33.76793 |
| *Lampropeltis getula* | -118.37778 | 33.77083 |
| *Lampropeltis getula* | -118.35931 | 33.77106 |
| *Lampropeltis getula* | -118.32587 | 33.77316 |
| *Lampropeltis getula* | -118.32587 | 33.773161 |
| *Lampropeltis getula* | -118.11409 | 33.77648 |
| *Lampropeltis getula* | -118.16648 | 33.77702 |
| *Lampropeltis getula* | -118.212 | 33.7847 |
| *Lampropeltis getula* | -115.82487 | 33.79332 |
| *Lampropeltis getula* | -118.18916 | 33.80433 |
| *Lampropeltis getula* | -117.25188 | 33.80452 |
| *Lampropeltis getula* | -116.675 | 33.810278 |
| *Lampropeltis getula* | -118.08731 | 33.81289 |
| *Lampropeltis getula* | -106.89 | 33.82 |
| *Lampropeltis getula* | -117.74191 | 33.82528 |
| *Lampropeltis getula* | -106.88 | 33.83 |
| *Lampropeltis getula* | -116.54534 | 33.83032 |
| *Lampropeltis getula* | -111.9507 | 33.83334 |
| *Lampropeltis getula* | -117.09241 | 33.83504 |
| *Lampropeltis getula* | -117.43316 | 33.84094 |
| *Lampropeltis getula* | -117.46111 | 33.8425 |
| *Lampropeltis getula* | -103.2 | 33.85 |
| *Lampropeltis getula* | -117.3732 | 33.85349 |
| *Lampropeltis getula* | -96.50256 | 33.8571 |
| *Lampropeltis getula* | -116.30452 | 33.86487 |
| *Lampropeltis getula* | -116.30452 | 33.86487 |
| *Lampropeltis getula* | -117.68112 | 33.86962 |
| *Lampropeltis getula* | -96.81889 | 33.8835 |
| *Lampropeltis getula* | -98.514519 | 33.884359 |
| *Lampropeltis getula* | -116.7874 | 33.88868 |
| *Lampropeltis getula* | -95.49444 | 33.89481 |
| *Lampropeltis getula* | -116.67848 | 33.90085 |
| *Lampropeltis getula* | -117.92986 | 33.9051 |
| *Lampropeltis getula* | -112.675032 | 33.907927 |
| *Lampropeltis getula* | -117.90012 | 33.91668 |
| *Lampropeltis getula* | -118.28003 | 33.91846 |
| *Lampropeltis getula* | -78.0206 | 33.9214 |
| *Lampropeltis getula* | -116.70347 | 33.9226 |
| *Lampropeltis getula* | -116.53919 | 33.9248 |
| *Lampropeltis getula* | -116.49402 | 33.92485 |
| *Lampropeltis getula* | -116.59913 | 33.92807 |
| *Lampropeltis getula* | -116.82757 | 33.93038 |
| *Lampropeltis getula* | -116.64176 | 33.9379 |
| *Lampropeltis getula* | -106.87 | 33.94 |
| *Lampropeltis getula* | -94.91682 | 33.94181 |
| *Lampropeltis getula* | -94.75897 | 33.94336 |
| *Lampropeltis getula* | -117.07234 | 33.94343 |
| *Lampropeltis getula* | -104.64 | 33.95 |
| *Lampropeltis getula* | -118.0006 | 33.95298 |
| *Lampropeltis getula* | -96.84238 | 33.9549 |
| *Lampropeltis getula* | -118.35375 | 33.95678 |
| *Lampropeltis getula* | -117.93752 | 33.9591 |
| *Lampropeltis getula* | -117.76766 | 33.95952 |
| *Lampropeltis getula* | -96.28484 | 33.95994 |
| *Lampropeltis getula* | -118.00951 | 33.96364 |
| *Lampropeltis getula* | -118.44141 | 33.96742 |
| *Lampropeltis getula* | -117.86692 | 33.96763 |
| *Lampropeltis getula* | -117.43033 | 33.96918 |
| *Lampropeltis getula* | -117.74906 | 33.96922 |
| *Lampropeltis getula* | -83.743694 | 33.969271 |
| *Lampropeltis getula* | -94.81792 | 33.97051 |
| *Lampropeltis getula* | -116.54502 | 33.97453 |
| *Lampropeltis getula* | -116.65303 | 33.9788 |
| *Lampropeltis getula* | -118.0321 | 33.99571 |
| *Lampropeltis getula* | -118.43074 | 34.00595 |
| *Lampropeltis getula* | -93.426658 | 34.006226 |
| *Lampropeltis getula* | -116.02027 | 34.00857 |
| *Lampropeltis getula* | -118.28546 | 34.01608 |
| *Lampropeltis getula* | -95.26722 | 34.01972 |
| *Lampropeltis getula* | -114.48111 | 34.02177 |
| *Lampropeltis getula* | -117.02405 | 34.02597 |
| *Lampropeltis getula* | -95.34193 | 34.02791 |
| *Lampropeltis getula* | -117.94732 | 34.02892 |
| *Lampropeltis getula* | -102.8288 | 34.02956 |
| *Lampropeltis getula* | -118.37699 | 34.03508 |
| *Lampropeltis getula* | -116.30901 | 34.0378 |
| *Lampropeltis getula* | -118.758 | 34.04 |
| *Lampropeltis getula* | -106.86799 | 34.04629 |
| *Lampropeltis getula* | -118.18162 | 34.05151 |
| *Lampropeltis getula* | -117.18707 | 34.05212 |
| *Lampropeltis getula* | -117.81971 | 34.05873 |
| *Lampropeltis getula* | -117.65 | 34.06333 |
| *Lampropeltis getula* | -112.84494 | 34.06644 |
| *Lampropeltis getula* | -78.317 | 34.067 |
| *Lampropeltis getula* | -98.672179 | 34.068031 |
| *Lampropeltis getula* | -118.58597 | 34.06815 |
| *Lampropeltis getula* | -118.44211 | 34.07105 |
| *Lampropeltis getula* | -116.22442 | 34.07672 |
| *Lampropeltis getula* | -117.80388 | 34.08613 |
| *Lampropeltis getula* | -118.31958 | 34.08924 |
| *Lampropeltis getula* | -103.04 | 34.09 |
| *Lampropeltis getula* | -118.12537 | 34.09399 |
| *Lampropeltis getula* | -116.36954 | 34.09425 |
| *Lampropeltis getula* | -118.2202 | 34.10148 |
| *Lampropeltis getula* | -118.411268 | 34.102841 |
| *Lampropeltis getula* | -118.84765 | 34.10413 |
| *Lampropeltis getula* | -118.72111 | 34.105 |
| *Lampropeltis getula* | -116.14889 | 34.10833 |
| *Lampropeltis getula* | -116.11128 | 34.1194 |
| *Lampropeltis getula* | -117.29325 | 34.12135 |
| *Lampropeltis getula* | -119.09118 | 34.1253 |
| *Lampropeltis getula* | -94.80732 | 34.12761 |
| *Lampropeltis getula* | -116.27731 | 34.13472 |
| *Lampropeltis getula* | -116.123389 | 34.135137 |
| *Lampropeltis getula* | -116.05348 | 34.13564 |
| *Lampropeltis getula* | -118.84856 | 34.14273 |
| *Lampropeltis getula* | -84.83 | 34.15 |
| *Lampropeltis getula* | -117.92372 | 34.15626 |
| *Lampropeltis getula* | -117.45452 | 34.16606 |
| *Lampropeltis getula* | -117.8126 | 34.17151 |
| *Lampropeltis getula* | -86.91694 | 34.18917 |
| *Lampropeltis getula* | -118.65153 | 34.20004 |
| *Lampropeltis getula* | -113.5148 | 34.20759 |
| *Lampropeltis getula* | -77.9447 | 34.2257 |
| *Lampropeltis getula* | -95.94256 | 34.2322 |
| *Lampropeltis getula* | -99.0734 | 34.24914 |
| *Lampropeltis getula* | -119.07074 | 34.253 |
| *Lampropeltis getula* | -114.13502 | 34.26727 |
| *Lampropeltis getula* | -89.64393 | 34.27191 |
| *Lampropeltis getula* | -114.15568 | 34.29577 |
| *Lampropeltis getula* | -87.33611 | 34.30694 |
| *Lampropeltis getula* | -94.68892 | 34.31971 |
| *Lampropeltis getula* | -117.42741 | 34.32586 |
| *Lampropeltis getula* | -101.662 | 34.33 |
| *Lampropeltis getula* | -89.51564 | 34.33309 |
| *Lampropeltis getula* | -117.23028 | 34.34333 |
| *Lampropeltis getula* | -106.88 | 34.35 |
| *Lampropeltis getula* | -96.71078 | 34.35349 |
| *Lampropeltis getula* | -117.42491 | 34.37088 |
| *Lampropeltis getula* | -106.86 | 34.38 |
| *Lampropeltis getula* | -119.46278 | 34.3975 |
| *Lampropeltis getula* | -93.8308 | 34.3975 |
| *Lampropeltis getula* | -89.77171 | 34.40015 |
| *Lampropeltis getula* | -119.30944 | 34.40472 |
| *Lampropeltis getula* | -103.44 | 34.41 |
| *Lampropeltis getula* | -117.80929 | 34.414206 |
| *Lampropeltis getula* | -116.89132 | 34.41857 |
| *Lampropeltis getula* | -106.83 | 34.42 |
| *Lampropeltis getula* | -106.73 | 34.42 |
| *Lampropeltis getula* | -117.31926 | 34.42407 |
| *Lampropeltis getula* | -118.53943 | 34.42431 |
| *Lampropeltis getula* | -119.73374 | 34.42612 |
| *Lampropeltis getula* | -97.14719 | 34.42619 |
| *Lampropeltis getula* | -119.697 | 34.427 |
| *Lampropeltis getula* | -89.39143 | 34.42751 |
| *Lampropeltis getula* | -106.41 | 34.43 |
| *Lampropeltis getula* | -112.07016 | 34.43127 |
| *Lampropeltis getula* | -119.66159 | 34.43529 |
| *Lampropeltis getula* | -97.1106 | 34.43884 |
| *Lampropeltis getula* | -119.83222 | 34.43944 |
| *Lampropeltis getula* | -96.07154 | 34.44379 |
| *Lampropeltis getula* | -119.879 | 34.445 |
| *Lampropeltis getula* | -119.71 | 34.44861 |
| *Lampropeltis getula* | -106.82 | 34.45 |
| *Lampropeltis getula* | -119.74307 | 34.45422 |
| *Lampropeltis getula* | -120.41361 | 34.46028 |
| *Lampropeltis getula* | -95.98857 | 34.46144 |
| *Lampropeltis getula* | -97.15216 | 34.4622 |
| *Lampropeltis getula* | -96.04059 | 34.46524 |
| *Lampropeltis getula* | -119.80028 | 34.4675 |
| *Lampropeltis getula* | -89.761577 | 34.468774 |
| *Lampropeltis getula* | -119.77417 | 34.46944 |
| *Lampropeltis getula* | -120.055 | 34.474 |
| *Lampropeltis getula* | -120.202 | 34.476 |
| *Lampropeltis getula* | -117.25871 | 34.47876 |
| *Lampropeltis getula* | -119.50528 | 34.48944 |
| *Lampropeltis getula* | -106.76 | 34.49 |
| *Lampropeltis getula* | -120.22593 | 34.49347 |
| *Lampropeltis getula* | -119.79111 | 34.495 |
| *Lampropeltis getula* | -106.8 | 34.5 |
| *Lampropeltis getula* | -117.185 | 34.50083 |
| *Lampropeltis getula* | -89.50291 | 34.50185 |
| *Lampropeltis getula* | -119.61 | 34.515 |
| *Lampropeltis getula* | -119.79178 | 34.51639 |
| *Lampropeltis getula* | -120.478 | 34.52 |
| *Lampropeltis getula* | -95.35229 | 34.52206 |
| *Lampropeltis getula* | -117.2872 | 34.52863 |
| *Lampropeltis getula* | -119.62667 | 34.53306 |
| *Lampropeltis getula* | -119.8625 | 34.53556 |
| *Lampropeltis getula* | -96.21947 | 34.53589 |
| *Lampropeltis getula* | -94.88545 | 34.53784 |
| *Lampropeltis getula* | -93.02833 | 34.54222 |
| *Lampropeltis getula* | -118.10956 | 34.54539 |
| *Lampropeltis getula* | -119.77378 | 34.54619 |
| *Lampropeltis getula* | -119.89582 | 34.54762 |
| *Lampropeltis getula* | -95.94463 | 34.55032 |
| *Lampropeltis getula* | -117.40263 | 34.55377 |
| *Lampropeltis getula* | -112.45278 | 34.55472 |
| *Lampropeltis getula* | -120.08257 | 34.55829 |
| *Lampropeltis getula* | -106.79 | 34.56 |
| *Lampropeltis getula* | -120.4075 | 34.56361 |
| *Lampropeltis getula* | -94.23908 | 34.57475 |
| *Lampropeltis getula* | -120.00889 | 34.58639 |
| *Lampropeltis getula* | -94.26482 | 34.58804 |
| *Lampropeltis getula* | -90.630164 | 34.597275 |
| *Lampropeltis getula* | -106.8 | 34.6 |
| *Lampropeltis getula* | -117.53095 | 34.60178 |
| *Lampropeltis getula* | -116.97834 | 34.60406 |
| *Lampropeltis getula* | -120.11514 | 34.60482 |
| *Lampropeltis getula* | -120.05222 | 34.61028 |
| *Lampropeltis getula* | -119.37073 | 34.61467 |
| *Lampropeltis getula* | -120.37722 | 34.61472 |
| *Lampropeltis getula* | -120.08944 | 34.61611 |
| *Lampropeltis getula* | -84.904 | 34.6163 |
| *Lampropeltis getula* | -118.45058 | 34.61968 |
| *Lampropeltis getula* | -79.93645 | 34.6211 |
| *Lampropeltis getula* | -95.38711 | 34.62645 |
| *Lampropeltis getula* | -112.42996 | 34.63406 |
| *Lampropeltis getula* | -78.4978 | 34.6427 |
| *Lampropeltis getula* | -94.70366 | 34.64864 |
| *Lampropeltis getula* | -112.2908 | 34.65031 |
| *Lampropeltis getula* | -117.82642 | 34.65385 |
| *Lampropeltis getula* | -76.57 | 34.667 |
| *Lampropeltis getula* | -120.22546 | 34.66954 |
| *Lampropeltis getula* | -120.45191 | 34.67132 |
| *Lampropeltis getula* | -98.38573 | 34.67337 |
| *Lampropeltis getula* | -94.26528 | 34.67972 |
| *Lampropeltis getula* | -94.61111 | 34.68 |
| *Lampropeltis getula* | -94.35201 | 34.68281 |
| *Lampropeltis getula* | -94.55815 | 34.68782 |
| *Lampropeltis getula* | -94.13639 | 34.69 |
| *Lampropeltis getula* | -119.90707 | 34.69001 |
| *Lampropeltis getula* | -117.78217 | 34.6954 |
| *Lampropeltis getula* | -113.906595 | 34.696177 |
| *Lampropeltis getula* | -118.13583 | 34.69806 |
| *Lampropeltis getula* | -94.46222 | 34.70778 |
| *Lampropeltis getula* | -98.44774 | 34.71107 |
| *Lampropeltis getula* | -94.67917 | 34.7125 |
| *Lampropeltis getula* | -111.9215 | 34.7178 |
| *Lampropeltis getula* | -117.45971 | 34.72116 |
| *Lampropeltis getula* | -87.7922 | 34.7294 |
| *Lampropeltis getula* | -86.581307 | 34.730885 |
| *Lampropeltis getula* | -112 | 34.73333 |
| *Lampropeltis getula* | -120.57527 | 34.74179 |
| *Lampropeltis getula* | -115.65635 | 34.74411 |
| *Lampropeltis getula* | -119.40935 | 34.74826 |
| *Lampropeltis getula* | -98.682064 | 34.750961 |
| *Lampropeltis getula* | -79.88327 | 34.75563 |
| *Lampropeltis getula* | -117.4751 | 34.76383 |
| *Lampropeltis getula* | -84.143528 | 34.766204 |
| *Lampropeltis getula* | -118.49678 | 34.77449 |
| *Lampropeltis getula* | -115.64719 | 34.77567 |
| *Lampropeltis getula* | -115.1755 | 34.776433 |
| *Lampropeltis getula* | -118.11234 | 34.78336 |
| *Lampropeltis getula* | -106.97 | 34.79 |
| *Lampropeltis getula* | -112.0625 | 34.79664 |
| *Lampropeltis getula* | -107 | 34.8 |
| *Lampropeltis getula* | -120.19125 | 34.80023 |
| *Lampropeltis getula* | -119.47573 | 34.84309 |
| *Lampropeltis getula* | -117.50564 | 34.86598 |
| *Lampropeltis getula* | -98.86845 | 34.86696 |
| *Lampropeltis getula* | -106.72 | 34.87 |
| *Lampropeltis getula* | -120.35944 | 34.87056 |
| *Lampropeltis getula* | -120.455556 | 34.870833 |
| *Lampropeltis getula* | -106.69 | 34.88 |
| *Lampropeltis getula* | -79.3825 | 34.8829 |
| *Lampropeltis getula* | -79.3825 | 34.8829 |
| *Lampropeltis getula* | -118.903179 | 34.884036 |
| *Lampropeltis getula* | -91.31155 | 34.88406 |
| *Lampropeltis getula* | -117.039555 | 34.898609 |
| *Lampropeltis getula* | -119.716721 | 34.903904 |
| *Lampropeltis getula* | -99.30646 | 34.90546 |
| *Lampropeltis getula* | -106.688289 | 34.907421 |
| *Lampropeltis getula* | -117.51731 | 34.9228 |
| *Lampropeltis getula* | -101.6619 | 34.9333 |
| *Lampropeltis getula* | -105.08717 | 34.93794 |
| *Lampropeltis getula* | -115.44113 | 34.93896 |
| *Lampropeltis getula* | -107.14 | 34.94 |
| *Lampropeltis getula* | -115.61614 | 34.94104 |
| *Lampropeltis getula* | -117.52896 | 34.95005 |
| *Lampropeltis getula* | -95.87786 | 34.95967 |
| *Lampropeltis getula* | -117.83363 | 34.96063 |
| *Lampropeltis getula* | -119.72533 | 34.96504 |
| *Lampropeltis getula* | -107.25 | 34.97 |
| *Lampropeltis getula* | -83.38488 | 34.97243 |
| *Lampropeltis getula* | -106.66 | 34.98 |
| *Lampropeltis getula* | -88.067 | 34.982 |
| *Lampropeltis getula* | -119.781042 | 34.984225 |
| *Lampropeltis getula* | -119.79776 | 34.98875 |
| *Lampropeltis getula* | -95.35519 | 34.99223 |
| *Lampropeltis getula* | -117.54056 | 34.99233 |
| *Lampropeltis getula* | -117.64925 | 35.00193 |
| *Lampropeltis getula* | -118.94884 | 35.00482 |
| *Lampropeltis getula* | -120.18658 | 35.02986 |
| *Lampropeltis getula* | -114.63589 | 35.03124 |
| *Lampropeltis getula* | -95.48137 | 35.03386 |
| *Lampropeltis getula* | -92.920254 | 35.035744 |
| *Lampropeltis getula* | -76.08824 | 35.03794 |
| *Lampropeltis getula* | -118.30275 | 35.039813 |
| *Lampropeltis getula* | -119.886733 | 35.04055 |
| *Lampropeltis getula* | -110.76695 | 35.04759 |
| *Lampropeltis getula* | -118.199804 | 35.051168 |
| *Lampropeltis getula* | -119.91495 | 35.0544 |
| *Lampropeltis getula* | -111.161261 | 35.065581 |
| *Lampropeltis getula* | -76.0638 | 35.0696 |
| *Lampropeltis getula* | -118.345883 | 35.070183 |
| *Lampropeltis getula* | -118.299919 | 35.097448 |
| *Lampropeltis getula* | -120.11629 | 35.09861 |
| *Lampropeltis getula* | -98.43723 | 35.09867 |
| *Lampropeltis getula* | -119.080056 | 35.12546 |
| *Lampropeltis getula* | -118.13153 | 35.12546 |
| *Lampropeltis getula* | -97.343 | 35.13117 |
| *Lampropeltis getula* | -118.435175 | 35.138992 |
| *Lampropeltis getula* | -97.41166 | 35.139 |
| *Lampropeltis getula* | -94.66982 | 35.16905 |
| *Lampropeltis getula* | -103.69 | 35.17 |
| *Lampropeltis getula* | -114.74508 | 35.17324 |
| *Lampropeltis getula* | -103.7 | 35.18 |
| *Lampropeltis getula* | -114.816934 | 35.184087 |
| *Lampropeltis getula* | -102.206444 | 35.190968 |
| *Lampropeltis getula* | -117.984325 | 35.198219 |
| *Lampropeltis getula* | -97.44571 | 35.20987 |
| *Lampropeltis getula* | -96.9499 | 35.21677 |
| *Lampropeltis getula* | -104.25 | 35.22 |
| *Lampropeltis getula* | -90.723883 | 35.22253 |
| *Lampropeltis getula* | -75.648 | 35.226 |
| *Lampropeltis getula* | -75.624902 | 35.235112 |
| *Lampropeltis getula* | -75.57369 | 35.23598 |
| *Lampropeltis getula* | -91.69724 | 35.242783 |
| *Lampropeltis getula* | -75.597 | 35.245 |
| *Lampropeltis getula* | -101.841202 | 35.246158 |
| *Lampropeltis getula* | -94.13017 | 35.25179 |
| *Lampropeltis getula* | -94.130907 | 35.254395 |
| *Lampropeltis getula* | -75.568 | 35.256 |
| *Lampropeltis getula* | -75.5354 | 35.2607 |
| *Lampropeltis getula* | -118.66898 | 35.2642 |
| *Lampropeltis getula* | -119.302308 | 35.2676 |
| *Lampropeltis getula* | -103.52 | 35.27 |
| *Lampropeltis getula* | -118.685232 | 35.270171 |
| *Lampropeltis getula* | -115.27472 | 35.27167 |
| *Lampropeltis getula* | -118.62633 | 35.2719 |
| *Lampropeltis getula* | -119.297896 | 35.283648 |
| *Lampropeltis getula* | -77.225139 | 35.285481 |
| *Lampropeltis getula* | -118.004577 | 35.296155 |
| *Lampropeltis getula* | -110.41887 | 35.299549 |
| *Lampropeltis getula* | -118.11653 | 35.304559 |
| *Lampropeltis getula* | -114.73084 | 35.31142 |
| *Lampropeltis getula* | -118.044755 | 35.315411 |
| *Lampropeltis getula* | -116.40702 | 35.32379 |
| *Lampropeltis getula* | -80.7189 | 35.32988 |
| *Lampropeltis getula* | -120.686461 | 35.334833 |
| *Lampropeltis getula* | -119.91181 | 35.34118 |
| *Lampropeltis getula* | -75.504 | 35.35 |
| *Lampropeltis getula* | -104.21275 | 35.3514 |
| *Lampropeltis getula* | -120.692887 | 35.351859 |
| *Lampropeltis getula* | -120.27618 | 35.35732 |
| *Lampropeltis getula* | -104.19 | 35.36 |
| *Lampropeltis getula* | -117.65108 | 35.36889 |
| *Lampropeltis getula* | -97.35361 | 35.37727 |
| *Lampropeltis getula* | -117.59864 | 35.38434 |
| *Lampropeltis getula* | -120.15083 | 35.3875 |
| *Lampropeltis getula* | -119.27306 | 35.39377 |
| *Lampropeltis getula* | -103.9 | 35.4 |
| *Lampropeltis getula* | -90.789601 | 35.401976 |
| *Lampropeltis getula* | -120.620277 | 35.40361 |
| *Lampropeltis getula* | -117.57651 | 35.4061 |
| *Lampropeltis getula* | -117.66879 | 35.41966 |
| *Lampropeltis getula* | -118.792486 | 35.441746 |
| *Lampropeltis getula* | -120.398173 | 35.45509 |
| *Lampropeltis getula* | -115.6331 | 35.45814 |
| *Lampropeltis getula* | -118.719801 | 35.475047 |
| *Lampropeltis getula* | -103.27 | 35.48 |
| *Lampropeltis getula* | -119.30473 | 35.500058 |
| *Lampropeltis getula* | -119.272572 | 35.5033 |
| *Lampropeltis getula* | -120.994 | 35.504 |
| *Lampropeltis getula* | -78.15875 | 35.507797 |
| *Lampropeltis getula* | -118.51915 | 35.514 |
| *Lampropeltis getula* | -118.20347 | 35.51615 |
| *Lampropeltis getula* | -116.82109 | 35.54221 |
| *Lampropeltis getula* | -117.66969 | 35.55221 |
| *Lampropeltis getula* | -120.66389 | 35.59389 |
| *Lampropeltis getula* | -119.65028 | 35.60056 |
| *Lampropeltis getula* | -120.32493 | 35.60531 |
| *Lampropeltis getula* | -117.52762 | 35.60643 |
| *Lampropeltis getula* | -76.2157 | 35.6363 |
| *Lampropeltis getula* | -118.936017 | 35.65118 |
| *Lampropeltis getula* | -76.20696 | 35.66132 |
| *Lampropeltis getula* | -91.612021 | 35.665756 |
| *Lampropeltis getula* | -94.83863 | 35.676533 |
| *Lampropeltis getula* | -91.705639 | 35.707625 |
| *Lampropeltis getula* | -118.430756 | 35.726055 |
| *Lampropeltis getula* | -83.47028 | 35.72611 |
| *Lampropeltis getula* | -78.76421 | 35.72642 |
| *Lampropeltis getula* | -115.93274 | 35.7403 |
| *Lampropeltis getula* | -76.10735 | 35.745 |
| *Lampropeltis getula* | -114.75 | 35.75 |
| *Lampropeltis getula* | -94.159404 | 35.770989 |
| *Lampropeltis getula* | -78.63882 | 35.7801 |
| *Lampropeltis getula* | -110.1 | 35.8 |
| *Lampropeltis getula* | -98.96965 | 35.80584 |
| *Lampropeltis getula* | -101.31571 | 35.83087 |
| *Lampropeltis getula* | -76.421322 | 35.848775 |
| *Lampropeltis getula* | -118.4467 | 35.86188 |
| *Lampropeltis getula* | -121.212558 | 35.864089 |
| *Lampropeltis getula* | -120.86766 | 35.87162 |
| *Lampropeltis getula* | -99.69897 | 35.87679 |
| *Lampropeltis getula* | -90.098393 | 35.877626 |
| *Lampropeltis getula* | -75.9627 | 35.8913 |
| *Lampropeltis getula* | -99.71842 | 35.89695 |
| *Lampropeltis getula* | -92.633125 | 35.908483 |
| *Lampropeltis getula* | -90.763822 | 35.934508 |
| *Lampropeltis getula* | -85.024326 | 35.949949 |
| *Lampropeltis getula* | -121.09953 | 35.96626 |
| *Lampropeltis getula* | -83.944898 | 35.982929 |
| *Lampropeltis getula* | -116.27009 | 35.98298 |
| *Lampropeltis getula* | -94.18691 | 35.99447 |
| *Lampropeltis getula* | -120.47203 | 36.00247 |
| *Lampropeltis getula* | -121.17417 | 36.00389 |
| *Lampropeltis getula* | -97.21878 | 36.01451 |
| *Lampropeltis getula* | -95.72127 | 36.02325 |
| *Lampropeltis getula* | -94.235295 | 36.029522 |
| *Lampropeltis getula* | -115.36 | 36.03 |
| *Lampropeltis getula* | -112.160904 | 36.039645 |
| *Lampropeltis getula* | -96.86537 | 36.04872 |
| *Lampropeltis getula* | -115.016 | 36.066 |
| *Lampropeltis getula* | -97.05105 | 36.07035 |
| *Lampropeltis getula* | -120.147 | 36.07217 |
| *Lampropeltis getula* | -117.51315 | 36.07366 |
| *Lampropeltis getula* | -95.99551 | 36.0756 |
| *Lampropeltis getula* | -95.56321 | 36.08615 |
| *Lampropeltis getula* | -120.461533 | 36.098206 |
| *Lampropeltis getula* | -95.88338 | 36.12791 |
| *Lampropeltis getula* | -120.743667 | 36.131678 |
| *Lampropeltis getula* | -94.78443 | 36.13998 |
| *Lampropeltis getula* | -96.2238 | 36.1402 |
| *Lampropeltis getula* | -115.884401 | 36.144259 |
| *Lampropeltis getula* | -85.501871 | 36.164599 |
| *Lampropeltis getula* | -91.23333 | 36.16667 |
| *Lampropeltis getula* | -97.30063 | 36.17381 |
| *Lampropeltis getula* | -95.30364 | 36.18079 |
| *Lampropeltis getula* | -120.776 | 36.19291 |
| *Lampropeltis getula* | -91.174515 | 36.203691 |
| *Lampropeltis getula* | -94.77938 | 36.20512 |
| *Lampropeltis getula* | -100.65 | 36.22778 |
| *Lampropeltis getula* | -114.266 | 36.233 |
| *Lampropeltis getula* | -120.31439 | 36.2463 |
| *Lampropeltis getula* | -119.28639 | 36.25778 |
| *Lampropeltis getula* | -121.083756 | 36.259886 |
| *Lampropeltis getula* | -121.08376 | 36.25989 |
| *Lampropeltis getula* | -121.30215 | 36.28083 |
| *Lampropeltis getula* | -118.848567 | 36.287025 |
| *Lampropeltis getula* | -115.416114 | 36.298356 |
| *Lampropeltis getula* | -117.47824 | 36.33591 |
| *Lampropeltis getula* | -121.02572 | 36.33825 |
| *Lampropeltis getula* | -121.50342 | 36.3401 |
| *Lampropeltis getula* | -118.76026 | 36.3497 |
| *Lampropeltis getula* | -89.424858 | 36.351788 |
| *Lampropeltis getula* | -97.22242 | 36.36214 |
| *Lampropeltis getula* | -121.55969 | 36.37191 |
| *Lampropeltis getula* | -117.279225 | 36.400377 |
| *Lampropeltis getula* | -115.5651 | 36.40748 |
| *Lampropeltis getula* | -119.45759 | 36.40773 |
| *Lampropeltis getula* | -118.9257 | 36.41337 |
| *Lampropeltis getula* | -76.3 | 36.417 |
| *Lampropeltis getula* | -115.38555 | 36.42029 |
| *Lampropeltis getula* | -118.908 | 36.43297 |
| *Lampropeltis getula* | -121.185119 | 36.447056 |
| *Lampropeltis getula* | -115.3666 | 36.45 |
| *Lampropeltis getula* | -121.04935 | 36.4503 |
| *Lampropeltis getula* | -114.38366 | 36.46934 |
| *Lampropeltis getula* | -121.154164 | 36.477341 |
| *Lampropeltis getula* | -121.731819 | 36.479311 |
| *Lampropeltis getula* | -121.17435 | 36.496451 |
| *Lampropeltis getula* | -121.08355 | 36.4972 |
| *Lampropeltis getula* | -103.111706 | 36.502349 |
| *Lampropeltis getula* | -114.45 | 36.516 |
| *Lampropeltis getula* | -120.096842 | 36.534736 |
| *Lampropeltis getula* | -89.260372 | 36.539567 |
| *Lampropeltis getula* | -76.45988 | 36.54715 |
| *Lampropeltis getula* | -121.70691 | 36.55275 |
| *Lampropeltis getula* | -121.56311 | 36.55275 |
| *Lampropeltis getula* | -114.44735 | 36.55716 |
| *Lampropeltis getula* | -121.895854 | 36.558915 |
| *Lampropeltis getula* | -121.176367 | 36.560383 |
| *Lampropeltis getula* | -115.828247 | 36.568916 |
| *Lampropeltis getula* | -77.53 | 36.57 |
| *Lampropeltis getula* | -93.001134 | 36.581701 |
| *Lampropeltis getula* | -120.990375 | 36.5844 |
| *Lampropeltis getula* | -115.99582 | 36.59272 |
| *Lampropeltis getula* | -120.75745 | 36.602883 |
| *Lampropeltis getula* | -76.21 | 36.61 |
| *Lampropeltis getula* | -76.0589 | 36.61552 |
| *Lampropeltis getula* | -76.09 | 36.64 |
| *Lampropeltis getula* | -76.09 | 36.64 |
| *Lampropeltis getula* | -116.43 | 36.65 |
| *Lampropeltis getula* | -84.348463 | 36.650584 |
| *Lampropeltis getula* | -116 | 36.66 |
| *Lampropeltis getula* | -76.12 | 36.66 |
| *Lampropeltis getula* | -92.499165 | 36.667534 |
| *Lampropeltis getula* | -93.862629 | 36.67643 |
| *Lampropeltis getula* | -82.33626 | 36.6771 |
| *Lampropeltis getula* | -116.229936 | 36.683208 |
| *Lampropeltis getula* | -117.90028 | 36.68778 |
| *Lampropeltis getula* | -121.2251 | 36.693783 |
| *Lampropeltis getula* | -99.36723 | 36.69476 |
| *Lampropeltis getula* | -82.64641 | 36.70709 |
| *Lampropeltis getula* | -76.03524 | 36.72061 |
| *Lampropeltis getula* | -116.07218 | 36.72096 |
| *Lampropeltis getula* | -84.161462 | 36.72468 |
| *Lampropeltis getula* | -121.27749 | 36.72905 |
| *Lampropeltis getula* | -76.38 | 36.73 |
| *Lampropeltis getula* | -114.21667 | 36.73333 |
| *Lampropeltis getula* | -75.947 | 36.734 |
| *Lampropeltis getula* | -76.09803 | 36.73997 |
| *Lampropeltis getula* | -76.54284 | 36.74445 |
| *Lampropeltis getula* | -115.99582 | 36.74645 |
| *Lampropeltis getula* | -99.36672 | 36.7542 |
| *Lampropeltis getula* | -120.8573 | 36.7589 |
| *Lampropeltis getula* | -119.77182 | 36.76615 |
| *Lampropeltis getula* | -121.306461 | 36.771764 |
| *Lampropeltis getula* | -77.0991 | 36.7727 |
| *Lampropeltis getula* | -77.10024 | 36.77349 |
| *Lampropeltis getula* | -88.015067 | 36.784151 |
| *Lampropeltis getula* | -76.0391 | 36.78447 |
| *Lampropeltis getula* | -82.5598 | 36.78663 |
| *Lampropeltis getula* | -116.16128 | 36.79274 |
| *Lampropeltis getula* | -118.30699 | 36.80246 |
| *Lampropeltis getula* | -76.02934 | 36.81618 |
| *Lampropeltis getula* | -119.08681 | 36.82367 |
| *Lampropeltis getula* | -100.51254 | 36.84474 |
| *Lampropeltis getula* | -78.59218 | 36.85369 |
| *Lampropeltis getula* | -120.455 | 36.8589 |
| *Lampropeltis getula* | -121.40117 | 36.85998 |
| *Lampropeltis getula* | -112.73761 | 36.861896 |
| *Lampropeltis getula* | -75.98487 | 36.8708 |
| *Lampropeltis getula* | -121.570522 | 36.871607 |
| *Lampropeltis getula* | -102.8211 | 36.8901 |
| *Lampropeltis getula* | -89.089984 | 36.89115 |
| *Lampropeltis getula* | -96.793657 | 36.891809 |
| *Lampropeltis getula* | -76.25854 | 36.89807 |
| *Lampropeltis getula* | -120.6885 | 36.9182 |
| *Lampropeltis getula* | -120.634506 | 36.924495 |
| *Lampropeltis getula* | -120.63451 | 36.9245 |
| *Lampropeltis getula* | -121.829 | 36.927 |
| *Lampropeltis getula* | -121.468714 | 36.928271 |
| *Lampropeltis getula* | -119.24439 | 36.93333 |
| *Lampropeltis getula* | -108.97 | 36.94 |
| *Lampropeltis getula* | -121.557969 | 36.940097 |
| *Lampropeltis getula* | -96.550339 | 36.949982 |
| *Lampropeltis getula* | -76.993 | 36.9523 |
| *Lampropeltis getula* | -89.043357 | 36.963342 |
| *Lampropeltis getula* | -122.015778 | 36.973751 |
| *Lampropeltis getula* | -77.73 | 36.98 |
| *Lampropeltis getula* | -121.7174 | 36.98507 |
| *Lampropeltis getula* | -120.63047 | 36.98789 |
| *Lampropeltis getula* | -78.8 | 36.99 |
| *Lampropeltis getula* | -120.707967 | 36.992583 |
| *Lampropeltis getula* | -120.616332 | 36.997124 |
| *Lampropeltis getula* | -89.17872 | 37.00875 |
| *Lampropeltis getula* | -91.001037 | 37.015344 |
| *Lampropeltis getula* | -120.099055 | 37.01781 |
| *Lampropeltis getula* | -99.299446 | 37.032501 |
| *Lampropeltis getula* | -97.60778 | 37.033611 |
| *Lampropeltis getula* | -120.63462 | 37.034466 |
| *Lampropeltis getula* | -78.43459 | 37.04278 |
| *Lampropeltis getula* | -96.054726 | 37.064445 |
| *Lampropeltis getula* | -119.721443 | 37.090636 |
| *Lampropeltis getula* | -120.77992 | 37.09505 |
| *Lampropeltis getula* | -121.01656 | 37.09778 |
| *Lampropeltis getula* | -78.3191 | 37.0979 |
| *Lampropeltis getula* | -96.79556 | 37.10278 |
| *Lampropeltis getula* | -113.57667 | 37.105 |
| *Lampropeltis getula* | -117.213607 | 37.113798 |
| *Lampropeltis getula* | -121.93888 | 37.11991 |
| *Lampropeltis getula* | -96.208054 | 37.121113 |
| *Lampropeltis getula* | -101.79 | 37.124167 |
| *Lampropeltis getula* | -76.563 | 37.135 |
| *Lampropeltis getula* | -75.875 | 37.144 |
| *Lampropeltis getula* | -122.079951 | 37.152712 |
| *Lampropeltis getula* | -100.43 | 37.162498 |
| *Lampropeltis getula* | -118.293125 | 37.166218 |
| *Lampropeltis getula* | -120.829761 | 37.166483 |
| *Lampropeltis getula* | -95.698608 | 37.167221 |
| *Lampropeltis getula* | -122.005 | 37.183283 |
| *Lampropeltis getula* | -121.973153 | 37.184788 |
| *Lampropeltis getula* | -122.032767 | 37.185317 |
| *Lampropeltis getula* | -100.34417 | 37.189724 |
| *Lampropeltis getula* | -112.90778 | 37.20417 |
| *Lampropeltis getula* | -121.89562 | 37.20552 |
| *Lampropeltis getula* | -98.69945 | 37.21833 |
| *Lampropeltis getula* | -121.973611 | 37.226667 |
| *Lampropeltis getula* | -91.410487 | 37.231971 |
| *Lampropeltis getula* | -121.755867 | 37.232119 |
| *Lampropeltis getula* | -99.997498 | 37.238609 |
| *Lampropeltis getula* | -118.177431 | 37.247548 |
| *Lampropeltis getula* | -121.788775 | 37.265189 |
| *Lampropeltis getula* | -121.736294 | 37.268306 |
| *Lampropeltis getula* | -121.837683 | 37.27965 |
| *Lampropeltis getula* | -76.93 | 37.28 |
| *Lampropeltis getula* | -76.6421 | 37.2832 |
| *Lampropeltis getula* | -76.74005 | 37.29108 |
| *Lampropeltis getula* | -121.86499 | 37.29481 |
| *Lampropeltis getula* | -109.86893 | 37.29728 |
| *Lampropeltis getula* | -79.28 | 37.3 |
| *Lampropeltis getula* | -78.3919 | 37.3021 |
| *Lampropeltis getula* | -121.950491 | 37.32027 |
| *Lampropeltis getula* | -118.07892 | 37.32052 |
| *Lampropeltis getula* | -77.973904 | 37.321007 |
| *Lampropeltis getula* | -109.03442 | 37.32573 |
| *Lampropeltis getula* | -113.7246 | 37.3259 |
| *Lampropeltis getula* | -75.91997 | 37.333 |
| *Lampropeltis getula* | -118.033376 | 37.335311 |
| *Lampropeltis getula* | -113.69357 | 37.33808 |
| *Lampropeltis getula* | -121.716057 | 37.342239 |
| *Lampropeltis getula* | -95.122162 | 37.354778 |
| *Lampropeltis getula* | -121.954572 | 37.356733 |
| *Lampropeltis getula* | -77.95 | 37.36 |
| *Lampropeltis getula* | -121.481369 | 37.360017 |
| *Lampropeltis getula* | -118.34877 | 37.36143 |
| *Lampropeltis getula* | -117.725087 | 37.361434 |
| *Lampropeltis getula* | -115.158732 | 37.364184 |
| *Lampropeltis getula* | -122.246796 | 37.365721 |
| *Lampropeltis getula* | -113.7362 | 37.37305 |
| *Lampropeltis getula* | -121.774675 | 37.378108 |
| *Lampropeltis getula* | -122.113058 | 37.385267 |
| *Lampropeltis getula* | -97.086917 | 37.389431 |
| *Lampropeltis getula* | -122.216939 | 37.391939 |
| *Lampropeltis getula* | -121.44417 | 37.39514 |
| *Lampropeltis getula* | -120.876961 | 37.396487 |
| *Lampropeltis getula* | -118.378557 | 37.397711 |
| *Lampropeltis getula* | -118.27148 | 37.40699 |
| *Lampropeltis getula* | -76.52 | 37.41 |
| *Lampropeltis getula* | -121.37137 | 37.42055 |
| *Lampropeltis getula* | -76.9011 | 37.4238 |
| *Lampropeltis getula* | -122.23017 | 37.42667 |
| *Lampropeltis getula* | -122.169169 | 37.427219 |
| *Lampropeltis getula* | -99.05272 | 37.42853 |
| *Lampropeltis getula* | -121.51148 | 37.4351 |
| *Lampropeltis getula* | -121.319161 | 37.438247 |
| *Lampropeltis getula* | -122.141944 | 37.441944 |
| *Lampropeltis getula* | -98.774048 | 37.442001 |
| *Lampropeltis getula* | -121.265067 | 37.453603 |
| *Lampropeltis getula* | -76.8402 | 37.4546 |
| *Lampropeltis getula* | -115.192811 | 37.465086 |
| *Lampropeltis getula* | -79.51 | 37.47 |
| *Lampropeltis getula* | -78.857995 | 37.494972 |
| *Lampropeltis getula* | -122.34645 | 37.51895 |
| *Lampropeltis getula* | -121.82059 | 37.52057 |
| *Lampropeltis getula* | -120.335479 | 37.522205 |
| *Lampropeltis getula* | -78.48 | 37.53 |
| *Lampropeltis getula* | -121.854233 | 37.533233 |
| *Lampropeltis getula* | -75.73 | 37.55 |
| *Lampropeltis getula* | -96.0252 | 37.5519 |
| *Lampropeltis getula* | -101.336945 | 37.565556 |
| *Lampropeltis getula* | -89.317054 | 37.569769 |
| *Lampropeltis getula* | -121.68937 | 37.57171 |
| *Lampropeltis getula* | -121.587775 | 37.573744 |
| *Lampropeltis getula* | -121.79572 | 37.58775 |
| *Lampropeltis getula* | -121.63045 | 37.59528 |
| *Lampropeltis getula* | -121.86547 | 37.59547 |
| *Lampropeltis getula* | -77.4 | 37.6 |
| *Lampropeltis getula* | -121.70293 | 37.62268 |
| *Lampropeltis getula* | -77.68 | 37.63 |
| *Lampropeltis getula* | -121.5134 | 37.630217 |
| *Lampropeltis getula* | -94.703331 | 37.635555 |
| *Lampropeltis getula* | -94.82304 | 37.63975 |
| *Lampropeltis getula* | -121.595767 | 37.641467 |
| *Lampropeltis getula* | -121.6245 | 37.646267 |
| *Lampropeltis getula* | -121.650117 | 37.6471 |
| *Lampropeltis getula* | -121.54152 | 37.65089 |
| *Lampropeltis getula* | -121.48495 | 37.65626 |
| *Lampropeltis getula* | -121.73178 | 37.66147 |
| *Lampropeltis getula* | -121.694327 | 37.665277 |
| *Lampropeltis getula* | -121.7498 | 37.66693 |
| *Lampropeltis getula* | -121.62889 | 37.68694 |
| *Lampropeltis getula* | -121.590597 | 37.693747 |
| *Lampropeltis getula* | -121.695805 | 37.694003 |
| *Lampropeltis getula* | -95.452499 | 37.711201 |
| *Lampropeltis getula* | -121.549133 | 37.713433 |
| *Lampropeltis getula* | -97.407776 | 37.716946 |
| *Lampropeltis getula* | -118.436461 | 37.725621 |
| *Lampropeltis getula* | -121.86779 | 37.72665 |
| *Lampropeltis getula* | -118.05361 | 37.73081 |
| *Lampropeltis getula* | -75.64303 | 37.74045 |
| *Lampropeltis getula* | -121.53167 | 37.74167 |
| *Lampropeltis getula* | -121.61783 | 37.7445 |
| *Lampropeltis getula* | -121.5892 | 37.744683 |
| *Lampropeltis getula* | -122.44612 | 37.7568 |
| *Lampropeltis getula* | -122.445183 | 37.75853 |
| *Lampropeltis getula* | -120.82928 | 37.773056 |
| *Lampropeltis getula* | -122.1231 | 37.7846 |
| *Lampropeltis getula* | -86.489296 | 37.790393 |
| *Lampropeltis getula* | -95.09111 | 37.79083 |
| *Lampropeltis getula* | -95.843887 | 37.796665 |
| *Lampropeltis getula* | -121.74897 | 37.8003 |
| *Lampropeltis getula* | -78.49617 | 37.80367 |
| *Lampropeltis getula* | -95.43528 | 37.80416 |
| *Lampropeltis getula* | -122.269722 | 37.804444 |
| *Lampropeltis getula* | -118.49415 | 37.82009 |
| *Lampropeltis getula* | -120.152281 | 37.821225 |
| *Lampropeltis getula* | -118.00528 | 37.82389 |
| *Lampropeltis getula* | -122.127884 | 37.830536 |
| *Lampropeltis getula* | -122.00468 | 37.8375 |
| *Lampropeltis getula* | -87.79889 | 37.84028 |
| *Lampropeltis getula* | -120.615028 | 37.845178 |
| *Lampropeltis getula* | -122.2695 | 37.85442 |
| *Lampropeltis getula* | -99.857224 | 37.855 |
| *Lampropeltis getula* | -122.1936 | 37.861 |
| *Lampropeltis getula* | -121.927262 | 37.861 |
| *Lampropeltis getula* | -93.618672 | 37.861907 |
| *Lampropeltis getula* | -78.05239 | 37.87101 |
| *Lampropeltis getula* | -122.243528 | 37.87488 |
| *Lampropeltis getula* | -78.07 | 37.88 |
| *Lampropeltis getula* | -122.11396 | 37.88555 |
| *Lampropeltis getula* | -122.247468 | 37.900292 |
| *Lampropeltis getula* | -122.19151 | 37.90063 |
| *Lampropeltis getula* | -122.061861 | 37.900993 |
| *Lampropeltis getula* | -121.879251 | 37.909577 |
| *Lampropeltis getula* | -121.87925 | 37.90958 |
| *Lampropeltis getula* | -122.282627 | 37.929196 |
| *Lampropeltis getula* | -122.015556 | 37.936944 |
| *Lampropeltis getula* | -122.140371 | 37.938132 |
| *Lampropeltis getula* | -121.370133 | 37.944889 |
| *Lampropeltis getula* | -120.91164 | 37.94489 |
| *Lampropeltis getula* | -96.852776 | 37.954445 |
| *Lampropeltis getula* | -122.291511 | 37.954638 |
| *Lampropeltis getula* | -122.560556 | 37.974722 |
| *Lampropeltis getula* | -96.16333 | 37.98056 |
| *Lampropeltis getula* | -122.114017 | 37.994317 |
| *Lampropeltis getula* | -120.38584 | 38.00599 |
| *Lampropeltis getula* | -122.5411 | 38.01373 |
| *Lampropeltis getula* | -121.95147 | 38.0162 |
| *Lampropeltis getula* | -101.9915 | 38.03557 |
| *Lampropeltis getula* | -122.187 | 38.037 |
| *Lampropeltis getula* | -96.725281 | 38.042778 |
| *Lampropeltis getula* | -97.423332 | 38.043056 |
| *Lampropeltis getula* | -122.873 | 38.059 |
| *Lampropeltis getula* | -122.80305 | 38.070908 |
| *Lampropeltis getula* | -120.55782 | 38.07816 |
| *Lampropeltis getula* | -78.78 | 38.1 |
| *Lampropeltis getula* | -103.527209 | 38.129689 |
| *Lampropeltis getula* | -122.113503 | 38.156731 |
| *Lampropeltis getula* | -120.610502 | 38.157688 |
| *Lampropeltis getula* | -121.705822 | 38.168844 |
| *Lampropeltis getula* | -120.829187 | 38.178989 |
| *Lampropeltis getula* | -83.455238 | 38.180246 |
| *Lampropeltis getula* | -120.615377 | 38.190358 |
| *Lampropeltis getula* | -122.597927 | 38.197012 |
| *Lampropeltis getula* | -122.645131 | 38.208342 |
| *Lampropeltis getula* | -120.70347 | 38.229067 |
| *Lampropeltis getula* | -95.248056 | 38.233889 |
| *Lampropeltis getula* | -122.034 | 38.26548 |
| *Lampropeltis getula* | -122.43735 | 38.278917 |
| *Lampropeltis getula* | -120.70689 | 38.285486 |
| *Lampropeltis getula* | -121.441 | 38.285667 |
| *Lampropeltis getula* | -122.371387 | 38.287343 |
| *Lampropeltis getula* | -121.264167 | 38.291667 |
| *Lampropeltis getula* | -122.30055 | 38.30433 |
| *Lampropeltis getula* | -121.44425 | 38.3155 |
| *Lampropeltis getula* | -122.353733 | 38.330432 |
| *Lampropeltis getula* | -93.304873 | 38.334689 |
| *Lampropeltis getula* | -121.908889 | 38.348333 |
| *Lampropeltis getula* | -95.248611 | 38.34861 |
| *Lampropeltis getula* | -120.775818 | 38.412899 |
| *Lampropeltis getula* | -95.478699 | 38.426601 |
| *Lampropeltis getula* | -93.972433 | 38.434766 |
| *Lampropeltis getula* | -82.13308 | 38.43683 |
| *Lampropeltis getula* | -118.64 | 38.44 |
| *Lampropeltis getula* | -122.71333 | 38.44056 |
| *Lampropeltis getula* | -99.830559 | 38.450554 |
| *Lampropeltis getula* | -101.670502 | 38.457901 |
| *Lampropeltis getula* | -120.7165 | 38.47311 |
| *Lampropeltis getula* | -98.636391 | 38.478054 |
| *Lampropeltis getula* | -122.135 | 38.47965 |
| *Lampropeltis getula* | -116.095807 | 38.480547 |
| *Lampropeltis getula* | -100.356392 | 38.480835 |
| *Lampropeltis getula* | -122.44487 | 38.48469 |
| *Lampropeltis getula* | -122.489969 | 38.488548 |
| *Lampropeltis getula* | -122.59036 | 38.48978 |
| *Lampropeltis getula* | -99.701302 | 38.501301 |
| *Lampropeltis getula* | -121.254551 | 38.513014 |
| *Lampropeltis getula* | -122.027192 | 38.523827 |
| *Lampropeltis getula* | -121.877333 | 38.53023 |
| *Lampropeltis getula* | -121.187067 | 38.555617 |
| *Lampropeltis getula* | -121.263611 | 38.580278 |
| *Lampropeltis getula* | -122.57975 | 38.58216 |
| *Lampropeltis getula* | -118.710083 | 38.583972 |
| *Lampropeltis getula* | -98.045097 | 38.602699 |
| *Lampropeltis getula* | -123.0081 | 38.60547 |
| *Lampropeltis getula* | -123.183217 | 38.622053 |
| *Lampropeltis getula* | -121.221617 | 38.634383 |
| *Lampropeltis getula* | -121.388458 | 38.635304 |
| *Lampropeltis getula* | -121.114617 | 38.643433 |
| *Lampropeltis getula* | -121.044142 | 38.661809 |
| *Lampropeltis getula* | -97.998055 | 38.667221 |
| *Lampropeltis getula* | -121.681211 | 38.684181 |
| *Lampropeltis getula* | -121.371097 | 38.685825 |
| *Lampropeltis getula* | -123.0234 | 38.693277 |
| *Lampropeltis getula* | -118.634442 | 38.699636 |
| *Lampropeltis getula* | -121.46669 | 38.71771 |
| *Lampropeltis getula* | -122.50402 | 38.7217 |
| *Lampropeltis getula* | -92.398645 | 38.73388 |
| *Lampropeltis getula* | -97.93745 | 38.73806 |
| *Lampropeltis getula* | -121.291457 | 38.746 |
| *Lampropeltis getula* | -100.232597 | 38.762501 |
| *Lampropeltis getula* | -121.310847 | 38.763883 |
| *Lampropeltis getula* | -83.171303 | 38.772778 |
| *Lampropeltis getula* | -123.012094 | 38.784808 |
| *Lampropeltis getula* | -101.195297 | 38.7915 |
| *Lampropeltis getula* | -121.874658 | 38.793844 |
| *Lampropeltis getula* | -120.86064 | 38.79631 |
| *Lampropeltis getula* | -97.719719 | 38.815861 |
| *Lampropeltis getula* | -96.177498 | 38.8475 |
| *Lampropeltis getula* | -96.9188 | 38.8496 |
| *Lampropeltis getula* | -78.585 | 38.854 |
| *Lampropeltis getula* | -121.803 | 38.86219 |
| *Lampropeltis getula* | -101.6211 | 38.8787 |
| *Lampropeltis getula* | -77.3 | 38.88 |
| *Lampropeltis getula* | -122.27183 | 38.88247 |
| *Lampropeltis getula* | -123.054342 | 38.883642 |
| *Lampropeltis getula* | -91.948852 | 38.897038 |
| *Lampropeltis getula* | -123.156374 | 38.899477 |
| *Lampropeltis getula* | -122.562911 | 38.899508 |
| *Lampropeltis getula* | -95.3255 | 38.9057 |
| *Lampropeltis getula* | -101.0842 | 38.9077 |
| *Lampropeltis getula* | -122.264178 | 38.908608 |
| *Lampropeltis getula* | -95.53717 | 38.91441 |
| *Lampropeltis getula* | -122.31391 | 38.91661 |
| *Lampropeltis getula* | -83.42625 | 38.92242 |
| *Lampropeltis getula* | -83.426247 | 38.922424 |
| *Lampropeltis getula* | -96.508057 | 38.927776 |
| *Lampropeltis getula* | -74.905819 | 38.934847 |
| *Lampropeltis getula* | -99.563752 | 38.938521 |
| *Lampropeltis getula* | -122.339 | 38.94037 |
| *Lampropeltis getula* | -123.33933 | 38.95975 |
| *Lampropeltis getula* | -122.339161 | 38.971819 |
| *Lampropeltis getula* | -95.37275 | 38.97306 |
| *Lampropeltis getula* | -118.266 | 38.983 |
| *Lampropeltis getula* | -96.177963 | 38.992779 |
| *Lampropeltis getula* | -96.519722 | 38.996113 |
| *Lampropeltis getula* | -95.32722 | 39.00056 |
| *Lampropeltis getula* | -120.991389 | 39.000833 |
| *Lampropeltis getula* | -123.08209 | 39.001158 |
| *Lampropeltis getula* | -99.0478 | 39.0094 |
| *Lampropeltis getula* | -122.00361 | 39.01322 |
| *Lampropeltis getula* | -122.37693 | 39.01483 |
| *Lampropeltis getula* | -96.547997 | 39.022099 |
| *Lampropeltis getula* | -123.27636 | 39.03037 |
| *Lampropeltis getula* | -122.41862 | 39.03944 |
| *Lampropeltis getula* | -96.49583 | 39.05305 |
| *Lampropeltis getula* | -94.83722 | 39.06361 |
| *Lampropeltis getula* | -96.79889 | 39.06389 |
| *Lampropeltis getula* | -74.8242 | 39.0825 |
| *Lampropeltis getula* | -96.576668 | 39.094166 |
| *Lampropeltis getula* | -121.39672 | 39.10097 |
| *Lampropeltis getula* | -122.324583 | 39.105017 |
| *Lampropeltis getula* | -122.271136 | 39.137287 |
| *Lampropeltis getula* | -122.614911 | 39.169967 |
| *Lampropeltis getula* | -99.732117 | 39.178219 |
| *Lampropeltis getula* | -96.961113 | 39.22028 |
| *Lampropeltis getula* | -121.60113 | 39.22651 |
| *Lampropeltis getula* | -76.620438 | 39.284706 |
| *Lampropeltis getula* | -98.191389 | 39.321111 |
| *Lampropeltis getula* | -121.339278 | 39.334361 |
| *Lampropeltis getula* | -122.39078 | 39.35321 |
| *Lampropeltis getula* | -96.31472 | 39.3675 |
| *Lampropeltis getula* | -75.6919 | 39.3805 |
| *Lampropeltis getula* | -95.952 | 39.3836 |
| *Lampropeltis getula* | -123.3038 | 39.41318 |
| *Lampropeltis getula* | -75.6418 | 39.4419 |
| *Lampropeltis getula* | -87.392119 | 39.445157 |
| *Lampropeltis getula* | -87.39274 | 39.46846 |
| *Lampropeltis getula* | -96.633408 | 39.482121 |
| *Lampropeltis getula* | -75.0261 | 39.4861 |
| *Lampropeltis getula* | -119.9732 | 39.513361 |
| *Lampropeltis getula* | -101.5494 | 39.5315 |
| *Lampropeltis getula* | -75.838252 | 39.535014 |
| *Lampropeltis getula* | -123.401836 | 39.562493 |
| *Lampropeltis getula* | -119.74 | 39.58 |
| *Lampropeltis getula* | -96.40778 | 39.58805 |
| *Lampropeltis getula* | -123.343833 | 39.628361 |
| *Lampropeltis getula* | -118.487982 | 39.654954 |
| *Lampropeltis getula* | -123.346528 | 39.664972 |
| *Lampropeltis getula* | -123.3565 | 39.703083 |
| *Lampropeltis getula* | -123.312436 | 39.712603 |
| *Lampropeltis getula* | -123.271083 | 39.713083 |
| *Lampropeltis getula* | -121.83 | 39.74 |
| *Lampropeltis getula* | -96.6661 | 39.7483 |
| *Lampropeltis getula* | -121.472762 | 39.762708 |
| *Lampropeltis getula* | -99.9361 | 39.7773 |
| *Lampropeltis getula* | -121.5625 | 39.78529 |
| *Lampropeltis getula* | -123.07983 | 39.80222 |
| *Lampropeltis getula* | -74.66687 | 39.8175 |
| *Lampropeltis getula* | -74.66687 | 39.8175 |
| *Lampropeltis getula* | -99.7513 | 39.821 |
| *Lampropeltis getula* | -123.089694 | 39.825639 |
| *Lampropeltis getula* | -123.180833 | 39.829472 |
| *Lampropeltis getula* | -98.056351 | 39.865559 |
| *Lampropeltis getula* | -74.6333 | 39.8667 |
| *Lampropeltis getula* | -98.47361 | 39.905556 |
| *Lampropeltis getula* | -97.50972 | 39.94361 |
| *Lampropeltis getula* | -74.32637 | 39.9536 |
| *Lampropeltis getula* | -123.790525 | 40.019146 |
| *Lampropeltis getula* | -74.2181 | 40.0978 |
| *Lampropeltis getula* | -122.18314 | 40.20172 |
| *Lampropeltis getula* | -74.679674 | 40.211509 |
| *Lampropeltis getula* | -122.274934 | 40.214778 |
| *Lampropeltis getula* | -122.11828 | 40.43989 |
| *Lampropeltis getula* | -121.99131 | 40.44168 |
| *Lampropeltis getula* | -122.61807 | 40.47515 |
| *Lampropeltis getula* | -122.61807 | 40.47515 |
| *Lampropeltis getula* | -121.98064 | 40.51084 |
| *Lampropeltis getula* | -122.942325 | 40.649295 |
| *Lampropeltis getula* | -122.942325 | 40.649295 |
| *Lampropeltis getula* | -122.92936 | 40.69344 |
| *Lampropeltis getula* | -119.3666 | 40.7166 |
| *Lampropeltis getula* | -123.36058 | 40.79398 |
| *Lampropeltis getula* | -122.039175 | 40.858711 |
| *Lampropeltis getula* | -122.10972 | 40.88752 |
| *Lampropeltis getula* | -123.58333 | 40.89028 |
| *Lampropeltis getula* | -122.1095 | 40.90862 |
| *Lampropeltis getula* | -123.852718 | 40.939658 |
| *Lampropeltis getula* | -123.62788 | 40.93966 |
| *Lampropeltis getula* | -122.49 | 41.73 |
| *Lampropeltis getula* | -123.16891 | 42.38626 |

**S1B Dataset**

Occurrence records of *Pantherophis guttatus* in North America and occurrence records from invaded areas.

| Specie | Longitude | Latitude |
| --- | --- | --- |
| *Pantherophis guttatus* | -69.053425 | 12.226057 |
| *Pantherophis guttatus* | -60.903668 | 14.540655 |
| *Pantherophis guttatus* | -61.796428 | 17.060816 |
| *Pantherophis guttatus* | -62.829377 | 17.89812 |
| *Pantherophis guttatus* | -63.050684 | 18.212595 |
| *Pantherophis guttatus* | -64.948132 | 18.353721 |
| *Pantherophis guttatus* | -64.633278 | 18.433469 |
| *Pantherophis guttatus* | -81.254592 | 19.313269 |
| *Pantherophis guttatus* | -81.7917 | 24.5542 |
| *Pantherophis guttatus* | -81.77767 | 24.55607 |
| *Pantherophis guttatus* | -81.748694 | 24.556172 |
| *Pantherophis guttatus* | -81.796622 | 24.556886 |
| *Pantherophis guttatus* | -81.739764 | 24.5666 |
| *Pantherophis guttatus* | -81.7406 | 24.5725 |
| *Pantherophis guttatus* | -78.019533 | 24.632658 |
| *Pantherophis guttatus* | -81.561 | 24.64217 |
| *Pantherophis guttatus* | -81.5117 | 24.6422 |
| *Pantherophis guttatus* | -81.562742 | 24.646811 |
| *Pantherophis guttatus* | -81.412511 | 24.6604 |
| *Pantherophis guttatus* | -81.41301 | 24.66073 |
| *Pantherophis guttatus* | -81.44448 | 24.66199 |
| *Pantherophis guttatus* | -81.445294 | 24.663331 |
| *Pantherophis guttatus* | -81.391033 | 24.669278 |
| *Pantherophis guttatus* | -81.34842 | 24.66946 |
| *Pantherophis guttatus* | -81.39089 | 24.66962 |
| *Pantherophis guttatus* | -81.44388 | 24.67319 |
| *Pantherophis guttatus* | -81.444522 | 24.682644 |
| *Pantherophis guttatus* | -81.406317 | 24.684231 |
| *Pantherophis guttatus* | -81.370581 | 24.689544 |
| *Pantherophis guttatus* | -81.09997 | 24.69029 |
| *Pantherophis guttatus* | -81.37003 | 24.69064 |
| *Pantherophis guttatus* | -81.35782 | 24.6916 |
| *Pantherophis guttatus* | -81.40781 | 24.6919 |
| *Pantherophis guttatus* | -81.101678 | 24.694727 |
| *Pantherophis guttatus* | -81.102442 | 24.696467 |
| *Pantherophis guttatus* | -81.436636 | 24.713914 |
| *Pantherophis guttatus* | -81.07563 | 24.71619 |
| *Pantherophis guttatus* | -81.43696 | 24.71812 |
| *Pantherophis guttatus* | -81.38766 | 24.72074 |
| *Pantherophis guttatus* | -81.311469 | 24.732919 |
| *Pantherophis guttatus* | -80.978003 | 24.748125 |
| *Pantherophis guttatus* | -80.953721 | 24.762782 |
| *Pantherophis guttatus* | -80.9486 | 24.76439 |
| *Pantherophis guttatus* | -80.846603 | 24.804006 |
| *Pantherophis guttatus* | -80.8365 | 24.8081 |
| *Pantherophis guttatus* | -80.739206 | 24.849698 |
| *Pantherophis guttatus* | -78.056821 | 25.018762 |
| *Pantherophis guttatus* | -77.355474 | 25.047017 |
| *Pantherophis guttatus* | -80.41574 | 25.11487 |
| *Pantherophis guttatus* | -80.3664 | 25.18 |
| *Pantherophis guttatus* | -80.34256 | 25.20792 |
| *Pantherophis guttatus* | -80.324564 | 25.250394 |
| *Pantherophis guttatus* | -80.788111 | 25.254958 |
| *Pantherophis guttatus* | -81.122458 | 25.273389 |
| *Pantherophis guttatus* | -80.7448 | 25.3292 |
| *Pantherophis guttatus* | -80.416894 | 25.349411 |
| *Pantherophis guttatus* | -80.71829 | 25.38937 |
| *Pantherophis guttatus* | -80.669983 | 25.40065 |
| *Pantherophis guttatus* | -80.56581 | 25.40107 |
| *Pantherophis guttatus* | -80.5 | 25.42 |
| *Pantherophis guttatus* | -80.679625 | 25.423275 |
| *Pantherophis guttatus* | -80.4694 | 25.4275 |
| *Pantherophis guttatus* | -80.4323 | 25.4375 |
| *Pantherophis guttatus* | -80.56165 | 25.462369 |
| *Pantherophis guttatus* | -80.48 | 25.47 |
| *Pantherophis guttatus* | -80.359358 | 25.555319 |
| *Pantherophis guttatus* | -80.685247 | 25.575583 |
| *Pantherophis guttatus* | -80.317892 | 25.581139 |
| *Pantherophis guttatus* | -80.4678 | 25.6 |
| *Pantherophis guttatus* | -80.3539 | 25.6075 |
| *Pantherophis guttatus* | -80.420956 | 25.684542 |
| *Pantherophis guttatus* | -80.4128 | 25.68522 |
| *Pantherophis guttatus* | -80.35169 | 25.74706 |
| *Pantherophis guttatus* | -80.8729 | 25.7608 |
| *Pantherophis guttatus* | -80.66166 | 25.76094 |
| *Pantherophis guttatus* | -80.434008 | 25.761906 |
| *Pantherophis guttatus* | -80.1939 | 25.7739 |
| *Pantherophis guttatus* | -80.175 | 25.8 |
| *Pantherophis guttatus* | -80.289506 | 25.828367 |
| *Pantherophis guttatus* | -80.204978 | 25.832736 |
| *Pantherophis guttatus* | -80.22146 | 25.85034 |
| *Pantherophis guttatus* | -80.221203 | 25.850489 |
| *Pantherophis guttatus* | -81.0202 | 25.8553 |
| *Pantherophis guttatus* | -80.3205 | 25.8721 |
| *Pantherophis guttatus* | -81.2391 | 25.878 |
| *Pantherophis guttatus* | -80.263292 | 25.897894 |
| *Pantherophis guttatus* | -80.190386 | 25.925633 |
| *Pantherophis guttatus* | -81.597764 | 25.995367 |
| *Pantherophis guttatus* | -81.745 | 26.027 |
| *Pantherophis guttatus* | -81.66 | 26.0385 |
| *Pantherophis guttatus* | -81.646547 | 26.043403 |
| *Pantherophis guttatus* | -80.1442 | 26.0519 |
| *Pantherophis guttatus* | -80.125061 | 26.058764 |
| *Pantherophis guttatus* | -80.48338 | 26.13522 |
| *Pantherophis guttatus* | -81.785003 | 26.140761 |
| *Pantherophis guttatus* | -80.374586 | 26.142858 |
| *Pantherophis guttatus* | -81.684958 | 26.153692 |
| *Pantherophis guttatus* | -81.669094 | 26.206147 |
| *Pantherophis guttatus* | -81.666431 | 26.314494 |
| *Pantherophis guttatus* | -80.2422 | 26.3386 |
| *Pantherophis guttatus* | -81.779 | 26.3405 |
| *Pantherophis guttatus* | -81.56547 | 26.3513 |
| *Pantherophis guttatus* | -80.08299 | 26.3587 |
| *Pantherophis guttatus* | -81.603697 | 26.374836 |
| *Pantherophis guttatus* | -81.364058 | 26.474197 |
| *Pantherophis guttatus* | -81.2248 | 26.5143 |
| *Pantherophis guttatus* | -81.12627 | 26.51492 |
| *Pantherophis guttatus* | -81.854683 | 26.547331 |
| *Pantherophis guttatus* | -80.98177 | 26.56073 |
| *Pantherophis guttatus* | -81.226306 | 26.606447 |
| *Pantherophis guttatus* | -81.888 | 26.666 |
| *Pantherophis guttatus* | -82.039094 | 26.686681 |
| *Pantherophis guttatus* | -82.0392 | 26.7052 |
| *Pantherophis guttatus* | -80.0367 | 26.7053 |
| *Pantherophis guttatus* | -81.128883 | 26.74805 |
| *Pantherophis guttatus* | -81.341692 | 26.895725 |
| *Pantherophis guttatus* | -81.314619 | 26.927136 |
| *Pantherophis guttatus* | -81.351464 | 27.18255 |
| *Pantherophis guttatus* | -81.3378 | 27.1883 |
| *Pantherophis guttatus* | -80.829783 | 27.243935 |
| *Pantherophis guttatus* | -81.362933 | 27.293033 |
| *Pantherophis guttatus* | -80.543845 | 27.322556 |
| *Pantherophis guttatus* | -82.335111 | 27.478788 |
| *Pantherophis guttatus* | -82.4236 | 27.6009 |
| *Pantherophis guttatus* | -80.397275 | 27.638642 |
| *Pantherophis guttatus* | -80.39689 | 27.63869 |
| *Pantherophis guttatus* | -80.64319 | 27.66498 |
| *Pantherophis guttatus* | -80.643 | 27.67797 |
| *Pantherophis guttatus* | -82.782369 | 27.837108 |
| *Pantherophis guttatus* | -82.4586 | 27.9472 |
| *Pantherophis guttatus* | -82.793 | 27.967 |
| *Pantherophis guttatus* | -81.734211 | 27.997392 |
| *Pantherophis guttatus* | -81.731803 | 28.022356 |
| *Pantherophis guttatus* | -81.83684 | 28.03787 |
| *Pantherophis guttatus* | -81.94981 | 28.03946 |
| *Pantherophis guttatus* | -81.935494 | 28.041889 |
| *Pantherophis guttatus* | -80.750514 | 28.080394 |
| *Pantherophis guttatus* | -82.293186 | 28.123567 |
| *Pantherophis guttatus* | -82.2586 | 28.1419 |
| *Pantherophis guttatus* | -82.74015 | 28.17667 |
| *Pantherophis guttatus* | -81.953997 | 28.257778 |
| *Pantherophis guttatus* | -82.445944 | 28.260186 |
| *Pantherophis guttatus* | -82.1959 | 28.36473 |
| *Pantherophis guttatus* | -82.098067 | 28.487983 |
| *Pantherophis guttatus* | -82.2716 | 28.5231 |
| *Pantherophis guttatus* | -81.522958 | 28.527853 |
| *Pantherophis guttatus* | -81.379272 | 28.538339 |
| *Pantherophis guttatus* | -82.3542 | 28.6166 |
| *Pantherophis guttatus* | -82.33725 | 28.69112 |
| *Pantherophis guttatus* | -82.307561 | 28.725681 |
| *Pantherophis guttatus* | -81.204753 | 28.790844 |
| *Pantherophis guttatus* | -82.61667 | 28.83333 |
| *Pantherophis guttatus* | -82.313469 | 28.879692 |
| *Pantherophis guttatus* | -81.970486 | 29.061067 |
| *Pantherophis guttatus* | -81.71741 | 29.17806 |
| *Pantherophis guttatus* | -81.551667 | 29.185411 |
| *Pantherophis guttatus* | -81.018893 | 29.208847 |
| *Pantherophis guttatus* | -82.057681 | 29.216401 |
| *Pantherophis guttatus* | -82.938183 | 29.237111 |
| *Pantherophis guttatus* | -81.77124 | 29.29384 |
| *Pantherophis guttatus* | -82.797717 | 29.304531 |
| *Pantherophis guttatus* | -82.187753 | 29.346378 |
| *Pantherophis guttatus* | -82.1808 | 29.3812 |
| *Pantherophis guttatus* | -81.63869 | 29.39138 |
| *Pantherophis guttatus* | -82.449164 | 29.39305 |
| *Pantherophis guttatus* | -82.11 | 29.41167 |
| *Pantherophis guttatus* | -81.380844 | 29.482433 |
| *Pantherophis guttatus* | -82.390939 | 29.556636 |
| *Pantherophis guttatus* | -82.25336 | 29.5655 |
| *Pantherophis guttatus* | -82.333014 | 29.583711 |
| *Pantherophis guttatus* | -91.516641 | 29.588226 |
| *Pantherophis guttatus* | -82.390736 | 29.603753 |
| *Pantherophis guttatus* | -82.81685 | 29.607178 |
| *Pantherophis guttatus* | -81.778702 | 29.626516 |
| *Pantherophis guttatus* | -81.675 | 29.63 |
| *Pantherophis guttatus* | -85.13481 | 29.64263 |
| *Pantherophis guttatus* | -81.628692 | 29.652189 |
| *Pantherophis guttatus* | -85.173297 | 29.672456 |
| *Pantherophis guttatus* | -85.117408 | 29.675286 |
| *Pantherophis guttatus* | -85.30193 | 29.68795 |
| *Pantherophis guttatus* | -85.30749 | 29.69658 |
| *Pantherophis guttatus* | -85.2981 | 29.711131 |
| *Pantherophis guttatus* | -82.453361 | 29.731475 |
| *Pantherophis guttatus* | -85.005828 | 29.733819 |
| *Pantherophis guttatus* | -84.986 | 29.735 |
| *Pantherophis guttatus* | -85.301214 | 29.76605 |
| *Pantherophis guttatus* | -84.831992 | 29.783031 |
| *Pantherophis guttatus* | -83.316208 | 29.815153 |
| *Pantherophis guttatus* | -84.88138 | 29.82939 |
| *Pantherophis guttatus* | -84.702469 | 29.842519 |
| *Pantherophis guttatus* | -91.784244 | 29.900611 |
| *Pantherophis guttatus* | -85.1917 | 29.9338 |
| *Pantherophis guttatus* | -83.244939 | 29.952661 |
| *Pantherophis guttatus* | -81.6764 | 29.989844 |
| *Pantherophis guttatus* | -85 | 30 |
| *Pantherophis guttatus* | -85.25838 | 30.0507 |
| *Pantherophis guttatus* | -90.796303 | 30.070756 |
| *Pantherophis guttatus* | -82 | 30.080358 |
| *Pantherophis guttatus* | -83.677265 | 30.097719 |
| *Pantherophis guttatus* | -85.092 | 30.10413 |
| *Pantherophis guttatus* | -84.388972 | 30.132694 |
| *Pantherophis guttatus* | -85.210081 | 30.141097 |
| *Pantherophis guttatus* | -85.65675 | 30.163286 |
| *Pantherophis guttatus* | -90.996044 | 30.169031 |
| *Pantherophis guttatus* | -84.652686 | 30.191525 |
| *Pantherophis guttatus* | -84.05022 | 30.19585 |
| *Pantherophis guttatus* | -88.020765 | 30.227897 |
| *Pantherophis guttatus* | -89.766417 | 30.24685 |
| *Pantherophis guttatus* | -90.768678 | 30.288344 |
| *Pantherophis guttatus* | -85.018386 | 30.290331 |
| *Pantherophis guttatus* | -84.40043 | 30.30377 |
| *Pantherophis guttatus* | -89.902285 | 30.309948 |
| *Pantherophis guttatus* | -90.856694 | 30.319194 |
| *Pantherophis guttatus* | -89.596 | 30.321 |
| *Pantherophis guttatus* | -91.014647 | 30.328106 |
| *Pantherophis guttatus* | -89.399139 | 30.349156 |
| *Pantherophis guttatus* | -84.886306 | 30.349878 |
| *Pantherophis guttatus* | -91.371575 | 30.358392 |
| *Pantherophis guttatus* | -91.012722 | 30.358811 |
| *Pantherophis guttatus* | -91.135508 | 30.365675 |
| *Pantherophis guttatus* | -84.362944 | 30.368389 |
| *Pantherophis guttatus* | -84.63912 | 30.37516 |
| *Pantherophis guttatus* | -91.017414 | 30.382928 |
| *Pantherophis guttatus* | -91.150736 | 30.387486 |
| *Pantherophis guttatus* | -84.6365 | 30.387542 |
| *Pantherophis guttatus* | -91.120608 | 30.396764 |
| *Pantherophis guttatus* | -85.121433 | 30.398669 |
| *Pantherophis guttatus* | -86.617042 | 30.419729 |
| *Pantherophis guttatus* | -84.31657 | 30.43353 |
| *Pantherophis guttatus* | -91.414714 | 30.4373 |
| *Pantherophis guttatus* | -84.280722 | 30.438219 |
| *Pantherophis guttatus* | -84.32651 | 30.45351 |
| *Pantherophis guttatus* | -93.036017 | 30.455322 |
| *Pantherophis guttatus* | -82.913392 | 30.474458 |
| *Pantherophis guttatus* | -90.461172 | 30.504392 |
| *Pantherophis guttatus* | -90.3728 | 30.510944 |
| *Pantherophis guttatus* | -82.019417 | 30.525856 |
| *Pantherophis guttatus* | -91.199822 | 30.525939 |
| *Pantherophis guttatus* | -81.842683 | 30.545057 |
| *Pantherophis guttatus* | -87.891619 | 30.547794 |
| *Pantherophis guttatus* | -86.109386 | 30.575597 |
| *Pantherophis guttatus* | -86.659224 | 30.575904 |
| *Pantherophis guttatus* | -84.97765 | 30.59634 |
| *Pantherophis guttatus* | -90.16881 | 30.628216 |
| *Pantherophis guttatus* | -90.313961 | 30.628928 |
| *Pantherophis guttatus* | -85.64841 | 30.63049 |
| *Pantherophis guttatus* | -82.333086 | 30.661008 |
| *Pantherophis guttatus* | -91.073853 | 30.663272 |
| *Pantherophis guttatus* | -85.955919 | 30.668697 |
| *Pantherophis guttatus* | -91.269656 | 30.676894 |
| *Pantherophis guttatus* | -86.122322 | 30.686228 |
| *Pantherophis guttatus* | -81.910272 | 30.697664 |
| *Pantherophis guttatus* | -91.436114 | 30.701275 |
| *Pantherophis guttatus* | -90.804133 | 30.703356 |
| *Pantherophis guttatus* | -90.754675 | 30.712344 |
| *Pantherophis guttatus* | -81.90843 | 30.71679 |
| *Pantherophis guttatus* | -91.204808 | 30.734325 |
| *Pantherophis guttatus* | -92.929775 | 30.762517 |
| *Pantherophis guttatus* | -85.227069 | 30.774167 |
| *Pantherophis guttatus* | -81.547756 | 30.789375 |
| *Pantherophis guttatus* | -86.843592 | 30.790275 |
| *Pantherophis guttatus* | -92.286572 | 30.790597 |
| *Pantherophis guttatus* | -85.683461 | 30.792425 |
| *Pantherophis guttatus* | -86.944081 | 30.795964 |
| *Pantherophis guttatus* | -82.305119 | 30.798819 |
| *Pantherophis guttatus* | -90.405244 | 30.8293 |
| *Pantherophis guttatus* | -82.3039 | 30.8385 |
| *Pantherophis guttatus* | -81.669347 | 30.840494 |
| *Pantherophis guttatus* | -91.373856 | 30.849417 |
| *Pantherophis guttatus* | -90.684379 | 30.85401 |
| *Pantherophis guttatus* | -83.304935 | 30.861381 |
| *Pantherophis guttatus* | -86.25168 | 30.86234 |
| *Pantherophis guttatus* | -81.531 | 30.863 |
| *Pantherophis guttatus* | -81.971325 | 30.883275 |
| *Pantherophis guttatus* | -84.229963 | 30.904953 |
| *Pantherophis guttatus* | -91.378058 | 30.929303 |
| *Pantherophis guttatus* | -91.424175 | 30.931158 |
| *Pantherophis guttatus* | -90.536503 | 30.936261 |
| *Pantherophis guttatus* | -82.80865 | 30.971336 |
| *Pantherophis guttatus* | -91.442925 | 30.997281 |
| *Pantherophis guttatus* | -81.428111 | 31.015464 |
| *Pantherophis guttatus* | -82.867767 | 31.034894 |
| *Pantherophis guttatus* | -88.552647 | 31.048356 |
| *Pantherophis guttatus* | -82.657753 | 31.068903 |
| *Pantherophis guttatus* | -86.5788 | 31.075114 |
| *Pantherophis guttatus* | -81.862681 | 31.138664 |
| *Pantherophis guttatus* | -84.773342 | 31.161753 |
| *Pantherophis guttatus* | -81.381597 | 31.174828 |
| *Pantherophis guttatus* | -81.3719 | 31.2225 |
| *Pantherophis guttatus* | -93.407836 | 31.241306 |
| *Pantherophis guttatus* | -81.964769 | 31.259819 |
| *Pantherophis guttatus* | -82.854911 | 31.299961 |
| *Pantherophis guttatus* | -92.946164 | 31.306322 |
| *Pantherophis guttatus* | -82.944803 | 31.313439 |
| *Pantherophis guttatus* | -93.194339 | 31.326722 |
| *Pantherophis guttatus* | -92.877658 | 31.331883 |
| *Pantherophis guttatus* | -81.451217 | 31.342522 |
| *Pantherophis guttatus* | -92.586403 | 31.362366 |
| *Pantherophis guttatus* | -93.4189 | 31.376253 |
| *Pantherophis guttatus* | -91.28795 | 31.41315 |
| *Pantherophis guttatus* | -91.282296 | 31.416471 |
| *Pantherophis guttatus* | -93.319581 | 31.464558 |
| *Pantherophis guttatus* | -93.231503 | 31.467419 |
| *Pantherophis guttatus* | -81.241597 | 31.475964 |
| *Pantherophis guttatus* | -81.343928 | 31.477986 |
| *Pantherophis guttatus* | -93.015722 | 31.481072 |
| *Pantherophis guttatus* | -81.638947 | 31.494433 |
| *Pantherophis guttatus* | -82.050133 | 31.498025 |
| *Pantherophis guttatus* | -81.960425 | 31.500297 |
| *Pantherophis guttatus* | -81.751661 | 31.507731 |
| *Pantherophis guttatus* | -91.35725 | 31.538977 |
| *Pantherophis guttatus* | -91.38275 | 31.54952 |
| *Pantherophis guttatus* | -81.420089 | 31.571125 |
| *Pantherophis guttatus* | -92.950469 | 31.583786 |
| *Pantherophis guttatus* | -81.980164 | 31.649517 |
| *Pantherophis guttatus* | -81.396611 | 31.656881 |
| *Pantherophis guttatus* | -81.65025 | 31.688656 |
| *Pantherophis guttatus* | -92.561417 | 31.721283 |
| *Pantherophis guttatus* | -82.092939 | 31.731894 |
| *Pantherophis guttatus* | -81.441403 | 31.744597 |
| *Pantherophis guttatus* | -81.951172 | 31.754789 |
| *Pantherophis guttatus* | -81.405411 | 31.796028 |
| *Pantherophis guttatus* | -81.244003 | 31.800853 |
| *Pantherophis guttatus* | -81.247239 | 31.847436 |
| *Pantherophis guttatus* | -83.059028 | 31.849433 |
| *Pantherophis guttatus* | -81.983683 | 31.874753 |
| *Pantherophis guttatus* | -81.583903 | 31.879078 |
| *Pantherophis guttatus* | -80.982836 | 31.905442 |
| *Pantherophis guttatus* | -81.647019 | 31.942306 |
| *Pantherophis guttatus* | -81.090333 | 31.954269 |
| *Pantherophis guttatus* | -81.339461 | 31.958853 |
| *Pantherophis guttatus* | -81.744462 | 31.963682 |
| *Pantherophis guttatus* | -81.218928 | 31.976825 |
| *Pantherophis guttatus* | -81.471339 | 31.977244 |
| *Pantherophis guttatus* | -81.284317 | 31.981089 |
| *Pantherophis guttatus* | -81.124861 | 31.990367 |
| *Pantherophis guttatus* | -81.150342 | 32.002131 |
| *Pantherophis guttatus* | -80.971178 | 32.007047 |
| *Pantherophis guttatus* | -91.006175 | 32.012006 |
| *Pantherophis guttatus* | -81.09565 | 32.017994 |
| *Pantherophis guttatus* | -81.584683 | 32.018233 |
| *Pantherophis guttatus* | -80.893725 | 32.027656 |
| *Pantherophis guttatus* | -80.974278 | 32.032436 |
| *Pantherophis guttatus* | -81.039556 | 32.037711 |
| *Pantherophis guttatus* | -81.264014 | 32.046019 |
| *Pantherophis guttatus* | -81.053639 | 32.066011 |
| *Pantherophis guttatus* | -81.135275 | 32.068203 |
| *Pantherophis guttatus* | -82.147928 | 32.079619 |
| *Pantherophis guttatus* | -81.075747 | 32.082619 |
| *Pantherophis guttatus* | -81.193353 | 32.106869 |
| *Pantherophis guttatus* | -80.86445 | 32.118853 |
| *Pantherophis guttatus* | -81.279114 | 32.120797 |
| *Pantherophis guttatus* | -81.617025 | 32.126175 |
| *Pantherophis guttatus* | -81.513414 | 32.127203 |
| *Pantherophis guttatus* | -81.377031 | 32.131664 |
| *Pantherophis guttatus* | -82.155533 | 32.226058 |
| *Pantherophis guttatus* | -81.439639 | 32.231936 |
| *Pantherophis guttatus* | -82.072001 | 32.232592 |
| *Pantherophis guttatus* | -80.859311 | 32.241198 |
| *Pantherophis guttatus* | -82.503664 | 32.269089 |
| *Pantherophis guttatus* | -92.990572 | 32.273158 |
| *Pantherophis guttatus* | -82.407681 | 32.274344 |
| *Pantherophis guttatus* | -82.226265 | 32.285107 |
| *Pantherophis guttatus* | -82.697428 | 32.293311 |
| *Pantherophis guttatus* | -81.100626 | 32.296777 |
| *Pantherophis guttatus* | -92.576053 | 32.306247 |
| *Pantherophis guttatus* | -89.667264 | 32.319453 |
| *Pantherophis guttatus* | -92.635611 | 32.320331 |
| *Pantherophis guttatus* | -93.205147 | 32.346911 |
| *Pantherophis guttatus* | -82.140633 | 32.365097 |
| *Pantherophis guttatus* | -92.571572 | 32.388712 |
| *Pantherophis guttatus* | -80.914152 | 32.388902 |
| *Pantherophis guttatus* | -81.009 | 32.4324 |
| *Pantherophis guttatus* | -82.063825 | 32.458022 |
| *Pantherophis guttatus* | -80.883683 | 32.481838 |
| *Pantherophis guttatus* | -82.926022 | 32.489297 |
| *Pantherophis guttatus* | -81.075391 | 32.490483 |
| *Pantherophis guttatus* | -81.493047 | 32.495056 |
| *Pantherophis guttatus* | -80.981254 | 32.510591 |
| *Pantherophis guttatus* | -86.498178 | 32.537139 |
| *Pantherophis guttatus* | -81.162815 | 32.538431 |
| *Pantherophis guttatus* | -81.021708 | 32.5413 |
| *Pantherophis guttatus* | -84.708189 | 32.547658 |
| *Pantherophis guttatus* | -81.520272 | 32.547864 |
| *Pantherophis guttatus* | -81.504053 | 32.591525 |
| *Pantherophis guttatus* | -89.682286 | 32.707228 |
| *Pantherophis guttatus* | -88.64228 | 32.74613 |
| *Pantherophis guttatus* | -79.952 | 32.76 |
| *Pantherophis guttatus* | -82.890442 | 32.764756 |
| *Pantherophis guttatus* | -89.686644 | 32.792128 |
| *Pantherophis guttatus* | -88.710881 | 32.801872 |
| *Pantherophis guttatus* | -81.603397 | 32.824019 |
| *Pantherophis guttatus* | -83.078778 | 32.825778 |
| *Pantherophis guttatus* | -87.8868 | 32.842356 |
| *Pantherophis guttatus* | -81.602969 | 32.870275 |
| *Pantherophis guttatus* | -82.471472 | 32.876928 |
| *Pantherophis guttatus* | -87.440175 | 32.887167 |
| *Pantherophis guttatus* | -82.693908 | 32.899575 |
| *Pantherophis guttatus* | -82.902769 | 32.978036 |
| *Pantherophis guttatus* | -81.336269 | 33.002147 |
| *Pantherophis guttatus* | -82.019761 | 33.067983 |
| *Pantherophis guttatus* | -89.51245 | 33.107853 |
| *Pantherophis guttatus* | -81.479211 | 33.183336 |
| *Pantherophis guttatus* | -82.784858 | 33.189061 |
| *Pantherophis guttatus* | -82.370825 | 33.201422 |
| *Pantherophis guttatus* | -87.467419 | 33.207864 |
| *Pantherophis guttatus* | -87.538919 | 33.219547 |
| *Pantherophis guttatus* | -81.910331 | 33.236703 |
| *Pantherophis guttatus* | -86.123583 | 33.241486 |
| *Pantherophis guttatus* | -87.473858 | 33.272469 |
| *Pantherophis guttatus* | -81.042658 | 33.302061 |
| *Pantherophis guttatus* | -81.73773 | 33.3492 |
| *Pantherophis guttatus* | -82.0718 | 33.3639 |
| *Pantherophis guttatus* | -84.491006 | 33.371378 |
| *Pantherophis guttatus* | -87.604814 | 33.418703 |
| *Pantherophis guttatus* | -82.449883 | 33.437519 |
| *Pantherophis guttatus* | -81.68734 | 33.54979 |
| *Pantherophis guttatus* | -81.738206 | 33.553817 |
| *Pantherophis guttatus* | -78.898659 | 33.685114 |
| *Pantherophis guttatus* | -87.507114 | 33.791961 |
| *Pantherophis guttatus* | -78.041469 | 33.927156 |
| *Pantherophis guttatus* | -88.90265 | 33.988836 |
| *Pantherophis guttatus* | -82.63861 | 33.98944 |
| *Pantherophis guttatus* | -81.049132 | 34.011558 |
| *Pantherophis guttatus* | -78.290728 | 34.031542 |
| *Pantherophis guttatus* | -87.390119 | 34.142875 |
| *Pantherophis guttatus* | -85.355239 | 34.200672 |
| *Pantherophis guttatus* | -84.616511 | 34.222906 |
| *Pantherophis guttatus* | -77.944711 | 34.225725 |
| *Pantherophis guttatus* | -84.601569 | 34.304619 |
| *Pantherophis guttatus* | -84.611258 | 34.388375 |
| *Pantherophis guttatus* | -89.662483 | 34.452814 |
| *Pantherophis guttatus* | -77.81351 | 34.52427 |
| *Pantherophis guttatus* | -77.9261 | 34.5521 |
| *Pantherophis guttatus* | -85.341175 | 34.552272 |
| *Pantherophis guttatus* | -89.574067 | 34.579528 |
| *Pantherophis guttatus* | -85.352442 | 34.645394 |
| *Pantherophis guttatus* | -88.960173 | 34.729039 |
| *Pantherophis guttatus* | -76.768 | 34.7763 |
| *Pantherophis guttatus* | -79 | 34.795148 |
| *Pantherophis guttatus* | -78.583531 | 34.799556 |
| *Pantherophis guttatus* | -76.569783 | 34.800839 |
| *Pantherophis guttatus* | -83.404344 | 34.877778 |
| *Pantherophis guttatus* | -77.034172 | 34.948556 |
| *Pantherophis guttatus* | -85.065436 | 35.057256 |
| *Pantherophis guttatus* | -77.048583 | 35.100919 |
| *Pantherophis guttatus* | -85.739004 | 35.162327 |
| *Pantherophis guttatus* | -75.565683 | 35.255447 |
| *Pantherophis guttatus* | -88.952557 | 35.352303 |
| *Pantherophis guttatus* | -88.953859 | 35.355185 |
| *Pantherophis guttatus* | -75.487919 | 35.414131 |
| *Pantherophis guttatus* | -75.483592 | 35.464506 |
| *Pantherophis guttatus* | -76.54614 | 35.48701 |
| *Pantherophis guttatus* | -75.47 | 35.54 |
| *Pantherophis guttatus* | -84.090028 | 35.567272 |
| *Pantherophis guttatus* | -83.6675 | 35.621303 |
| *Pantherophis guttatus* | -83.758008 | 35.677267 |
| *Pantherophis guttatus* | -84.034047 | 35.686853 |
| *Pantherophis guttatus* | -84.33311 | 35.72394 |
| *Pantherophis guttatus* | -85.077517 | 35.737247 |
| *Pantherophis guttatus* | -75.794631 | 35.866392 |
| *Pantherophis guttatus* | -76.18624 | 35.92945 |
| *Pantherophis guttatus* | -76.183378 | 35.929494 |
| *Pantherophis guttatus* | -83.121615 | 35.935528 |
| *Pantherophis guttatus* | -79.791975 | 36.072636 |
| *Pantherophis guttatus* | -84.103208 | 36.112569 |
| *Pantherophis guttatus* | -79.869833 | 36.782842 |
| *Pantherophis guttatus* | -80.785175 | 36.96235 |
| *Pantherophis guttatus* | -86.062839 | 37.092806 |
| *Pantherophis guttatus* | -80.184717 | 37.1193 |
| *Pantherophis guttatus* | -86.027469 | 37.146497 |
| *Pantherophis guttatus* | -86.215625 | 37.193869 |
| *Pantherophis guttatus* | -80.3254 | 37.1956 |
| *Pantherophis guttatus* | -79.865142 | 37.210564 |
| *Pantherophis guttatus* | -80.30645 | 37.21183 |
| *Pantherophis guttatus* | -77.502864 | 37.295278 |
| *Pantherophis guttatus* | -85.762092 | 37.309483 |
| *Pantherophis guttatus* | -80.115442 | 37.379442 |
| *Pantherophis guttatus* | -79.651392 | 37.397906 |
| *Pantherophis guttatus* | -85.999136 | 37.410611 |
| *Pantherophis guttatus* | -79.879717 | 37.411306 |
| *Pantherophis guttatus* | -77.903644 | 37.425222 |
| *Pantherophis guttatus* | -79.74 | 37.43 |
| *Pantherophis guttatus* | -78.682947 | 37.4515 |
| *Pantherophis guttatus* | -78.006558 | 37.459817 |
| *Pantherophis guttatus* | -78.9047 | 37.4913 |
| *Pantherophis guttatus* | -86.022942 | 37.513692 |
| *Pantherophis guttatus* | -77.6337 | 37.517225 |
| *Pantherophis guttatus* | -78.832153 | 37.534028 |
| *Pantherophis guttatus* | -79.045303 | 37.578425 |
| *Pantherophis guttatus* | -77.748522 | 37.594436 |
| *Pantherophis guttatus* | -77.698364 | 37.60085 |
| *Pantherophis guttatus* | -77.723244 | 37.603683 |
| *Pantherophis guttatus* | -78.936933 | 37.616758 |
| *Pantherophis guttatus* | -78.726475 | 37.641567 |
| *Pantherophis guttatus* | -79.796936 | 37.661139 |
| *Pantherophis guttatus* | -79.240931 | 37.690033 |
| *Pantherophis guttatus* | -77.310814 | 37.694361 |
| *Pantherophis guttatus* | -78.317808 | 37.701733 |
| *Pantherophis guttatus* | -77.602153 | 37.753775 |
| *Pantherophis guttatus* | -83.464108 | 37.754856 |
| *Pantherophis guttatus* | -83.528219 | 37.827192 |
| *Pantherophis guttatus* | -79.836339 | 37.829525 |
| *Pantherophis guttatus* | -83.634503 | 37.933511 |
| *Pantherophis guttatus* | -77.909767 | 38.01175 |
| *Pantherophis guttatus* | -78.610146 | 38.048248 |
| *Pantherophis guttatus* | -76.537507 | 38.222062 |
| *Pantherophis guttatus* | -77.954278 | 38.295619 |
| *Pantherophis guttatus* | -76.589605 | 38.297377 |
| *Pantherophis guttatus* | -78.665958 | 38.317653 |
| *Pantherophis guttatus* | -78.4738 | 38.3286 |
| *Pantherophis guttatus* | -82.949278 | 38.329825 |
| *Pantherophis guttatus* | -76.71471 | 38.4938 |
| *Pantherophis guttatus* | -75.625931 | 38.523606 |
| *Pantherophis guttatus* | -84.398864 | 38.602789 |
| *Pantherophis guttatus* | -75.631589 | 38.612292 |
| *Pantherophis guttatus* | -78.225033 | 38.657592 |
| *Pantherophis guttatus* | -76.721703 | 38.679131 |
| *Pantherophis guttatus* | -75.169 | 38.708 |
| *Pantherophis guttatus* | -76.7043 | 38.7096 |
| *Pantherophis guttatus* | -78.093686 | 38.72295 |
| *Pantherophis guttatus* | -75.194 | 38.776 |
| *Pantherophis guttatus* | -78.427561 | 38.842369 |
| *Pantherophis guttatus* | -78.175225 | 38.927794 |
| *Pantherophis guttatus* | -77.0764 | 38.9712 |
| *Pantherophis guttatus* | -74.9445 | 39.372492 |
| *Pantherophis guttatus* | -76.969861 | 39.546525 |
| *Pantherophis guttatus* | -79.099231 | 39.588161 |
| *Pantherophis guttatus* | -78.3786 | 39.59369 |
| *Pantherophis guttatus* | -74.705931 | 39.844803 |
| *Pantherophis guttatus* | -74.029919 | 40.246758 |
| *Pantherophis guttatus* | -74.405394 | 40.057892 |
| *Pantherophis guttatus* | -74.034609 | 40.19862 |
| *Pantherophis guttatus* | -64.633064 | 18.432292 |

**S1C Dataset**

Occurrence records of *Lampropeltis triangulum* in the Americas.

| Specie | Longitude | Latitude |
| --- | --- | --- |
| *Lampropeltis triangulum* | -79.9 | -2.16667 |
| *Lampropeltis triangulum* | -79.9 | -1.36667 |
| *Lampropeltis triangulum* | -79.583 | -1.333 |
| *Lampropeltis triangulum* | -79.15 | -0.25 |
| *Lampropeltis triangulum* | -79.22528 | -0.23556 |
| *Lampropeltis triangulum* | -76.616857 | 2.45019 |
| *Lampropeltis triangulum* | -76.492559 | 3.37415 |
| *Lampropeltis triangulum* | -76.55 | 3.41667 |
| *Lampropeltis triangulum* | -76.4742 | 3.6353 |
| *Lampropeltis triangulum* | -76.641149 | 3.800012 |
| *Lampropeltis triangulum* | -74.58333 | 4.25 |
| *Lampropeltis triangulum* | -77.00432 | 4.55388 |
| *Lampropeltis triangulum* | -75.75 | 4.83333 |
| *Lampropeltis triangulum* | -74.41667 | 5.41667 |
| *Lampropeltis triangulum* | -74.27135 | 5.60052 |
| *Lampropeltis triangulum* | -75.83333 | 5.83333 |
| *Lampropeltis triangulum* | -75.53646 | 6.2924 |
| *Lampropeltis triangulum* | -72.5 | 6.33333 |
| *Lampropeltis triangulum* | -72.16667 | 6.75 |
| *Lampropeltis triangulum* | -75.25 | 7.08333 |
| *Lampropeltis triangulum* | -72.58333 | 7.41667 |
| *Lampropeltis triangulum* | -73.66667 | 7.58333 |
| *Lampropeltis triangulum* | -80.13333 | 8.6 |
| *Lampropeltis triangulum* | -82.413437 | 8.60385 |
| *Lampropeltis triangulum* | -71.33947 | 8.61344 |
| *Lampropeltis triangulum* | -82.966667 | 8.8 |
| *Lampropeltis triangulum* | -82.95 | 8.85 |
| *Lampropeltis triangulum* | -82.7493 | 8.88478 |
| *Lampropeltis triangulum* | -82.85 | 8.933333 |
| *Lampropeltis triangulum* | -82.75 | 8.95 |
| *Lampropeltis triangulum* | -82.726732 | 9.372581 |
| *Lampropeltis triangulum* | -83.71453 | 9.43477 |
| *Lampropeltis triangulum* | -83.75 | 9.566667 |
| *Lampropeltis triangulum* | -83.95 | 9.7333 |
| *Lampropeltis triangulum* | -83.9667 | 9.8 |
| *Lampropeltis triangulum* | -83.6667 | 9.8667 |
| *Lampropeltis triangulum* | -84.30791 | 10.0167 |
| *Lampropeltis triangulum* | -84.75 | 10.03333 |
| *Lampropeltis triangulum* | -84.45 | 10.06667 |
| *Lampropeltis triangulum* | -84.16984 | 10.23698 |
| *Lampropeltis triangulum* | -84.18388 | 10.2678 |
| *Lampropeltis triangulum* | -84.78333 | 10.28333 |
| *Lampropeltis triangulum* | -84.816666 | 10.3 |
| *Lampropeltis triangulum* | -84.43333 | 10.33333 |
| *Lampropeltis triangulum* | -84.02535 | 10.4221 |
| *Lampropeltis triangulum* | -83.98333 | 10.43333 |
| *Lampropeltis triangulum* | -85.12972 | 10.4531 |
| *Lampropeltis triangulum* | -85.24862 | 10.52037 |
| *Lampropeltis triangulum* | -85.681 | 10.537 |
| *Lampropeltis triangulum* | -84.667576 | 10.83115 |
| *Lampropeltis triangulum* | -83.691076 | 10.932547 |
| *Lampropeltis triangulum* | -85.616783 | 11.09782 |
| *Lampropeltis triangulum* | -86.14445 | 11.90695 |
| *Lampropeltis triangulum* | -86.164722 | 12.138187 |
| *Lampropeltis triangulum* | -89.49583 | 13.73333 |
| *Lampropeltis triangulum* | -88.11667 | 13.91667 |
| *Lampropeltis triangulum* | -87.010201 | 14.0076 |
| *Lampropeltis triangulum* | -90.785 | 14.305 |
| *Lampropeltis triangulum* | -91.753881 | 14.372716 |
| *Lampropeltis triangulum* | -88.983 | 14.417 |
| *Lampropeltis triangulum* | -85.3 | 14.65 |
| *Lampropeltis triangulum* | -84.467 | 14.783 |
| *Lampropeltis triangulum* | -91.52257 | 14.829885 |
| *Lampropeltis triangulum* | -89.1333 | 14.85 |
| *Lampropeltis triangulum* | -85.905113 | 14.859068 |
| *Lampropeltis triangulum* | -91.832292 | 14.94846 |
| *Lampropeltis triangulum* | -88.02083 | 14.96389 |
| *Lampropeltis triangulum* | -92.135 | 15.042 |
| *Lampropeltis triangulum* | -88.917 | 15.1 |
| *Lampropeltis triangulum* | -90.149882 | 15.166692 |
| *Lampropeltis triangulum* | -92.417 | 15.222 |
| *Lampropeltis triangulum* | -89.099779 | 15.251993 |
| *Lampropeltis triangulum* | -92.233 | 15.366 |
| *Lampropeltis triangulum* | -92.63 | 15.42 |
| *Lampropeltis triangulum* | -87.919159 | 15.432701 |
| *Lampropeltis triangulum* | -88.837248 | 15.474317 |
| *Lampropeltis triangulum* | -89.50001 | 15.53333 |
| *Lampropeltis triangulum* | -87.2994 | 15.714 |
| *Lampropeltis triangulum* | -87.084225 | 15.767556 |
| *Lampropeltis triangulum* | -87.45 | 15.7833 |
| *Lampropeltis triangulum* | -87.772598 | 15.8315 |
| *Lampropeltis triangulum* | -90.196336 | 15.958148 |
| *Lampropeltis triangulum* | -96.637 | 15.959 |
| *Lampropeltis triangulum* | -97.688 | 15.966 |
| *Lampropeltis triangulum* | -95.67 | 15.99 |
| *Lampropeltis triangulum* | -97.52 | 16.01 |
| *Lampropeltis triangulum* | -97.698 | 16.096 |
| *Lampropeltis triangulum* | -91.68 | 16.11 |
| *Lampropeltis triangulum* | -94.22 | 16.41 |
| *Lampropeltis triangulum* | -93.54583 | 16.68611 |
| *Lampropeltis triangulum* | -96.36 | 16.74 |
| *Lampropeltis triangulum* | -93.117 | 16.753 |
| *Lampropeltis triangulum* | -99.34 | 16.76 |
| *Lampropeltis triangulum* | -99.66 | 16.8 |
| *Lampropeltis triangulum* | -93.268891 | 16.801678 |
| *Lampropeltis triangulum* | -93.40417 | 16.80833 |
| *Lampropeltis triangulum* | -91.452 | 16.814 |
| *Lampropeltis triangulum* | -99.69 | 16.82 |
| *Lampropeltis triangulum* | -93.3103 | 16.86728 |
| *Lampropeltis triangulum* | -99.808 | 16.871 |
| *Lampropeltis triangulum* | -91.628 | 16.897 |
| *Lampropeltis triangulum* | -96.41 | 16.93 |
| *Lampropeltis triangulum* | -91.61667 | 16.96667 |
| *Lampropeltis triangulum* | -93.486 | 16.999 |
| *Lampropeltis triangulum* | -88.5 | 17.01667 |
| *Lampropeltis triangulum* | -98.78 | 17.05 |
| *Lampropeltis triangulum* | -99.61194 | 17.12861 |
| *Lampropeltis triangulum* | -88.66842 | 17.15153 |
| *Lampropeltis triangulum* | -93.02 | 17.24 |
| *Lampropeltis triangulum* | -99.544878 | 17.437803 |
| *Lampropeltis triangulum* | -100.18 | 17.45 |
| *Lampropeltis triangulum* | -91.947 | 17.476 |
| *Lampropeltis triangulum* | -92.017 | 17.483 |
| *Lampropeltis triangulum* | -99.48 | 17.52 |
| *Lampropeltis triangulum* | -99.5 | 17.549999 |
| *Lampropeltis triangulum* | -99.45 | 17.55 |
| *Lampropeltis triangulum* | -93.182 | 17.587 |
| *Lampropeltis triangulum* | -96.33 | 17.65 |
| *Lampropeltis triangulum* | -101.61 | 17.72 |
| *Lampropeltis triangulum* | -92.947378 | 17.765852 |
| *Lampropeltis triangulum* | -96.985 | 17.854 |
| *Lampropeltis triangulum* | -95.97 | 17.88 |
| *Lampropeltis triangulum* | -97.018 | 17.902 |
| *Lampropeltis triangulum* | -97.019 | 17.946 |
| *Lampropeltis triangulum* | -92.97 | 17.99 |
| *Lampropeltis triangulum* | -102.305739 | 18.025481 |
| *Lampropeltis triangulum* | -97.067 | 18.133 |
| *Lampropeltis triangulum* | -94.17 | 18.17 |
| *Lampropeltis triangulum* | -97.47 | 18.171 |
| *Lampropeltis triangulum* | -95.15 | 18.18 |
| *Lampropeltis triangulum* | -97.651 | 18.185 |
| *Lampropeltis triangulum* | -97.203 | 18.22 |
| *Lampropeltis triangulum* | -97.469 | 18.283 |
| *Lampropeltis triangulum* | -97.495 | 18.326 |
| *Lampropeltis triangulum* | -95.13 | 18.37 |
| *Lampropeltis triangulum* | -99.547102 | 18.389337 |
| *Lampropeltis triangulum* | -88.305 | 18.504 |
| *Lampropeltis triangulum* | -95.07 | 18.516 |
| *Lampropeltis triangulum* | -98.848932 | 18.555091 |
| *Lampropeltis triangulum* | -95.088267 | 18.590867 |
| *Lampropeltis triangulum* | -90.733 | 18.617 |
| *Lampropeltis triangulum* | -95.56 | 18.69 |
| *Lampropeltis triangulum* | -90.739998 | 18.719999 |
| *Lampropeltis triangulum* | -90.74 | 18.72 |
| *Lampropeltis triangulum* | -103.16 | 18.78 |
| *Lampropeltis triangulum* | -98.438 | 18.862 |
| *Lampropeltis triangulum* | -97.03333 | 18.86667 |
| *Lampropeltis triangulum* | -97.01667 | 18.88333 |
| *Lampropeltis triangulum* | -96.783333 | 18.883333 |
| *Lampropeltis triangulum* | -89.28 | 19.002 |
| *Lampropeltis triangulum* | -98.2 | 19.05 |
| *Lampropeltis triangulum* | -104.29 | 19.07 |
| *Lampropeltis triangulum* | -96.94528 | 19.15833 |
| *Lampropeltis triangulum* | -88.422 | 19.205 |
| *Lampropeltis triangulum* | -103.716667 | 19.233333 |
| *Lampropeltis triangulum* | -104.182416 | 19.242039 |
| *Lampropeltis triangulum* | -104.83 | 19.32 |
| *Lampropeltis triangulum* | -104.96 | 19.41 |
| *Lampropeltis triangulum* | -102.094 | 19.428 |
| *Lampropeltis triangulum* | -102.219 | 19.443 |
| *Lampropeltis triangulum* | -103.46 | 19.47 |
| *Lampropeltis triangulum* | -104.51 | 19.54 |
| *Lampropeltis triangulum* | -103.60669 | 19.578275 |
| *Lampropeltis triangulum* | -88.033 | 19.583 |
| *Lampropeltis triangulum* | -88.14 | 19.61 |
| *Lampropeltis triangulum* | -88 | 19.639999 |
| *Lampropeltis triangulum* | -88 | 19.64 |
| *Lampropeltis triangulum* | -105.21 | 19.7 |
| *Lampropeltis triangulum* | -88.73 | 19.78 |
| *Lampropeltis triangulum* | -96.77 | 19.787 |
| *Lampropeltis triangulum* | -103.68 | 19.94 |
| *Lampropeltis triangulum* | -97.534 | 19.969 |
| *Lampropeltis triangulum* | -103.76 | 20.23 |
| *Lampropeltis triangulum* | -97.96 | 20.276 |
| *Lampropeltis triangulum* | -103.195583 | 20.291563 |
| *Lampropeltis triangulum* | -87.55 | 20.33 |
| *Lampropeltis triangulum* | -96.91 | 20.37 |
| *Lampropeltis triangulum* | -103.56 | 20.43 |
| *Lampropeltis triangulum* | -103.559998 | 20.43 |
| *Lampropeltis triangulum* | -98.272 | 20.443 |
| *Lampropeltis triangulum* | -103.33 | 20.68 |
| *Lampropeltis triangulum* | -88.567 | 20.683 |
| *Lampropeltis triangulum* | -88.199714 | 20.689088 |
| *Lampropeltis triangulum* | -88.68 | 20.71 |
| *Lampropeltis triangulum* | -87.717 | 20.833 |
| *Lampropeltis triangulum* | -101.56667 | 20.83333 |
| *Lampropeltis triangulum* | -87.60802 | 20.87853 |
| *Lampropeltis triangulum* | -87.3 | 20.9 |
| *Lampropeltis triangulum* | -103.91 | 20.9 |
| *Lampropeltis triangulum* | -97.680446 | 20.905011 |
| *Lampropeltis triangulum* | -98.756 | 20.962 |
| *Lampropeltis triangulum* | -103.03 | 21.01 |
| *Lampropeltis triangulum* | -98.508 | 21.059 |
| *Lampropeltis triangulum* | -89.599 | 21.097 |
| *Lampropeltis triangulum* | -89.61667 | 21.1 |
| *Lampropeltis triangulum* | -98.95806 | 21.13417 |
| *Lampropeltis triangulum* | -87.183 | 21.217 |
| *Lampropeltis triangulum* | -99.486498 | 21.259149 |
| *Lampropeltis triangulum* | -99.686926 | 21.427524 |
| *Lampropeltis triangulum* | -99.93 | 21.8 |
| *Lampropeltis triangulum* | -98.95 | 21.98 |
| *Lampropeltis triangulum* | -105.62 | 22.51 |
| *Lampropeltis triangulum* | -105.74 | 22.55 |
| *Lampropeltis triangulum* | -105.6 | 22.62 |
| *Lampropeltis triangulum* | -105.731942 | 22.652962 |
| *Lampropeltis triangulum* | -105.77 | 22.66 |
| *Lampropeltis triangulum* | -99.01 | 22.79 |
| *Lampropeltis triangulum* | -98.464 | 22.827 |
| *Lampropeltis triangulum* | -105.786231 | 22.838691 |
| *Lampropeltis triangulum* | -105.95 | 22.97 |
| *Lampropeltis triangulum* | -105.86 | 23 |
| *Lampropeltis triangulum* | -105.89 | 23.01 |
| *Lampropeltis triangulum* | -105.75 | 23.03 |
| *Lampropeltis triangulum* | -105.75 | 23.030001 |
| *Lampropeltis triangulum* | -98.769 | 23.043 |
| *Lampropeltis triangulum* | -105.47 | 23.07 |
| *Lampropeltis triangulum* | -106.09 | 23.16 |
| *Lampropeltis triangulum* | -106.4 | 23.23333 |
| *Lampropeltis triangulum* | -99.057216 | 23.236031 |
| *Lampropeltis triangulum* | -99.024 | 23.274 |
| *Lampropeltis triangulum* | -106.419998 | 23.33 |
| *Lampropeltis triangulum* | -106.379997 | 23.370001 |
| *Lampropeltis triangulum* | -106.51 | 23.39 |
| *Lampropeltis triangulum* | -106.46 | 23.43 |
| *Lampropeltis triangulum* | -106.46967 | 23.45213 |
| *Lampropeltis triangulum* | -106.476493 | 23.486247 |
| *Lampropeltis triangulum* | -106.509405 | 23.536017 |
| *Lampropeltis triangulum* | -106.44 | 23.55 |
| *Lampropeltis triangulum* | -106.54 | 23.59 |
| *Lampropeltis triangulum* | -106.56 | 23.61 |
| *Lampropeltis triangulum* | -106.74 | 23.65 |
| *Lampropeltis triangulum* | -106.51 | 23.85 |
| *Lampropeltis triangulum* | -106.91 | 23.91 |
| *Lampropeltis triangulum* | -106.42 | 23.94 |
| *Lampropeltis triangulum* | -99.109 | 23.95 |
| *Lampropeltis triangulum* | -106.71 | 23.96 |
| *Lampropeltis triangulum* | -106.98 | 23.99 |
| *Lampropeltis triangulum* | -98.82 | 24 |
| *Lampropeltis triangulum* | -107.03 | 24.08 |
| *Lampropeltis triangulum* | -106.89 | 24.12 |
| *Lampropeltis triangulum* | -107.07 | 24.13 |
| *Lampropeltis triangulum* | -106.750328 | 24.210268 |
| *Lampropeltis triangulum* | -107.06 | 24.23 |
| *Lampropeltis triangulum* | -107.37 | 24.35 |
| *Lampropeltis triangulum* | -107.09 | 24.43 |
| *Lampropeltis triangulum* | -107.19 | 24.55 |
| *Lampropeltis triangulum* | -107.216711 | 24.583403 |
| *Lampropeltis triangulum* | -98.912 | 24.621 |
| *Lampropeltis triangulum* | -99.543 | 24.626 |
| *Lampropeltis triangulum* | -107.27 | 24.64 |
| *Lampropeltis triangulum* | -107.44 | 24.66 |
| *Lampropeltis triangulum* | -106.91 | 24.67 |
| *Lampropeltis triangulum* | -107.400015 | 24.758179 |
| *Lampropeltis triangulum* | -107.36 | 24.76 |
| *Lampropeltis triangulum* | -107.44 | 24.85 |
| *Lampropeltis triangulum* | -107.35 | 24.86 |
| *Lampropeltis triangulum* | -107.39 | 24.94 |
| *Lampropeltis triangulum* | -97.978 | 24.993 |
| *Lampropeltis triangulum* | -98.07 | 25.05 |
| *Lampropeltis triangulum* | -98.116 | 25.099 |
| *Lampropeltis triangulum* | -97.812 | 25.144 |
| *Lampropeltis triangulum* | -98.112 | 25.147 |
| *Lampropeltis triangulum* | -97.940002 | 25.190001 |
| *Lampropeltis triangulum* | -97.91 | 25.22 |
| *Lampropeltis triangulum* | -108.09 | 25.27 |
| *Lampropeltis triangulum* | -108.03 | 25.29 |
| *Lampropeltis triangulum* | -103.33 | 25.36 |
| *Lampropeltis triangulum* | -107.53 | 25.37 |
| *Lampropeltis triangulum* | -107.49 | 25.39 |
| *Lampropeltis triangulum* | -107.63 | 25.42 |
| *Lampropeltis triangulum* | -108.01 | 25.46 |
| *Lampropeltis triangulum* | -107.45 | 25.52 |
| *Lampropeltis triangulum* | -80.632555 | 25.549175 |
| *Lampropeltis triangulum* | -107.27 | 25.59 |
| *Lampropeltis triangulum* | -107.38 | 25.66 |
| *Lampropeltis triangulum* | -80.479 | 25.686 |
| *Lampropeltis triangulum* | -80.36435 | 25.70101 |
| *Lampropeltis triangulum* | -80.267613 | 25.72256 |
| *Lampropeltis triangulum* | -97.506 | 25.862 |
| *Lampropeltis triangulum* | -97.5 | 25.93 |
| *Lampropeltis triangulum* | -81.3111 | 25.9736 |
| *Lampropeltis triangulum* | -80.365725 | 26.189327 |
| *Lampropeltis triangulum* | -107.71 | 26.33 |
| *Lampropeltis triangulum* | -81.779 | 26.3405 |
| *Lampropeltis triangulum* | -80.082991 | 26.358698 |
| *Lampropeltis triangulum* | -108.668514 | 26.39871 |
| *Lampropeltis triangulum* | -80.068 | 26.714 |
| *Lampropeltis triangulum* | -108.69417 | 26.90278 |
| *Lampropeltis triangulum* | -109.040566 | 27.067022 |
| *Lampropeltis triangulum* | -97.789 | 27.11933 |
| *Lampropeltis triangulum* | -97.789 | 27.11933 |
| *Lampropeltis triangulum* | -98.005794 | 27.227906 |
| *Lampropeltis triangulum* | -98.652946 | 27.240973 |
| *Lampropeltis triangulum* | -97.968465 | 27.245743 |
| *Lampropeltis triangulum* | -97.984175 | 27.2963 |
| *Lampropeltis triangulum* | -98.673186 | 27.3538 |
| *Lampropeltis triangulum* | -98.99228 | 27.471243 |
| *Lampropeltis triangulum* | -80.543585 | 27.695373 |
| *Lampropeltis triangulum* | -98.62143 | 27.7634 |
| *Lampropeltis triangulum* | -82.3936 | 28.0768 |
| *Lampropeltis triangulum* | -81.62782 | 28.10273 |
| *Lampropeltis triangulum* | -80.630325 | 28.129061 |
| *Lampropeltis triangulum* | -96.762173 | 28.159425 |
| *Lampropeltis triangulum* | -101.55 | 28.19 |
| *Lampropeltis triangulum* | -98.288673 | 28.243362 |
| *Lampropeltis triangulum* | -98.569874 | 28.30112 |
| *Lampropeltis triangulum* | -81.4036 | 28.3261 |
| *Lampropeltis triangulum* | -99.08133 | 28.33267 |
| *Lampropeltis triangulum* | -81.58447 | 28.38173 |
| *Lampropeltis triangulum* | -81.379272 | 28.538339 |
| *Lampropeltis triangulum* | -98.34516 | 28.59074 |
| *Lampropeltis triangulum* | -82.36467 | 28.62702 |
| *Lampropeltis triangulum* | -82.33772 | 28.69115 |
| *Lampropeltis triangulum* | -81.18735 | 28.69232 |
| *Lampropeltis triangulum* | -82.297 | 28.75 |
| *Lampropeltis triangulum* | -100.516319 | 28.78618 |
| *Lampropeltis triangulum* | -81.96702 | 28.99914 |
| *Lampropeltis triangulum* | -82.5249 | 29.00181 |
| *Lampropeltis triangulum* | -81.076484 | 29.032302 |
| *Lampropeltis triangulum* | -102.952106 | 29.177428 |
| *Lampropeltis triangulum* | -82.14022 | 29.18724 |
| *Lampropeltis triangulum* | -82.0578 | 29.2164 |
| *Lampropeltis triangulum* | -82.0011 | 29.2906 |
| *Lampropeltis triangulum* | -103.93512 | 29.29492 |
| *Lampropeltis triangulum* | -98.592918 | 29.40401 |
| *Lampropeltis triangulum* | -103.51662 | 29.45996 |
| *Lampropeltis triangulum* | -82.10037 | 29.51164 |
| *Lampropeltis triangulum* | -82.253366 | 29.565505 |
| *Lampropeltis triangulum* | -103.13965 | 29.5858 |
| *Lampropeltis triangulum* | -82.32448 | 29.6514 |
| *Lampropeltis triangulum* | -85.311 | 29.694 |
| *Lampropeltis triangulum* | -82.2267 | 29.6858 |
| *Lampropeltis triangulum* | -85.27635 | 29.69764 |
| *Lampropeltis triangulum* | -93.593 | 29.783 |
| *Lampropeltis triangulum* | -101.200561 | 29.767025 |
| *Lampropeltis triangulum* | -90.445869 | 29.779136 |
| *Lampropeltis triangulum* | -83.316208 | 29.815153 |
| *Lampropeltis triangulum* | -101.167667 | 29.830479 |
| *Lampropeltis triangulum* | -90.1421 | 29.9055 |
| *Lampropeltis triangulum* | -91.096929 | 29.921764 |
| *Lampropeltis triangulum* | -101.136208 | 29.953991 |
| *Lampropeltis triangulum* | -90.46083 | 29.961181 |
| *Lampropeltis triangulum* | -94.852633 | 29.969327 |
| *Lampropeltis triangulum* | -90.486638 | 30.059516 |
| *Lampropeltis triangulum* | -85.15822 | 30.13031 |
| *Lampropeltis triangulum* | -94.11205 | 30.22127 |
| *Lampropeltis triangulum* | -99.87 | 30.25 |
| *Lampropeltis triangulum* | -102.44064 | 30.28294 |
| *Lampropeltis triangulum* | -91.405452 | 30.28834 |
| *Lampropeltis triangulum* | -91.01537 | 30.293592 |
| *Lampropeltis triangulum* | -99.1237 | 30.29958 |
| *Lampropeltis triangulum* | -90.977625 | 30.308434 |
| *Lampropeltis triangulum* | -81.608112 | 30.359374 |
| *Lampropeltis triangulum* | -89.92285 | 30.426256 |
| *Lampropeltis triangulum* | -84.280722 | 30.438219 |
| *Lampropeltis triangulum* | -96.256569 | 30.483978 |
| *Lampropeltis triangulum* | -90.957709 | 30.486824 |
| *Lampropeltis triangulum* | -95.688733 | 30.508107 |
| *Lampropeltis triangulum* | -87.004 | 30.594 |
| *Lampropeltis triangulum* | -86.64765 | 30.575155 |
| *Lampropeltis triangulum* | -96.209225 | 30.657398 |
| *Lampropeltis triangulum* | -91.997645 | 30.660689 |
| *Lampropeltis triangulum* | -103.79382 | 30.66751 |
| *Lampropeltis triangulum* | -88.045578 | 30.696441 |
| *Lampropeltis triangulum* | -84.13975 | 30.72696 |
| *Lampropeltis triangulum* | -88.84428 | 30.72729 |
| *Lampropeltis triangulum* | -81.555817 | 30.743336 |
| *Lampropeltis triangulum* | -81.55582 | 30.74334 |
| *Lampropeltis triangulum* | -90.82298 | 30.79785 |
| *Lampropeltis triangulum* | -91.020592 | 30.845793 |
| *Lampropeltis triangulum* | -81.454336 | 30.854156 |
| *Lampropeltis triangulum* | -101.06017 | 30.99694 |
| *Lampropeltis triangulum* | -93.276897 | 31.11257 |
| *Lampropeltis triangulum* | -82.126191 | 31.117151 |
| *Lampropeltis triangulum* | -93.91864 | 31.21916 |
| *Lampropeltis triangulum* | -81.60249 | 31.260122 |
| *Lampropeltis triangulum* | -81.903297 | 31.330665 |
| *Lampropeltis triangulum* | -109.156443 | 31.344968 |
| *Lampropeltis triangulum* | -82.196049 | 31.359947 |
| *Lampropeltis triangulum* | -100.439464 | 31.383001 |
| *Lampropeltis triangulum* | -108.9 | 31.45 |
| *Lampropeltis triangulum* | -81.253693 | 31.457132 |
| *Lampropeltis triangulum* | -81.55118 | 31.574042 |
| *Lampropeltis triangulum* | -82.1101 | 31.6586 |
| *Lampropeltis triangulum* | -81.441403 | 31.744597 |
| *Lampropeltis triangulum* | -93.08756 | 31.75382 |
| *Lampropeltis triangulum* | -106.36741 | 31.79348 |
| *Lampropeltis triangulum* | -82.755536 | 31.984486 |
| *Lampropeltis triangulum* | -81.242268 | 31.99892 |
| *Lampropeltis triangulum* | -81.343 | 32.053 |
| *Lampropeltis triangulum* | -81.2797 | 32.0539 |
| *Lampropeltis triangulum* | -81.0965 | 32.0823 |
| *Lampropeltis triangulum* | -82.617394 | 32.095195 |
| *Lampropeltis triangulum* | -82.126685 | 32.096864 |
| *Lampropeltis triangulum* | -83.752941 | 32.154193 |
| *Lampropeltis triangulum* | -81.17875 | 32.15548 |
| *Lampropeltis triangulum* | -95.8553 | 32.2047 |
| *Lampropeltis triangulum* | -81.182379 | 32.26846 |
| *Lampropeltis triangulum* | -103.18 | 32.28 |
| *Lampropeltis triangulum* | -82.40709 | 32.320127 |
| *Lampropeltis triangulum* | -103.8 | 32.35 |
| *Lampropeltis triangulum* | -92.12092 | 32.35362 |
| *Lampropeltis triangulum* | -103.78 | 32.37 |
| *Lampropeltis triangulum* | -81.03241 | 32.37822 |
| *Lampropeltis triangulum* | -103.12 | 32.44 |
| *Lampropeltis triangulum* | -81.1811 | 32.4633 |
| *Lampropeltis triangulum* | -80.9245 | 32.468498 |
| *Lampropeltis triangulum* | -80.9245 | 32.4685 |
| *Lampropeltis triangulum* | -82.299395 | 32.511263 |
| *Lampropeltis triangulum* | -94.69138 | 32.54288 |
| *Lampropeltis triangulum* | -107.49 | 32.59 |
| *Lampropeltis triangulum* | -92.06733 | 32.59832 |
| *Lampropeltis triangulum* | -93.28664 | 32.61549 |
| *Lampropeltis triangulum* | -93.551164 | 32.750721 |
| *Lampropeltis triangulum* | -96.75412 | 32.80082 |
| *Lampropeltis triangulum* | -81.109539 | 32.870965 |
| *Lampropeltis triangulum* | -79.8472 | 32.9486 |
| *Lampropeltis triangulum* | -104.17 | 33.05 |
| *Lampropeltis triangulum* | -87.125 | 33.11806 |
| *Lampropeltis triangulum* | -87.56917 | 33.20972 |
| *Lampropeltis triangulum* | -104 | 33.27 |
| *Lampropeltis triangulum* | -87.08556 | 33.27833 |
| *Lampropeltis triangulum* | -103.91 | 33.32 |
| *Lampropeltis triangulum* | -103.79 | 33.45 |
| *Lampropeltis triangulum* | -86.83434 | 33.46805 |
| *Lampropeltis triangulum* | -104.4 | 33.6 |
| *Lampropeltis triangulum* | -94.975962 | 33.604725 |
| *Lampropeltis triangulum* | -85.518718 | 33.621213 |
| *Lampropeltis triangulum* | -103.164884 | 33.642658 |
| *Lampropeltis triangulum* | -86.00536 | 33.65972 |
| *Lampropeltis triangulum* | -97.295 | 33.8799 |
| *Lampropeltis triangulum* | -96.83658 | 33.91184 |
| *Lampropeltis triangulum* | -103.37 | 33.94 |
| *Lampropeltis triangulum* | -83.3768 | 33.9574 |
| *Lampropeltis triangulum* | -80.83423 | 34.04135 |
| *Lampropeltis triangulum* | -84.83 | 34.15 |
| *Lampropeltis triangulum* | -95.90197 | 34.3265 |
| *Lampropeltis triangulum* | -95.861766 | 34.329713 |
| *Lampropeltis triangulum* | -97.18019 | 34.33255 |
| *Lampropeltis triangulum* | -105.69 | 34.37 |
| *Lampropeltis triangulum* | -95.82706 | 34.40764 |
| *Lampropeltis triangulum* | -93.92338 | 34.4133 |
| *Lampropeltis triangulum* | -105.8 | 34.43 |
| *Lampropeltis triangulum* | -93.97561 | 34.43488 |
| *Lampropeltis triangulum* | -94.01965 | 34.44051 |
| *Lampropeltis triangulum* | -95.21555 | 34.4823 |
| *Lampropeltis triangulum* | -96.01747 | 34.51389 |
| *Lampropeltis triangulum* | -85.684566 | 34.521088 |
| *Lampropeltis triangulum* | -95.34362 | 34.52597 |
| *Lampropeltis triangulum* | -79.2914 | 34.5366 |
| *Lampropeltis triangulum* | -95.94116 | 34.54244 |
| *Lampropeltis triangulum* | -95.07645 | 34.54564 |
| *Lampropeltis triangulum* | -95.963766 | 34.55442 |
| *Lampropeltis triangulum* | -92.591019 | 34.556064 |
| *Lampropeltis triangulum* | -87.301514 | 34.556753 |
| *Lampropeltis triangulum* | -106.01 | 34.56 |
| *Lampropeltis triangulum* | -105.147439 | 34.58219 |
| *Lampropeltis triangulum* | -94.49722 | 34.61528 |
| *Lampropeltis triangulum* | -106.34 | 34.62 |
| *Lampropeltis triangulum* | -95.38711 | 34.62645 |
| *Lampropeltis triangulum* | -94.5625 | 34.6868 |
| *Lampropeltis triangulum* | -94.53077 | 34.69239 |
| *Lampropeltis triangulum* | -87.7922 | 34.7294 |
| *Lampropeltis triangulum* | -109.876679 | 34.808981 |
| *Lampropeltis triangulum* | -106.22 | 34.82 |
| *Lampropeltis triangulum* | -94.617388 | 34.86779 |
| *Lampropeltis triangulum* | -106.05 | 34.87 |
| *Lampropeltis triangulum* | -95.35556 | 34.98713 |
| *Lampropeltis triangulum* | -106.3 | 35.08 |
| *Lampropeltis triangulum* | -106.3 | 35.03 |
| *Lampropeltis triangulum* | -106.39 | 35.08 |
| *Lampropeltis triangulum* | -82.77315 | 35.214441 |
| *Lampropeltis triangulum* | -111.069158 | 35.223028 |
| *Lampropeltis triangulum* | -82.75 | 35.25 |
| *Lampropeltis triangulum* | -94.53345 | 35.54864 |
| *Lampropeltis triangulum* | -95.96321 | 35.62516 |
| *Lampropeltis triangulum* | -94.75671 | 35.64429 |
| *Lampropeltis triangulum* | -94.87205 | 35.70505 |
| *Lampropeltis triangulum* | -94.86723 | 35.76529 |
| *Lampropeltis triangulum* | -83.13611 | 35.78333 |
| *Lampropeltis triangulum* | -78.564536 | 35.797215 |
| *Lampropeltis triangulum* | -96.322493 | 35.835593 |
| *Lampropeltis triangulum* | -105.06 | 35.84 |
| *Lampropeltis triangulum* | -82.95889 | 35.84889 |
| *Lampropeltis triangulum* | -75.85834 | 35.86358 |
| *Lampropeltis triangulum* | -92.118251 | 35.866158 |
| *Lampropeltis triangulum* | -95.841637 | 35.949297 |
| *Lampropeltis triangulum* | -94.803108 | 35.971489 |
| *Lampropeltis triangulum* | -81.909796 | 36.11981 |
| *Lampropeltis triangulum* | -102.65 | 36.14 |
| *Lampropeltis triangulum* | -86.78 | 36.18 |
| *Lampropeltis triangulum* | -103.18 | 36.29 |
| *Lampropeltis triangulum* | -107.1 | 36.3 |
| *Lampropeltis triangulum* | -95.02634 | 36.39509 |
| *Lampropeltis triangulum* | -105.05 | 36.57 |
| *Lampropeltis triangulum* | -78.3433 | 36.5932 |
| *Lampropeltis triangulum* | -93.718871 | 36.610142 |
| *Lampropeltis triangulum* | -81.6 | 36.63 |
| *Lampropeltis triangulum* | -89.12773 | 36.66917 |
| *Lampropeltis triangulum* | -82.40304 | 36.67244 |
| *Lampropeltis triangulum* | -81.6118 | 36.6744 |
| *Lampropeltis triangulum* | -83.0758 | 36.686 |
| *Lampropeltis triangulum* | -76.511 | 36.71888 |
| *Lampropeltis triangulum* | -77.1017 | 36.7533 |
| *Lampropeltis triangulum* | -77.2819 | 36.7912 |
| *Lampropeltis triangulum* | -104.7 | 36.8 |
| *Lampropeltis triangulum* | -95.85886 | 36.80049 |
| *Lampropeltis triangulum* | -82.2408 | 36.8265 |
| *Lampropeltis triangulum* | -88.07472 | 36.85694 |
| *Lampropeltis triangulum* | -81.916 | 36.896 |
| *Lampropeltis triangulum* | -82.0677 | 36.8968 |
| *Lampropeltis triangulum* | -77.0245 | 36.9121 |
| *Lampropeltis triangulum* | -80.26311 | 36.91212 |
| *Lampropeltis triangulum* | -83.207764 | 36.948702 |
| *Lampropeltis triangulum* | -97.62144 | 36.97189 |
| *Lampropeltis triangulum* | -97.61073 | 36.98898 |
| *Lampropeltis triangulum* | -99.31973 | 37.01305 |
| *Lampropeltis triangulum* | -94.6224 | 37.0259 |
| *Lampropeltis triangulum* | -96.08482 | 37.04622 |
| *Lampropeltis triangulum* | -105.7444 | 37.09652 |
| *Lampropeltis triangulum* | -82.869867 | 37.122337 |
| *Lampropeltis triangulum* | -100.77979 | 37.13217 |
| *Lampropeltis triangulum* | -100.46944 | 37.16972 |
| *Lampropeltis triangulum* | -98.69945 | 37.21833 |
| *Lampropeltis triangulum* | -98.699448 | 37.218334 |
| *Lampropeltis triangulum* | -108.464583 | 37.234755 |
| *Lampropeltis triangulum* | -101.564697 | 37.263199 |
| *Lampropeltis triangulum* | -101.5647 | 37.2632 |
| *Lampropeltis triangulum* | -97.06612 | 37.3018 |
| *Lampropeltis triangulum* | -76.96 | 37.31 |
| *Lampropeltis triangulum* | -88.9135 | 37.34362 |
| *Lampropeltis triangulum* | -95.96309 | 37.37985 |
| *Lampropeltis triangulum* | -94.705 | 37.41111 |
| *Lampropeltis triangulum* | -101.15996 | 37.43129 |
| *Lampropeltis triangulum* | -101.2621 | 37.438499 |
| *Lampropeltis triangulum* | -101.2621 | 37.4385 |
| *Lampropeltis triangulum* | -79.7 | 37.45 |
| *Lampropeltis triangulum* | -79.5 | 37.46 |
| *Lampropeltis triangulum* | -105.991893 | 37.516885 |
| *Lampropeltis triangulum* | -102.01199 | 37.52364 |
| *Lampropeltis triangulum* | -95.811432 | 37.526661 |
| *Lampropeltis triangulum* | -95.216171 | 37.554192 |
| *Lampropeltis triangulum* | -95.33179 | 37.55826 |
| *Lampropeltis triangulum* | -101.742814 | 37.571254 |
| *Lampropeltis triangulum* | -75.77358 | 37.60342 |
| *Lampropeltis triangulum* | -79.33 | 37.62 |
| *Lampropeltis triangulum* | -94.703333 | 37.635556 |
| *Lampropeltis triangulum* | -94.70333 | 37.63556 |
| *Lampropeltis triangulum* | -79.32147 | 37.69247 |
| *Lampropeltis triangulum* | -79.91 | 37.72 |
| *Lampropeltis triangulum* | -80.91168 | 37.73773 |
| *Lampropeltis triangulum* | -76.5136 | 37.7776 |
| *Lampropeltis triangulum* | -95.07816 | 37.78594 |
| *Lampropeltis triangulum* | -95.8394 | 37.787 |
| *Lampropeltis triangulum* | -78.75542 | 37.79088 |
| *Lampropeltis triangulum* | -94.77801 | 37.79354 |
| *Lampropeltis triangulum* | -79.25609 | 37.79449 |
| *Lampropeltis triangulum* | -100.29389 | 37.80028 |
| *Lampropeltis triangulum* | -96.54667 | 37.80972 |
| *Lampropeltis triangulum* | -94.81503 | 37.8126 |
| *Lampropeltis triangulum* | -101.0506 | 37.8219 |
| *Lampropeltis triangulum* | -94.82444 | 37.83484 |
| *Lampropeltis triangulum* | -83.66347 | 37.8357 |
| *Lampropeltis triangulum* | -94.70333 | 37.83667 |
| *Lampropeltis triangulum* | -78.61 | 37.88 |
| *Lampropeltis triangulum* | -103.099053 | 37.893745 |
| *Lampropeltis triangulum* | -79.06 | 37.91 |
| *Lampropeltis triangulum* | -80.21106 | 37.91647 |
| *Lampropeltis triangulum* | -87.89397 | 37.93655 |
| *Lampropeltis triangulum* | -78.93422 | 37.94058 |
| *Lampropeltis triangulum* | -94.97 | 37.94265 |
| *Lampropeltis triangulum* | -94.7132 | 37.95718 |
| *Lampropeltis triangulum* | -89.8866 | 37.96486 |
| *Lampropeltis triangulum* | -105.901246 | 37.994057 |
| *Lampropeltis triangulum* | -79.79 | 38.01 |
| *Lampropeltis triangulum* | -94.98799 | 38.01006 |
| *Lampropeltis triangulum* | -78.47 | 38.02 |
| *Lampropeltis triangulum* | -101.9915 | 38.03557 |
| *Lampropeltis triangulum* | -99.8885 | 38.0512 |
| *Lampropeltis triangulum* | -96.03779 | 38.05381 |
| *Lampropeltis triangulum* | -78.8 | 38.07 |
| *Lampropeltis triangulum* | -79.46 | 38.11 |
| *Lampropeltis triangulum* | -87.931488 | 38.128264 |
| *Lampropeltis triangulum* | -94.91505 | 38.14834 |
| *Lampropeltis triangulum* | -79.29 | 38.16 |
| *Lampropeltis triangulum* | -104.175056 | 38.178688 |
| *Lampropeltis triangulum* | -104.17506 | 38.17869 |
| *Lampropeltis triangulum* | -95.2975 | 38.18361 |
| *Lampropeltis triangulum* | -94.78127 | 38.19353 |
| *Lampropeltis triangulum* | -95.23998 | 38.19637 |
| *Lampropeltis triangulum* | -79.32792 | 38.22938 |
| *Lampropeltis triangulum* | -79.32792 | 38.22938 |
| *Lampropeltis triangulum* | -79.12 | 38.23 |
| *Lampropeltis triangulum* | -78.67065 | 38.25399 |
| *Lampropeltis triangulum* | -79.32 | 38.26 |
| *Lampropeltis triangulum* | -95.14826 | 38.2607 |
| *Lampropeltis triangulum* | -78.65923 | 38.28826 |
| *Lampropeltis triangulum* | -99.74516 | 38.29072 |
| *Lampropeltis triangulum* | -90.30168 | 38.30024 |
| *Lampropeltis triangulum* | -79.17 | 38.31 |
| *Lampropeltis triangulum* | -95.857498 | 38.339169 |
| *Lampropeltis triangulum* | -95.8575 | 38.33917 |
| *Lampropeltis triangulum* | -79.33 | 38.34 |
| *Lampropeltis triangulum* | -79.5898 | 38.3465 |
| *Lampropeltis triangulum* | -95.33271 | 38.3526 |
| *Lampropeltis triangulum* | -78.54432 | 38.35424 |
| *Lampropeltis triangulum* | -90.349558 | 38.356781 |
| *Lampropeltis triangulum* | -79.51004 | 38.3645 |
| *Lampropeltis triangulum* | -79.5088 | 38.3646 |
| *Lampropeltis triangulum* | -78.4275 | 38.3662 |
| *Lampropeltis triangulum* | -96.59364 | 38.3717 |
| *Lampropeltis triangulum* | -96.80152 | 38.39354 |
| *Lampropeltis triangulum* | -96.74932 | 38.40968 |
| *Lampropeltis triangulum* | -99.69604 | 38.4111 |
| *Lampropeltis triangulum* | -79.30051 | 38.41454 |
| *Lampropeltis triangulum* | -95.08736 | 38.41782 |
| *Lampropeltis triangulum* | -94.795 | 38.41861 |
| *Lampropeltis triangulum* | -79.59909 | 38.42846 |
| *Lampropeltis triangulum* | -79.27 | 38.43 |
| *Lampropeltis triangulum* | -79.65 | 38.4338 |
| *Lampropeltis triangulum* | -99.62247 | 38.45298 |
| *Lampropeltis triangulum* | -79.24 | 38.46 |
| *Lampropeltis triangulum* | -85.303568 | 38.462288 |
| *Lampropeltis triangulum* | -95.0912 | 38.47 |
| *Lampropeltis triangulum* | -95.598587 | 38.476372 |
| *Lampropeltis triangulum* | -78.45636 | 38.47839 |
| *Lampropeltis triangulum* | -94.76139 | 38.49138 |
| *Lampropeltis triangulum* | -78.4206 | 38.4915 |
| *Lampropeltis triangulum* | -98.58889 | 38.50017 |
| *Lampropeltis triangulum* | -94.647 | 38.507 |
| *Lampropeltis triangulum* | -95.61 | 38.50694 |
| *Lampropeltis triangulum* | -95.637497 | 38.506943 |
| *Lampropeltis triangulum* | -78.4253 | 38.5285 |
| *Lampropeltis triangulum* | -95.08381 | 38.5318 |
| *Lampropeltis triangulum* | -78.4147 | 38.5457 |
| *Lampropeltis triangulum* | -78.4149 | 38.5462 |
| *Lampropeltis triangulum* | -78.395 | 38.5554 |
| *Lampropeltis triangulum* | -94.184113 | 38.560815 |
| *Lampropeltis triangulum* | -94.837433 | 38.569416 |
| *Lampropeltis triangulum* | -94.83722 | 38.56944 |
| *Lampropeltis triangulum* | -95.278549 | 38.57111 |
| *Lampropeltis triangulum* | -78.41061 | 38.57817 |
| *Lampropeltis triangulum* | -78.3674 | 38.5854 |
| *Lampropeltis triangulum* | -79.05972 | 38.58923 |
| *Lampropeltis triangulum* | -95.05608 | 38.63641 |
| *Lampropeltis triangulum* | -98.5419 | 38.6461 |
| *Lampropeltis triangulum* | -99.326 | 38.6543 |
| *Lampropeltis triangulum* | -79.51412 | 38.65445 |
| *Lampropeltis triangulum* | -78.4595 | 38.6654 |
| *Lampropeltis triangulum* | -79.07778 | 38.66709 |
| *Lampropeltis triangulum* | -83.24467 | 38.66777 |
| *Lampropeltis triangulum* | -104.86167 | 38.67014 |
| *Lampropeltis triangulum* | -97.99223 | 38.67722 |
| *Lampropeltis triangulum* | -99.2597 | 38.6896 |
| *Lampropeltis triangulum* | -99.36106 | 38.69069 |
| *Lampropeltis triangulum* | -99.156 | 38.6972 |
| *Lampropeltis triangulum* | -87.390216 | 38.697858 |
| *Lampropeltis triangulum* | -97.7988 | 38.7164 |
| *Lampropeltis triangulum* | -99.1449 | 38.7167 |
| *Lampropeltis triangulum* | -99.3222 | 38.7279 |
| *Lampropeltis triangulum* | -78.31834 | 38.7313 |
| *Lampropeltis triangulum* | -86.47169 | 38.734171 |
| *Lampropeltis triangulum* | -99.3545 | 38.7395 |
| *Lampropeltis triangulum* | -99.5477 | 38.748 |
| *Lampropeltis triangulum* | -93.60659 | 38.749398 |
| *Lampropeltis triangulum* | -108.085427 | 38.74961 |
| *Lampropeltis triangulum* | -77.47 | 38.75 |
| *Lampropeltis triangulum* | -92.199013 | 38.752662 |
| *Lampropeltis triangulum* | -83.86097 | 38.75877 |
| *Lampropeltis triangulum* | -85.41861 | 38.75944 |
| *Lampropeltis triangulum* | -79.78188 | 38.76155 |
| *Lampropeltis triangulum* | -101.3064 | 38.7625 |
| *Lampropeltis triangulum* | -92.480451 | 38.764925 |
| *Lampropeltis triangulum* | -83.1713 | 38.77278 |
| *Lampropeltis triangulum* | -78.5386 | 38.774 |
| *Lampropeltis triangulum* | -99.7883 | 38.777 |
| *Lampropeltis triangulum* | -98.81985 | 38.78238 |
| *Lampropeltis triangulum* | -78.19789 | 38.78408 |
| *Lampropeltis triangulum* | -99.81611 | 38.79361 |
| *Lampropeltis triangulum* | -96.89306 | 38.7975 |
| *Lampropeltis triangulum* | -99.0196 | 38.7994 |
| *Lampropeltis triangulum* | -78.17786 | 38.8116 |
| *Lampropeltis triangulum* | -101.0142 | 38.8173 |
| *Lampropeltis triangulum* | -96.9003 | 38.8206 |
| *Lampropeltis triangulum* | -99.4088 | 38.8335 |
| *Lampropeltis triangulum* | -99.10405 | 38.83648 |
| *Lampropeltis triangulum* | -95.38028 | 38.84028 |
| *Lampropeltis triangulum* | -95.24401 | 38.84092 |
| *Lampropeltis triangulum* | -99.2965 | 38.8422 |
| *Lampropeltis triangulum* | -98.16039 | 38.84308 |
| *Lampropeltis triangulum* | -101.6026 | 38.8496 |
| *Lampropeltis triangulum* | -100.977 | 38.8527 |
| *Lampropeltis triangulum* | -99.4614 | 38.8571 |
| *Lampropeltis triangulum* | -78.20238 | 38.86619 |
| *Lampropeltis triangulum* | -78.68652 | 38.8662 |
| *Lampropeltis triangulum* | -99.41497 | 38.87196 |
| *Lampropeltis triangulum* | -98.7579 | 38.8738 |
| *Lampropeltis triangulum* | -95.4181 | 38.8766 |
| *Lampropeltis triangulum* | -98.68153 | 38.89323 |
| *Lampropeltis triangulum* | -94.81583 | 38.89611 |
| *Lampropeltis triangulum* | -96.85806 | 38.90055 |
| *Lampropeltis triangulum* | -95.3255 | 38.9057 |
| *Lampropeltis triangulum* | -99.4453 | 38.9058 |
| *Lampropeltis triangulum* | -99.6584 | 38.9066 |
| *Lampropeltis triangulum* | -95.01315 | 38.9142 |
| *Lampropeltis triangulum* | -95.4366 | 38.9202 |
| *Lampropeltis triangulum* | -95.45657 | 38.92057 |
| *Lampropeltis triangulum* | -95.23278 | 38.92111 |
| *Lampropeltis triangulum* | -79.84821 | 38.92575 |
| *Lampropeltis triangulum* | -98.7038 | 38.9287 |
| *Lampropeltis triangulum* | -100.49139 | 38.93111 |
| *Lampropeltis triangulum* | -98.798 | 38.9355 |
| *Lampropeltis triangulum* | -95.371944 | 38.935556 |
| *Lampropeltis triangulum* | -95.28041 | 38.93572 |
| *Lampropeltis triangulum* | -96.48944 | 38.93595 |
| *Lampropeltis triangulum* | -98.7517 | 38.9373 |
| *Lampropeltis triangulum* | -94.8081 | 38.93774 |
| *Lampropeltis triangulum* | -77.22 | 38.95 |
| *Lampropeltis triangulum* | -84.45833 | 38.95 |
| *Lampropeltis triangulum* | -99.0108 | 38.9513 |
| *Lampropeltis triangulum* | -94.90417 | 38.95139 |
| *Lampropeltis triangulum* | -98.8345 | 38.952 |
| *Lampropeltis triangulum* | -95.316391 | 38.97139 |
| *Lampropeltis triangulum* | -95.23473 | 38.97139 |
| *Lampropeltis triangulum* | -98.8757 | 38.9734 |
| *Lampropeltis triangulum* | -99.00193 | 38.97605 |
| *Lampropeltis triangulum* | -94.918 | 38.9784 |
| *Lampropeltis triangulum* | -98.7003 | 38.9818 |
| *Lampropeltis triangulum* | -96.51972 | 38.98444 |
| *Lampropeltis triangulum* | -78.361 | 38.9849 |
| *Lampropeltis triangulum* | -98.8246 | 38.9915 |
| *Lampropeltis triangulum* | -94.317887 | 39.005214 |
| *Lampropeltis triangulum* | -95.46778 | 39.00917 |
| *Lampropeltis triangulum* | -94.87991 | 39.01554 |
| *Lampropeltis triangulum* | -96.34639 | 39.0175 |
| *Lampropeltis triangulum* | -84.79553 | 39.02103 |
| *Lampropeltis triangulum* | -98.07278 | 39.02119 |
| *Lampropeltis triangulum* | -98.9264 | 39.0301 |
| *Lampropeltis triangulum* | -95.5325 | 39.03611 |
| *Lampropeltis triangulum* | -95.67778 | 39.04833 |
| *Lampropeltis triangulum* | -77.60433 | 39.04907 |
| *Lampropeltis triangulum* | -78.36 | 39.05 |
| *Lampropeltis triangulum* | -95.18791 | 39.0502 |
| *Lampropeltis triangulum* | -94.88194 | 39.06 |
| *Lampropeltis triangulum* | -94.881943 | 39.060001 |
| *Lampropeltis triangulum* | -99.3157 | 39.0637 |
| *Lampropeltis triangulum* | -96.82917 | 39.064167 |
| *Lampropeltis triangulum* | -99.2144 | 39.0675 |
| *Lampropeltis triangulum* | -93.194638 | 39.068026 |
| *Lampropeltis triangulum* | -99.4003 | 39.0686 |
| *Lampropeltis triangulum* | -98.11867 | 39.07021 |
| *Lampropeltis triangulum* | -95.21587 | 39.07287 |
| *Lampropeltis triangulum* | -95.34207 | 39.07365 |
| *Lampropeltis triangulum* | -108.552 | 39.07603 |
| *Lampropeltis triangulum* | -94.85083 | 39.07611 |
| *Lampropeltis triangulum* | -109.27623 | 39.07968 |
| *Lampropeltis triangulum* | -94.413882 | 39.090672 |
| *Lampropeltis triangulum* | -96.57667 | 39.09417 |
| *Lampropeltis triangulum* | -84.18962 | 39.09466 |
| *Lampropeltis triangulum* | -96.79611 | 39.09555 |
| *Lampropeltis triangulum* | -98.6968 | 39.102 |
| *Lampropeltis triangulum* | -76.5711 | 39.1073 |
| *Lampropeltis triangulum* | -84.49306 | 39.11667 |
| *Lampropeltis triangulum* | -99.2751 | 39.1169 |
| *Lampropeltis triangulum* | -96.28972 | 39.1175 |
| *Lampropeltis triangulum* | -95.18916 | 39.11902 |
| *Lampropeltis triangulum* | -95.47945 | 39.13861 |
| *Lampropeltis triangulum* | -99.185501 | 39.139999 |
| *Lampropeltis triangulum* | -99.1855 | 39.14 |
| *Lampropeltis triangulum* | -96.64361 | 39.15028 |
| *Lampropeltis triangulum* | -96.57694 | 39.15778 |
| *Lampropeltis triangulum* | -86.52759 | 39.16389 |
| *Lampropeltis triangulum* | -77.23686 | 39.16454 |
| *Lampropeltis triangulum* | -95.38625 | 39.16605 |
| *Lampropeltis triangulum* | -78.16 | 39.18 |
| *Lampropeltis triangulum* | -84.38649 | 39.18351 |
| *Lampropeltis triangulum* | -84.26806 | 39.21453 |
| *Lampropeltis triangulum* | -86.15814 | 39.24485 |
| *Lampropeltis triangulum* | -77.29223 | 39.25766 |
| *Lampropeltis triangulum* | -95.1075 | 39.261 |
| *Lampropeltis triangulum* | -97.86153 | 39.26395 |
| *Lampropeltis triangulum* | -96.09125 | 39.28929 |
| *Lampropeltis triangulum* | -96.58556 | 39.29147 |
| *Lampropeltis triangulum* | -94.9945 | 39.2956 |
| *Lampropeltis triangulum* | -95.62639 | 39.29583 |
| *Lampropeltis triangulum* | -98.19139 | 39.32111 |
| *Lampropeltis triangulum* | -82.35838 | 39.32947 |
| *Lampropeltis triangulum* | -99.837 | 39.343 |
| *Lampropeltis triangulum* | -74.482 | 39.373 |
| *Lampropeltis triangulum* | -82.77429 | 39.35807 |
| *Lampropeltis triangulum* | -100.22972 | 39.36333 |
| *Lampropeltis triangulum* | -99.90222 | 39.36417 |
| *Lampropeltis triangulum* | -99.27821 | 39.37635 |
| *Lampropeltis triangulum* | -79.74839 | 39.39176 |
| *Lampropeltis triangulum* | -99.46432 | 39.40599 |
| *Lampropeltis triangulum* | -96.165 | 39.43111 |
| *Lampropeltis triangulum* | -87.392119 | 39.445157 |
| *Lampropeltis triangulum* | -95.53111 | 39.44778 |
| *Lampropeltis triangulum* | -96.51961 | 39.45053 |
| *Lampropeltis triangulum* | -95.03965 | 39.45695 |
| *Lampropeltis triangulum* | -93.919561 | 39.462889 |
| *Lampropeltis triangulum* | -75.0261 | 39.4861 |
| *Lampropeltis triangulum* | -98.813202 | 39.487999 |
| *Lampropeltis triangulum* | -98.8132 | 39.488 |
| *Lampropeltis triangulum* | -96.0725 | 39.48972 |
| *Lampropeltis triangulum* | -84.702477 | 39.507996 |
| *Lampropeltis triangulum* | -97.863998 | 39.516998 |
| *Lampropeltis triangulum* | -110.52783 | 39.53383 |
| *Lampropeltis triangulum* | -79.48329 | 39.54119 |
| *Lampropeltis triangulum* | -96.53 | 39.5879 |
| *Lampropeltis triangulum* | -96.40778 | 39.58805 |
| *Lampropeltis triangulum* | -84.61945 | 39.58914 |
| *Lampropeltis triangulum* | -84.5275 | 39.59064 |
| *Lampropeltis triangulum* | -96.84202 | 39.59547 |
| *Lampropeltis triangulum* | -96.87304 | 39.6101 |
| *Lampropeltis triangulum* | -99.037903 | 39.6325 |
| *Lampropeltis triangulum* | -99.0379 | 39.6325 |
| *Lampropeltis triangulum* | -95.17038 | 39.6367 |
| *Lampropeltis triangulum* | -97.77861 | 39.63889 |
| *Lampropeltis triangulum* | -96.600594 | 39.638901 |
| *Lampropeltis triangulum* | -77.5728 | 39.6548 |
| *Lampropeltis triangulum* | -87.40442 | 39.65914 |
| *Lampropeltis triangulum* | -95.18339 | 39.66543 |
| *Lampropeltis triangulum* | -95.21171 | 39.66797 |
| *Lampropeltis triangulum* | -101.81017 | 39.67135 |
| *Lampropeltis triangulum* | -78.09639 | 39.68417 |
| *Lampropeltis triangulum* | -101.91288 | 39.68846 |
| *Lampropeltis triangulum* | -101.91288 | 39.688461 |
| *Lampropeltis triangulum* | -80.1184 | 39.69937 |
| *Lampropeltis triangulum* | -96.647301 | 39.7048 |
| *Lampropeltis triangulum* | -76.12885 | 39.7266 |
| *Lampropeltis triangulum* | -75.495 | 39.746 |
| *Lampropeltis triangulum* | -96.703598 | 39.733799 |
| *Lampropeltis triangulum* | -76.03929 | 39.73615 |
| *Lampropeltis triangulum* | -97.31852 | 39.73677 |
| *Lampropeltis triangulum* | -78.34459 | 39.73728 |
| *Lampropeltis triangulum* | -80.32199 | 39.78672 |
| *Lampropeltis triangulum* | -77.2 | 39.8 |
| *Lampropeltis triangulum* | -80.12022 | 39.81431 |
| *Lampropeltis triangulum* | -95.08823 | 39.8269 |
| *Lampropeltis triangulum* | -80.44639 | 39.83538 |
| *Lampropeltis triangulum* | -78.92282 | 39.84196 |
| *Lampropeltis triangulum* | -109.18507 | 39.84371 |
| *Lampropeltis triangulum* | -76.26748 | 39.84878 |
| *Lampropeltis triangulum* | -80.31705 | 39.8498 |
| *Lampropeltis triangulum* | -77.28954 | 39.852454 |
| *Lampropeltis triangulum* | -101.54056 | 39.85528 |
| *Lampropeltis triangulum* | -95.18122 | 39.85605 |
| *Lampropeltis triangulum* | -97.98111 | 39.86555 |
| *Lampropeltis triangulum* | -98.05635 | 39.86556 |
| *Lampropeltis triangulum* | -79.498 | 39.86818 |
| *Lampropeltis triangulum* | -97.9623 | 39.8728 |
| *Lampropeltis triangulum* | -79.46649 | 39.87854 |
| *Lampropeltis triangulum* | -79.07632 | 39.88757 |
| *Lampropeltis triangulum* | -76.69613 | 39.88882 |
| *Lampropeltis triangulum* | -78.65097 | 39.89267 |
| *Lampropeltis triangulum* | -80.18371 | 39.89885 |
| *Lampropeltis triangulum* | -75.630077 | 39.900037 |
| *Lampropeltis triangulum* | -74.3225 | 39.9025 |
| *Lampropeltis triangulum* | -80.21027 | 39.90417 |
| *Lampropeltis triangulum* | -84.62559 | 39.90514 |
| *Lampropeltis triangulum* | -78.72242 | 39.93713 |
| *Lampropeltis triangulum* | -74.7029 | 39.9393 |
| *Lampropeltis triangulum* | -102.02609 | 39.94155 |
| *Lampropeltis triangulum* | -75.293436 | 39.948839 |
| *Lampropeltis triangulum* | -74.3789 | 39.9544 |
| *Lampropeltis triangulum* | -75.364619 | 39.9807 |
| *Lampropeltis triangulum* | -101.7773 | 39.9807 |
| *Lampropeltis triangulum* | -75.0519 | 39.9814 |
| *Lampropeltis triangulum* | -97.35 | 40.00222 |
| *Lampropeltis triangulum* | -79.0728 | 40.0066 |
| *Lampropeltis triangulum* | -75.69999 | 40.00697 |
| *Lampropeltis triangulum* | -105.269639 | 40.011097 |
| *Lampropeltis triangulum* | -74.3117 | 40.0144 |
| *Lampropeltis triangulum* | -105.27 | 40.01497 |
| *Lampropeltis triangulum* | -97.435 | 40.01662 |
| *Lampropeltis triangulum* | -75.325552 | 40.034772 |
| *Lampropeltis triangulum* | -74.865013 | 40.071407 |
| *Lampropeltis triangulum* | -83.56594 | 40.08459 |
| *Lampropeltis triangulum* | -104.392429 | 40.087274 |
| *Lampropeltis triangulum* | -74.85271 | 40.10493 |
| *Lampropeltis triangulum* | -78.91579 | 40.11452 |
| *Lampropeltis triangulum* | -88.20384 | 40.11617 |
| *Lampropeltis triangulum* | -79.34048 | 40.1285 |
| *Lampropeltis triangulum* | -79.26476 | 40.13853 |
| *Lampropeltis triangulum* | -78.63371 | 40.14954 |
| *Lampropeltis triangulum* | -103.77885 | 40.1527 |
| *Lampropeltis triangulum* | -74.1258 | 40.1583 |
| *Lampropeltis triangulum* | -79.26311 | 40.16282 |
| *Lampropeltis triangulum* | -104.191289 | 40.165374 |
| *Lampropeltis triangulum* | -75.726908 | 40.170871 |
| *Lampropeltis triangulum* | -101.01278 | 40.17639 |
| *Lampropeltis triangulum* | -111.1295 | 40.18003 |
| *Lampropeltis triangulum* | -104.2114 | 40.21692 |
| *Lampropeltis triangulum* | -78.10239 | 40.23473 |
| *Lampropeltis triangulum* | -79.72914 | 40.24922 |
| *Lampropeltis triangulum* | -103.793223 | 40.265723 |
| *Lampropeltis triangulum* | -109.53337 | 40.28039 |
| *Lampropeltis triangulum* | -76.86507 | 40.28968 |
| *Lampropeltis triangulum* | -109.9889 | 40.29934 |
| *Lampropeltis triangulum* | -77.13054 | 40.2998 |
| *Lampropeltis triangulum* | -78.21862 | 40.32184 |
| *Lampropeltis triangulum* | -79.98672 | 40.32563 |
| *Lampropeltis triangulum* | -75.453498 | 40.331263 |
| *Lampropeltis triangulum* | -75.93793 | 40.33173 |
| *Lampropeltis triangulum* | -84.08233 | 40.33344 |
| *Lampropeltis triangulum* | -80.07651 | 40.34166 |
| *Lampropeltis triangulum* | -79.9286 | 40.35375 |
| *Lampropeltis triangulum* | -79.47076 | 40.36067 |
| *Lampropeltis triangulum* | -79.99569 | 40.36099 |
| *Lampropeltis triangulum* | -80.40099 | 40.36228 |
| *Lampropeltis triangulum* | -76.191622 | 40.365599 |
| *Lampropeltis triangulum* | -80.02499 | 40.37796 |
| *Lampropeltis triangulum* | -81.407081 | 40.394604 |
| *Lampropeltis triangulum* | -79.81922 | 40.39545 |
| *Lampropeltis triangulum* | -79.84118 | 40.3958 |
| *Lampropeltis triangulum* | -80.02816 | 40.40211 |
| *Lampropeltis triangulum* | -75.248011 | 40.410434 |
| *Lampropeltis triangulum* | -80.05225 | 40.41191 |
| *Lampropeltis triangulum* | -80.1619 | 40.42102 |
| *Lampropeltis triangulum* | -80.01141 | 40.4283 |
| *Lampropeltis triangulum* | -79.81627 | 40.42971 |
| *Lampropeltis triangulum* | -79.87943 | 40.4327 |
| *Lampropeltis triangulum* | -76.113758 | 40.435473 |
| *Lampropeltis triangulum* | -109.5285 | 40.44832 |
| *Lampropeltis triangulum* | -80.02225 | 40.45451 |
| *Lampropeltis triangulum* | -79.82333 | 40.47245 |
| *Lampropeltis triangulum* | -79.98929 | 40.47508 |
| *Lampropeltis triangulum* | -74.9703 | 40.4847 |
| *Lampropeltis triangulum* | -79.69729 | 40.49022 |
| *Lampropeltis triangulum* | -112.58175 | 40.49382 |
| *Lampropeltis triangulum* | -80.03603 | 40.5109 |
| *Lampropeltis triangulum* | -79.71912 | 40.52129 |
| *Lampropeltis triangulum* | -74.643349 | 40.5286 |
| *Lampropeltis triangulum* | -79.06507 | 40.53603 |
| *Lampropeltis triangulum* | -74.16183 | 40.56285 |
| *Lampropeltis triangulum* | -80.01476 | 40.57631 |
| *Lampropeltis triangulum* | -74.7708 | 40.6153 |
| *Lampropeltis triangulum* | -80.27107 | 40.62143 |
| *Lampropeltis triangulum* | -80.05494 | 40.62207 |
| *Lampropeltis triangulum* | -79.15599 | 40.62356 |
| *Lampropeltis triangulum* | -77.75018 | 40.65089 |
| *Lampropeltis triangulum* | -78.97533 | 40.65642 |
| *Lampropeltis triangulum* | -75.65617 | 40.65657 |
| *Lampropeltis triangulum* | -73.566197 | 40.657211 |
| *Lampropeltis triangulum* | -79.0556 | 40.6675 |
| *Lampropeltis triangulum* | -74.262941 | 40.692258 |
| *Lampropeltis triangulum* | -100.7978 | 40.6994 |
| *Lampropeltis triangulum* | -80.31095 | 40.701 |
| *Lampropeltis triangulum* | -73.844789 | 40.701232 |
| *Lampropeltis triangulum* | -73.841007 | 40.702443 |
| *Lampropeltis triangulum* | -79.36317 | 40.71225 |
| *Lampropeltis triangulum* | -73.794847 | 40.728317 |
| *Lampropeltis triangulum* | -77.98636 | 40.77738 |
| *Lampropeltis triangulum* | -77.49136 | 40.81322 |
| *Lampropeltis triangulum* | -74.040944 | 40.814584 |
| *Lampropeltis triangulum* | -77.57212 | 40.85399 |
| *Lampropeltis triangulum* | -82.32362 | 40.86096 |
| *Lampropeltis triangulum* | -79.91237 | 40.86136 |
| *Lampropeltis triangulum* | -79.9144 | 40.86407 |
| *Lampropeltis triangulum* | -73.45793 | 40.87164 |
| *Lampropeltis triangulum* | -76.55377 | 40.89932 |
| *Lampropeltis triangulum* | -75.11588 | 40.89992 |
| *Lampropeltis triangulum* | -96.15915 | 40.90512 |
| *Lampropeltis triangulum* | -73.13697 | 40.92355 |
| *Lampropeltis triangulum* | -74.078257 | 40.92588 |
| *Lampropeltis triangulum* | -73.12649 | 40.93931 |
| *Lampropeltis triangulum* | -78.34287 | 40.93938 |
| *Lampropeltis triangulum* | -80.06418 | 40.9448 |
| *Lampropeltis triangulum* | -103.34222 | 40.96255 |
| *Lampropeltis triangulum* | -76.41688 | 40.96415 |
| *Lampropeltis triangulum* | -72.608191 | 40.973101 |
| *Lampropeltis triangulum* | -78.96899 | 40.97557 |
| *Lampropeltis triangulum* | -77.25308 | 40.98142 |
| *Lampropeltis triangulum* | -77.13825 | 40.98566 |
| *Lampropeltis triangulum* | -76.05577 | 40.99118 |
| *Lampropeltis triangulum* | -78.11246 | 41.00078 |
| *Lampropeltis triangulum* | -73.6289 | 41.0264 |
| *Lampropeltis triangulum* | -73.69583 | 41.03833 |
| *Lampropeltis triangulum* | -77.30813 | 41.05031 |
| *Lampropeltis triangulum* | -75.21674 | 41.05215 |
| *Lampropeltis triangulum* | -80.08414 | 41.06944 |
| *Lampropeltis triangulum* | -78.24999 | 41.086 |
| *Lampropeltis triangulum* | -80.49651 | 41.09709 |
| *Lampropeltis triangulum* | -80.103 | 41.0997 |
| *Lampropeltis triangulum* | -80.10265 | 41.09981 |
| *Lampropeltis triangulum* | -72.36381 | 41.10409 |
| *Lampropeltis triangulum* | -81.74117 | 41.11583 |
| *Lampropeltis triangulum* | -80.18945 | 41.11792 |
| *Lampropeltis triangulum* | -73.79608 | 41.118187 |
| *Lampropeltis triangulum* | -76.51663 | 41.12502 |
| *Lampropeltis triangulum* | -73.274356 | 41.1344 |
| *Lampropeltis triangulum* | -75.1134 | 41.16645 |
| *Lampropeltis triangulum* | -79.1086 | 41.16843 |
| *Lampropeltis triangulum* | -75.09225 | 41.16965 |
| *Lampropeltis triangulum* | -76.81935 | 41.17094 |
| *Lampropeltis triangulum* | -76.5526 | 41.17102 |
| *Lampropeltis triangulum* | -73.2735 | 41.1779 |
| *Lampropeltis triangulum* | -79.45764 | 41.17929 |
| *Lampropeltis triangulum* | -76.39763 | 41.18008 |
| *Lampropeltis triangulum* | -78.50925 | 41.20018 |
| *Lampropeltis triangulum* | -78.85546 | 41.21341 |
| *Lampropeltis triangulum* | -79.02557 | 41.21433 |
| *Lampropeltis triangulum* | -73.0569 | 41.2222 |
| *Lampropeltis triangulum* | -77.1414 | 41.23152 |
| *Lampropeltis triangulum* | -77.78304 | 41.25184 |
| *Lampropeltis triangulum* | -69.984126 | 41.2625 |
| *Lampropeltis triangulum* | -81.55279 | 41.2749 |
| *Lampropeltis triangulum* | -73.0261 | 41.2783 |
| *Lampropeltis triangulum* | -101.68694 | 41.27917 |
| *Lampropeltis triangulum* | -76.40823 | 41.27917 |
| *Lampropeltis triangulum* | -76.35177 | 41.28108 |
| *Lampropeltis triangulum* | -79.59011 | 41.29375 |
| *Lampropeltis triangulum* | -70.0133 | 41.2983 |
| *Lampropeltis triangulum* | -101.892 | 41.3006 |
| *Lampropeltis triangulum* | -72.9246 | 41.3113 |
| *Lampropeltis triangulum* | -76.19416 | 41.31154 |
| *Lampropeltis triangulum* | -77.7861 | 41.31369 |
| *Lampropeltis triangulum* | -78.39747 | 41.31566 |
| *Lampropeltis triangulum* | -81.40867 | 41.32274 |
| *Lampropeltis triangulum* | -77.11546 | 41.32888 |
| *Lampropeltis triangulum* | -79.16644 | 41.33344 |
| *Lampropeltis triangulum* | -72.9647 | 41.3375 |
| *Lampropeltis triangulum* | -77.36797 | 41.33972 |
| *Lampropeltis triangulum* | -70.7453 | 41.3431 |
| *Lampropeltis triangulum* | -72.8483 | 41.3475 |
| *Lampropeltis triangulum* | -70.511 | 41.745 |
| *Lampropeltis triangulum* | -84.015938 | 41.367592 |
| *Lampropeltis triangulum* | -79.63 | 41.37 |
| *Lampropeltis triangulum* | -70.519 | 41.368 |
| *Lampropeltis triangulum* | -73.4547 | 41.3778 |
| *Lampropeltis triangulum* | -75.63319 | 41.37955 |
| *Lampropeltis triangulum* | -72.7917 | 41.3936 |
| *Lampropeltis triangulum* | -79.62422 | 41.39701 |
| *Lampropeltis triangulum* | -84.288588 | 41.39954 |
| *Lampropeltis triangulum* | -79.0797 | 41.4044 |
| *Lampropeltis triangulum* | -70.6167 | 41.4167 |
| *Lampropeltis triangulum* | -78.6537 | 41.4187 |
| *Lampropeltis triangulum* | -73.95498 | 41.41983 |
| *Lampropeltis triangulum* | -75.07726 | 41.4203 |
| *Lampropeltis triangulum* | -72.9975 | 41.4217 |
| *Lampropeltis triangulum* | -76.05688 | 41.42288 |
| *Lampropeltis triangulum* | -77.49052 | 41.42328 |
| *Lampropeltis triangulum* | -79.36848 | 41.4282 |
| *Lampropeltis triangulum* | -78.38474 | 41.43225 |
| *Lampropeltis triangulum* | -71.69371 | 41.436699 |
| *Lampropeltis triangulum* | -71.69371 | 41.4367 |
| *Lampropeltis triangulum* | -70.6042 | 41.4542 |
| *Lampropeltis triangulum* | -75.049 | 41.45924 |
| *Lampropeltis triangulum* | -72.478257 | 41.464332 |
| *Lampropeltis triangulum* | -78.34602 | 41.47242 |
| *Lampropeltis triangulum* | -79.49203 | 41.47305 |
| *Lampropeltis triangulum* | -73.87403 | 41.48572 |
| *Lampropeltis triangulum* | -71.3065 | 41.4893 |
| *Lampropeltis triangulum* | -71.3133 | 41.49 |
| *Lampropeltis triangulum* | -81.674336 | 41.499479 |
| *Lampropeltis triangulum* | -81.67434 | 41.49948 |
| *Lampropeltis triangulum* | -75.99319 | 41.5076 |
| *Lampropeltis triangulum* | -74.06023 | 41.508236 |
| *Lampropeltis triangulum* | -74.06023 | 41.50824 |
| *Lampropeltis triangulum* | -71.1717 | 41.51 |
| *Lampropeltis triangulum* | -75.96162 | 41.51435 |
| *Lampropeltis triangulum* | -72.7125 | 41.5172 |
| *Lampropeltis triangulum* | -93.610214 | 41.519989 |
| *Lampropeltis triangulum* | -79.43526 | 41.52755 |
| *Lampropeltis triangulum* | -75.97753 | 41.53238 |
| *Lampropeltis triangulum* | -77.71523 | 41.53974 |
| *Lampropeltis triangulum* | -75.95262 | 41.54192 |
| *Lampropeltis triangulum* | -76.41549 | 41.55985 |
| *Lampropeltis triangulum* | -79.688705 | 41.566664 |
| *Lampropeltis triangulum* | -93.61481 | 41.57618 |
| *Lampropeltis triangulum* | -79.5797 | 41.5922 |
| *Lampropeltis triangulum* | -80.35632 | 41.59309 |
| *Lampropeltis triangulum* | -78.18649 | 41.60202 |
| *Lampropeltis triangulum* | -75.83443 | 41.60532 |
| *Lampropeltis triangulum* | -80.36033 | 41.61811 |
| *Lampropeltis triangulum* | -75.48994 | 41.61846 |
| *Lampropeltis triangulum* | -78.82951 | 41.61867 |
| *Lampropeltis triangulum* | -77.70678 | 41.63547 |
| *Lampropeltis triangulum* | -75.73563 | 41.63577 |
| *Lampropeltis triangulum* | -75.70544 | 41.63953 |
| *Lampropeltis triangulum* | -80.42778 | 41.6424 |
| *Lampropeltis triangulum* | -80.47674 | 41.65542 |
| *Lampropeltis triangulum* | -80.46599 | 41.66235 |
| *Lampropeltis triangulum* | -73.52893 | 41.67153 |
| *Lampropeltis triangulum* | -75.05116 | 41.68117 |
| *Lampropeltis triangulum* | -87.91524 | 41.68863 |
| *Lampropeltis triangulum* | -77.63962 | 41.6897 |
| *Lampropeltis triangulum* | -79.35345 | 41.69272 |
| *Lampropeltis triangulum* | -70.0278 | 41.7 |
| *Lampropeltis triangulum* | -75.18713 | 41.70137 |
| *Lampropeltis triangulum* | -76.57323 | 41.70367 |
| *Lampropeltis triangulum* | -70.375 | 41.7056 |
| *Lampropeltis triangulum* | -77.57953 | 41.71523 |
| *Lampropeltis triangulum* | -87.671847 | 41.731467 |
| *Lampropeltis triangulum* | -102.254787 | 41.73685 |
| *Lampropeltis triangulum* | -73.165995 | 41.741766 |
| *Lampropeltis triangulum* | -70.109 | 41.745 |
| *Lampropeltis triangulum* | -70.11346 | 41.75211 |
| *Lampropeltis triangulum* | -77.55762 | 41.75245 |
| *Lampropeltis triangulum* | -77.99194 | 41.78356 |
| *Lampropeltis triangulum* | -73.699591 | 41.790063 |
| *Lampropeltis triangulum* | -70.64748 | 41.7921 |
| *Lampropeltis triangulum* | -70.64822 | 41.79885 |
| *Lampropeltis triangulum* | -75.80342 | 41.80392 |
| *Lampropeltis triangulum* | -75.19628 | 41.81471 |
| *Lampropeltis triangulum* | -73.13122 | 41.81603 |
| *Lampropeltis triangulum* | -75.32195 | 41.82824 |
| *Lampropeltis triangulum* | -75.21899 | 41.83493 |
| *Lampropeltis triangulum* | -75.84074 | 41.83602 |
| *Lampropeltis triangulum* | -87.62628 | 41.87887 |
| *Lampropeltis triangulum* | -100.28288 | 41.88505 |
| *Lampropeltis triangulum* | -80.785173 | 41.898094 |
| *Lampropeltis triangulum* | -78.06784 | 41.90259 |
| *Lampropeltis triangulum* | -72.4703 | 41.9039 |
| *Lampropeltis triangulum* | -78.10509 | 41.9179 |
| *Lampropeltis triangulum* | -79.61672 | 41.93252 |
| *Lampropeltis triangulum* | -80.47444 | 41.94583 |
| *Lampropeltis triangulum* | -78.91195 | 41.98772 |
| *Lampropeltis triangulum* | -72.5833 | 42.05 |
| *Lampropeltis triangulum* | -70.1792 | 42.0583 |
| *Lampropeltis triangulum* | -80.19472 | 42.065 |
| *Lampropeltis triangulum* | -80.18222 | 42.07194 |
| *Lampropeltis triangulum* | -78.43028 | 42.07639 |
| *Lampropeltis triangulum* | -80.14361 | 42.09278 |
| *Lampropeltis triangulum* | -79.96417 | 42.10184 |
| *Lampropeltis triangulum* | -73.3556 | 42.1103 |
| *Lampropeltis triangulum* | -84.399022 | 42.115622 |
| *Lampropeltis triangulum* | -71.9706 | 42.1356 |
| *Lampropeltis triangulum* | -71.16479 | 42.1711 |
| *Lampropeltis triangulum* | -71.1181 | 42.1917 |
| *Lampropeltis triangulum* | -72.1917 | 42.2125 |
| *Lampropeltis triangulum* | -86.014096 | 42.231079 |
| *Lampropeltis triangulum* | -76.86315 | 42.23155 |
| *Lampropeltis triangulum* | -71.34151 | 42.2541 |
| *Lampropeltis triangulum* | -81.852264 | 42.276039 |
| *Lampropeltis triangulum* | -71.4375 | 42.2972 |
| *Lampropeltis triangulum* | -76.48101 | 42.30391 |
| *Lampropeltis triangulum* | -76.47845 | 42.31633 |
| *Lampropeltis triangulum* | -76.33814 | 42.33031 |
| *Lampropeltis triangulum* | -71.2 | 42.3306 |
| *Lampropeltis triangulum* | -71.25 | 42.3472 |
| *Lampropeltis triangulum* | -76.2006 | 42.35566 |
| *Lampropeltis triangulum* | -71.2361 | 42.3764 |
| *Lampropeltis triangulum* | -98.2344 | 42.3783 |
| *Lampropeltis triangulum* | -71.3605 | 42.3861 |
| *Lampropeltis triangulum* | -76.32639 | 42.38861 |
| *Lampropeltis triangulum* | -98.1266 | 42.3935 |
| *Lampropeltis triangulum* | -76.35052 | 42.39393 |
| *Lampropeltis triangulum* | -84.06792 | 42.39851 |
| *Lampropeltis triangulum* | -76.4167 | 42.4167 |
| *Lampropeltis triangulum* | -76.50293 | 42.41685 |
| *Lampropeltis triangulum* | -76.51806 | 42.41931 |
| *Lampropeltis triangulum* | -76.47563 | 42.42621 |
| *Lampropeltis triangulum* | -76.38849 | 42.42766 |
| *Lampropeltis triangulum* | -71.9764 | 42.4306 |
| *Lampropeltis triangulum* | -76.50948 | 42.4381 |
| *Lampropeltis triangulum* | -85.57129 | 42.43876 |
| *Lampropeltis triangulum* | -71.64351 | 42.44609 |
| *Lampropeltis triangulum* | -71.0667 | 42.4583 |
| *Lampropeltis triangulum* | -85.968151 | 42.465745 |
| *Lampropeltis triangulum* | -70.95 | 42.4667 |
| *Lampropeltis triangulum* | -76.45218 | 42.46748 |
| *Lampropeltis triangulum* | -82.41006 | 42.47185 |
| *Lampropeltis triangulum* | -77.65611 | 42.47216 |
| *Lampropeltis triangulum* | -76.50948 | 42.48157 |
| *Lampropeltis triangulum* | -72.1875 | 42.4875 |
| *Lampropeltis triangulum* | -71.2767 | 42.4906 |
| *Lampropeltis triangulum* | -76.45767 | 42.49128 |
| *Lampropeltis triangulum* | -71.5278 | 42.4917 |
| *Lampropeltis triangulum* | -76.54 | 42.49278 |
| *Lampropeltis triangulum* | -71.5833 | 42.5 |
| *Lampropeltis triangulum* | -100.53722 | 42.50306 |
| *Lampropeltis triangulum* | -71.30151 | 42.5051 |
| *Lampropeltis triangulum* | -70.929004 | 42.527941 |
| *Lampropeltis triangulum* | -85.355013 | 42.535554 |
| *Lampropeltis triangulum* | -76.54981 | 42.53719 |
| *Lampropeltis triangulum* | -76.65966 | 42.54401 |
| *Lampropeltis triangulum* | -76.61374 | 42.54623 |
| *Lampropeltis triangulum* | -85.974288 | 42.547765 |
| *Lampropeltis triangulum* | -76.72113 | 42.5494 |
| *Lampropeltis triangulum* | -76.62864 | 42.55045 |
| *Lampropeltis triangulum* | -70.8806 | 42.5583 |
| *Lampropeltis triangulum* | -80.24294 | 42.5613 |
| *Lampropeltis triangulum* | -71.93352 | 42.56309 |
| *Lampropeltis triangulum* | -70.8111 | 42.5639 |
| *Lampropeltis triangulum* | -76.51583 | 42.57306 |
| *Lampropeltis triangulum* | -97.91639 | 42.5925 |
| *Lampropeltis triangulum* | -72.00852 | 42.59839 |
| *Lampropeltis triangulum* | -99.13944 | 42.59944 |
| *Lampropeltis triangulum* | -76.17528 | 42.60083 |
| *Lampropeltis triangulum* | -70.6486 | 42.6111 |
| *Lampropeltis triangulum* | -76.75843 | 42.6225 |
| *Lampropeltis triangulum* | -100.55028 | 42.629 |
| *Lampropeltis triangulum* | -70.8436 | 42.6361 |
| *Lampropeltis triangulum* | -70.9515 | 42.6576 |
| *Lampropeltis triangulum* | -77.53838 | 42.6593 |
| *Lampropeltis triangulum* | -80.3588 | 42.67359 |
| *Lampropeltis triangulum* | -70.8417 | 42.6792 |
| *Lampropeltis triangulum* | -72.3394 | 42.6819 |
| *Lampropeltis triangulum* | -81.79998 | 42.68475 |
| *Lampropeltis triangulum* | -99.86369 | 42.72478 |
| *Lampropeltis triangulum* | -77.77251 | 42.73018 |
| *Lampropeltis triangulum* | -98.02694 | 42.75472 |
| *Lampropeltis triangulum* | -108.7761 | 42.75806 |
| *Lampropeltis triangulum* | -84.745104 | 42.766248 |
| *Lampropeltis triangulum* | -71.0778 | 42.7761 |
| *Lampropeltis triangulum* | -71.97 | 42.7833 |
| *Lampropeltis triangulum* | -77.81233 | 42.79776 |
| *Lampropeltis triangulum* | -97.7289 | 42.8214 |
| *Lampropeltis triangulum* | -70.985 | 42.8625 |
| *Lampropeltis triangulum* | -100.52972 | 42.89222 |
| *Lampropeltis triangulum* | -78.82111 | 42.91639 |
| *Lampropeltis triangulum* | -79.30275 | 42.918344 |
| *Lampropeltis triangulum* | -81.33556 | 42.94361 |
| *Lampropeltis triangulum* | -85.48887 | 42.95455 |
| *Lampropeltis triangulum* | -85.50124 | 42.9784 |
| *Lampropeltis triangulum* | -83.717413 | 42.992352 |
| *Lampropeltis triangulum* | -78.18472 | 42.99611 |
| *Lampropeltis triangulum* | -77.59712 | 42.99663 |
| *Lampropeltis triangulum* | -81.2613 | 42.99875 |
| *Lampropeltis triangulum* | -87.893268 | 43.044296 |
| *Lampropeltis triangulum* | -80.56343 | 43.04478 |
| *Lampropeltis triangulum* | -75.846728 | 43.054297 |
| *Lampropeltis triangulum* | -89.411381 | 43.062467 |
| *Lampropeltis triangulum* | -77.44952 | 43.10434 |
| *Lampropeltis triangulum* | -71.8958 | 43.1139 |
| *Lampropeltis triangulum* | -71.8958 | 43.1139 |
| *Lampropeltis triangulum* | -79.26267 | 43.11696 |
| *Lampropeltis triangulum* | -79.262667 | 43.116961 |
| *Lampropeltis triangulum* | -79.048219 | 43.146965 |
| *Lampropeltis triangulum* | -88.304215 | 43.147874 |
| *Lampropeltis triangulum* | -88.114081 | 43.181347 |
| *Lampropeltis triangulum* | -77.86417 | 43.18833 |
| *Lampropeltis triangulum* | -77.58682 | 43.19216 |
| *Lampropeltis triangulum* | -70.6925 | 43.2231 |
| *Lampropeltis triangulum* | -86.254171 | 43.240193 |
| *Lampropeltis triangulum* | -71.8172 | 43.2806 |
| *Lampropeltis triangulum* | -80.4546 | 43.28939 |
| *Lampropeltis triangulum* | -71.5658 | 43.3369 |
| *Lampropeltis triangulum* | -88.043225 | 43.432418 |
| *Lampropeltis triangulum* | -76.43583 | 43.44028 |
| *Lampropeltis triangulum* | -88.702004 | 43.444665 |
| *Lampropeltis triangulum* | -70.7942 | 43.4667 |
| *Lampropeltis triangulum* | -73.77377 | 43.49541 |
| *Lampropeltis triangulum* | -81.383335 | 43.549999 |
| *Lampropeltis triangulum* | -90.89387 | 43.555899 |
| *Lampropeltis triangulum* | -70.228 | 43.584 |
| *Lampropeltis triangulum* | -79.62009 | 43.5742 |
| *Lampropeltis triangulum* | -83.890998 | 43.595687 |
| *Lampropeltis triangulum* | -103.41 | 43.61 |
| *Lampropeltis triangulum* | -72.97272 | 43.611788 |
| *Lampropeltis triangulum* | -89.681612 | 43.61783 |
| *Lampropeltis triangulum* | -103.36839 | 43.64369 |
| *Lampropeltis triangulum* | -79.36755 | 43.67563 |
| *Lampropeltis triangulum* | -79.367552 | 43.675632 |
| *Lampropeltis triangulum* | -71.1172 | 43.6853 |
| *Lampropeltis triangulum* | -79.372787 | 43.690839 |
| *Lampropeltis triangulum* | -79.50943 | 43.70089 |
| *Lampropeltis triangulum* | -103.34031 | 43.7062 |
| *Lampropeltis triangulum* | -88.464364 | 43.772334 |
| *Lampropeltis triangulum* | -102.55 | 43.78 |
| *Lampropeltis triangulum* | -90.076413 | 43.798216 |
| *Lampropeltis triangulum* | -79.11993 | 43.799474 |
| *Lampropeltis triangulum* | -73.2983 | 43.8039 |
| *Lampropeltis triangulum* | -71.84629 | 43.81084 |
| *Lampropeltis triangulum* | -79.99343 | 43.86225 |
| *Lampropeltis triangulum* | -80.11392 | 43.91585 |
| *Lampropeltis triangulum* | -69.5097 | 43.9575 |
| *Lampropeltis triangulum* | -81.51346 | 43.96088 |
| *Lampropeltis triangulum* | -79.62204 | 43.98806 |
| *Lampropeltis triangulum* | -102.24 | 43.99 |
| *Lampropeltis triangulum* | -80.03021 | 43.99084 |
| *Lampropeltis triangulum* | -77.45 | 44 |
| *Lampropeltis triangulum* | -79.56329 | 44.00796 |
| *Lampropeltis triangulum* | -77.138615 | 44.008003 |
| *Lampropeltis triangulum* | -107.75331 | 44.01676 |
| *Lampropeltis triangulum* | -72.008616 | 44.030985 |
| *Lampropeltis triangulum* | -77.73807 | 44.03275 |
| *Lampropeltis triangulum* | -91.99837 | 44.09016 |
| *Lampropeltis triangulum* | -70.494026 | 44.130055 |
| *Lampropeltis triangulum* | -71.0117 | 44.1644 |
| *Lampropeltis triangulum* | -80.81667 | 44.16666 |
| *Lampropeltis triangulum* | -80.819854 | 44.175723 |
| *Lampropeltis triangulum* | -69.9572 | 44.2386 |
| *Lampropeltis triangulum* | -76.95613 | 44.23918 |
| *Lampropeltis triangulum* | -76.5067 | 44.24199 |
| *Lampropeltis triangulum* | -76.38483 | 44.26586 |
| *Lampropeltis triangulum* | -79.99363 | 44.3012 |
| *Lampropeltis triangulum* | -85.41835 | 44.32323 |
| *Lampropeltis triangulum* | -69.8608 | 44.3244 |
| *Lampropeltis triangulum* | -80.10522 | 44.32616 |
| *Lampropeltis triangulum* | -84.185559 | 44.326557 |
| *Lampropeltis triangulum* | -79.28713 | 44.32747 |
| *Lampropeltis triangulum* | -76.150532 | 44.393438 |
| *Lampropeltis triangulum* | -70.0725 | 44.4206 |
| *Lampropeltis triangulum* | -78.71666 | 44.4666 |
| *Lampropeltis triangulum* | -77.68167 | 44.48432 |
| *Lampropeltis triangulum* | -79.7277 | 44.53162 |
| *Lampropeltis triangulum* | -78.54732 | 44.53849 |
| *Lampropeltis triangulum* | -76.10972 | 44.54167 |
| *Lampropeltis triangulum* | -78.5071 | 44.5923 |
| *Lampropeltis triangulum* | -79.416672 | 44.600002 |
| *Lampropeltis triangulum* | -80.58831 | 44.6081 |
| *Lampropeltis triangulum* | -69.33827 | 44.614398 |
| *Lampropeltis triangulum* | -68.5553 | 44.6711 |
| *Lampropeltis triangulum* | -77.423916 | 44.675672 |
| *Lampropeltis triangulum* | -76.68045 | 44.685498 |
| *Lampropeltis triangulum* | -76.92188 | 44.7223 |
| *Lampropeltis triangulum* | -78.6333 | 44.75 |
| *Lampropeltis triangulum* | -79.932404 | 44.766237 |
| *Lampropeltis triangulum* | -79.793873 | 44.814237 |
| *Lampropeltis triangulum* | -81.32546 | 44.86628 |
| *Lampropeltis triangulum* | -79.6 | 44.8666 |
| *Lampropeltis triangulum* | -81.326294 | 44.87047 |
| *Lampropeltis triangulum* | -79.759 | 44.875 |
| *Lampropeltis triangulum* | -91.936102 | 44.884203 |
| *Lampropeltis triangulum* | -85.20666 | 44.9792 |
| *Lampropeltis triangulum* | -79.93316 | 44.99417 |
| *Lampropeltis triangulum* | -77.85688 | 45.06442 |
| *Lampropeltis triangulum* | -79.542131 | 45.091784 |
| *Lampropeltis triangulum* | -81.54461 | 45.11898 |
| *Lampropeltis triangulum* | -79.60693 | 45.15372 |
| *Lampropeltis triangulum* | -108.91651 | 45.18686 |
| *Lampropeltis triangulum* | -79.78573 | 45.21055 |
| *Lampropeltis triangulum* | -108.93639 | 45.21711 |
| *Lampropeltis triangulum* | -108.84547 | 45.21944 |
| *Lampropeltis triangulum* | -81.66886 | 45.25858 |
| *Lampropeltis triangulum* | -108.80646 | 45.32413 |
| *Lampropeltis triangulum* | -108.82583 | 45.32589 |
| *Lampropeltis triangulum* | -80.333 | 45.4 |
| *Lampropeltis triangulum* | -76.34817 | 45.43381 |
| *Lampropeltis triangulum* | -77.67498 | 45.48815 |
| *Lampropeltis triangulum* | -80.5 | 45.56666 |
| *Lampropeltis triangulum* | -75.931031 | 45.683105 |
| *Lampropeltis triangulum* | -108.957 | 45.77065 |
| *Lampropeltis triangulum* | -84.72788 | 45.8688 |
| *Lampropeltis triangulum* | -81.90986 | 45.94691 |
| *Lampropeltis triangulum* | -108.50611 | 46.01906 |
| *Lampropeltis triangulum* | -77.35 | 46.05 |
| *Lampropeltis triangulum* | -82.94661 | 46.18958 |
| *Lampropeltis triangulum* | -82.07517 | 46.21037 |
| *Lampropeltis triangulum* | -82.00961 | 46.25119 |
| *Lampropeltis triangulum* | -81.36245 | 46.25505 |
| *Lampropeltis triangulum* | -84.34535 | 46.55253 |
| *Lampropeltis triangulum* | -83.98333 | 46.56666 |
| *Lampropeltis triangulum* | -107.126301 | 47.265832 |
| *Lampropeltis triangulum* | -83.58183 | 95.68947 |
| *Lampropeltis triangulum* | -86.499544 | 39.184958 |

**S1D Dataset**

Occurrence records of *Pyhton bivittatus* in Asia.

| Specie | Latitude | Longitude |
| --- | --- | --- |
| *Python bivittatus* | 24.998056 | 92.912500 |
| *Python bivittatus* | -8.859433 | 117.164666 |
| *Python bivittatus* | -8.838104 | 117.568514 |
| *Python bivittatus* | -8.665266 | 117.388646 |
| *Python bivittatus* | -8.632737 | 116.338484 |
| *Python bivittatus* | -8.483128 | 118.491940 |
| *Python bivittatus* | -8.382138 | 115.099536 |
| *Python bivittatus* | -8.053120 | 113.000873 |
| *Python bivittatus* | -7.766933 | 112.515978 |
| *Python bivittatus* | -7.671499 | 111.174150 |
| *Python bivittatus* | -7.452343 | 107.783784 |
| *Python bivittatus* | -7.297592 | 109.178826 |
| *Python bivittatus* | -7.286752 | 109.536544 |
| *Python bivittatus* | -7.213922 | 110.157908 |
| *Python bivittatus* | -7.135182 | 107.236593 |
| *Python bivittatus* | -6.931285 | 108.415443 |
| *Python bivittatus* | -6.830753 | 107.788860 |
| *Python bivittatus* | -6.793587 | 106.947914 |
| *Python bivittatus* | -6.695664 | 106.152052 |
| *Python bivittatus* | -6.655009 | 110.897187 |
| *Python bivittatus* | -6.562511 | 107.633332 |
| *Python bivittatus* | -5.362717 | 119.949851 |
| *Python bivittatus* | -5.016832 | 119.698097 |
| *Python bivittatus* | -4.946979 | 120.234992 |
| *Python bivittatus* | -4.878407 | 120.206498 |
| *Python bivittatus* | -4.786569 | 119.834914 |
| *Python bivittatus* | -4.645170 | 120.124469 |
| *Python bivittatus* | -4.461421 | 119.805975 |
| *Python bivittatus* | -4.339598 | 120.041569 |
| *Python bivittatus* | -4.083042 | 119.743733 |
| *Python bivittatus* | 10.182009 | 106.137358 |
| *Python bivittatus* | 10.729189 | 105.493621 |
| *Python bivittatus* | 11.419119 | 107.426548 |
| *Python bivittatus* | 12.271853 | 104.114356 |
| *Python bivittatus* | 12.868685 | 99.400107 |
| *Python bivittatus* | 12.884984 | 99.632659 |
| *Python bivittatus* | 12.932586 | 99.644916 |
| *Python bivittatus* | 13.018830 | 100.041552 |
| *Python bivittatus* | 13.260658 | 103.500077 |
| *Python bivittatus* | 13.894176 | 105.138962 |
| *Python bivittatus* | 14.938947 | 105.857754 |
| *Python bivittatus* | 18.724241 | 109.942864 |
| *Python bivittatus* | 18.737548 | 108.936175 |
| *Python bivittatus* | 18.831778 | 109.074820 |
| *Python bivittatus* | 19.045740 | 109.519597 |
| *Python bivittatus* | 19.447021 | 109.618171 |
| *Python bivittatus* | 19.885153 | 110.326863 |
| *Python bivittatus* | 20.421833 | 103.608400 |
| *Python bivittatus* | 20.456098 | 103.636271 |
| *Python bivittatus* | 20.486018 | 103.639996 |
| *Python bivittatus* | 20.487727 | 103.708185 |
| *Python bivittatus* | 20.672818 | 86.912322 |
| *Python bivittatus* | 21.317582 | 101.578590 |
| *Python bivittatus* | 21.597223 | 101.698607 |
| *Python bivittatus* | 22.072345 | 99.985769 |
| *Python bivittatus* | 22.130548 | 99.994302 |
| *Python bivittatus* | 22.194029 | 113.499377 |
| *Python bivittatus* | 22.235334 | 113.944496 |
| *Python bivittatus* | 22.253876 | 114.159245 |
| *Python bivittatus* | 22.253885 | 114.203510 |
| *Python bivittatus* | 22.329110 | 99.584259 |
| *Python bivittatus* | 22.361410 | 114.313170 |
| *Python bivittatus* | 22.530736 | 88.248963 |
| *Python bivittatus* | 22.703206 | 103.939636 |
| *Python bivittatus* | 23.202570 | 98.953523 |
| *Python bivittatus* | 23.274585 | 102.088305 |
| *Python bivittatus* | 23.298693 | 114.380744 |
| *Python bivittatus* | 23.751351 | 117.213420 |
| *Python bivittatus* | 23.832309 | 101.672917 |
| *Python bivittatus* | 24.134221 | 88.703981 |
| *Python bivittatus* | 24.201081 | 97.909621 |
| *Python bivittatus* | 24.341376 | 91.797711 |
| *Python bivittatus* | 24.442728 | 113.951808 |
| *Python bivittatus* | 24.540862 | 97.826633 |
| *Python bivittatus* | 24.717094 | 114.447716 |
| *Python bivittatus* | 25.006769 | 106.238786 |
| *Python bivittatus* | 25.041358 | 85.298909 |
| *Python bivittatus* | 25.128674 | 106.607150 |
| *Python bivittatus* | 25.430961 | 106.249524 |
| *Python bivittatus* | 25.686143 | 97.505975 |
| *Python bivittatus* | 26.473842 | 90.564361 |
| *Python bivittatus* | 26.633628 | 118.179152 |
| *Python bivittatus* | 26.811949 | 90.672525 |
| *Python bivittatus* | 26.836048 | 90.734574 |
| *Python bivittatus* | 27.145697 | 90.688580 |
| *Python bivittatus* | 27.534980 | 84.358708 |
| *Python bivittatus* | 28.546131 | 77.238128 |

**S1E Dataset**

Occurrence records of *Python regius* in Africa.

| Species | Longitude | Latitude |
| --- | --- | --- |
| *Python regius* | 2.517 | 8.083 |
| *Python regius* | -3 | 9.267 |
| *Python regius* | -3.437855 | 9.118069 |
| *Python regius* | -0.233 | 5.617 |
| *Python regius* | -0.2 | 5.667 |
| *Python regius* | -0.21667 | 5.91667 |
| *Python regius* | 0.55407 | 8.32512 |
| *Python regius* | 0.51624 | 8.36917 |
| *Python regius* | -1.4588 | 9.16464 |
| *Python regius* | -0.194 | 5.646667 |
| *Python regius* | -0.017 | 6.1 |
| *Python regius* | -14.45 | 10.754 |
| *Python regius* | 7.95268 | 5.12502 |
| *Python regius* | 3.917207 | 7.395481 |
| *Python regius* | 8.325 | 4.95 |
| *Python regius* | -11.8 | 8.46667 |
| *Python regius* | 0.530809 | 8.28176 |
| *Python regius* | -15.788805 | 13.536507 |
| *Python regius* | 6.381893 | 5.191417 |
| *Python regius* | -16.785392 | 13.373892 |
| *Python regius* | -16.476 | 13.184 |
| *Python regius* | 21.762922 | -4.037068 |
| *Python regius* | -16.55496 | 14.00099 |
| *Python regius* | 2.166667 | 6.916667 |
| *Python regius* | 1.100276 | 6.593297 |
| *Python regius* | 1.216667 | 6.433333 |
| *Python regius* | 0.009143 | 11.098892 |
| *Python regius* | 0.608575 | 7.586604 |
| *Python regius* | 1.200118 | 6.387714 |
| *Python regius* | 1.404467 | 6.70404 |
| *Python regius* | 1.081355 | 6.154274 |
| *Python regius* | 1.622651 | 7.024516 |
