# Supplementary material for: Pet snakes illegally marketed in Brazil: Climatic viability and establishment risk
Source: PLoS One. 2017 Aug 17;12(8):e0183143. doi: 10.1371/journal.pone.0183143 (PMC5560532; doi:10.1371/journal.pone.0183143)
Supplement: S1 Tables — S1A Table—Environmental variables and their respective PCA axes used as environmental layers for American species. S1B Table—Environmental variables and their respective PCA axes used as environmental layers for the Asian species. S1C Table—Environmental variables and their respective PCA axes used as environmental layers for the African species. (DOCX) [file pone.0183143.s002.docx]

**S1 Tables - Environmental variables and their respective PCA axes used as environmental layers.**

**S1A Table**

Environmental variables and their respective PCA axes used as environmental layers for American species (*Lampropeltis getula*. *Lampropeltis triangulum* and *Pantherophis guttatus*).

| Enviromental variables | Principal components | |  |  |  |  |
| --- | --- | --- | --- | --- | --- | --- |
|  | PC1 | PC2 | PC3 | PC4 | PC5 | PC6 |
| Altitude | 0.048 | -0.017 | -0.608 | -0.493 | -0.305 | -0.106 |
| Mean temperature warmest quarter | -0.245 | 0.219 | 0.271 | -0.056 | 0.022 | -0.061 |
| Mean temperature coldest quarter | -0.285 | 0.112 | -0.061 | -0.050 | 0.141 | 0.075 |
| Annual precipitation | -0.246 | -0.255 | 0.011 | 0.106 | -0.176 | -0.097 |
| Precipitation wettest period | -0.255 | -0.143 | -0.079 | 0.263 | -0.248 | -0.137 |
| Precipitation driest period | -0.133 | -0.398 | 0.160 | -0.251 | -0.020 | 0.047 |
| Precipitation seasonality | -0.020 | 0.324 | -0.300 | 0.444 | -0.353 | 0.002 |
| Precipitation wettest quarter | -0.254 | -0.154 | -0.076 | 0.254 | -0.245 | -0.141 |
| Precipitation driest quarter | -0.145 | -0.395 | 0.154 | -0.234 | -0.026 | 0.022 |
| Precipitation warmest quarter | -0.198 | -0.200 | 0.115 | -0.054 | -0.497 | 0.541 |
| Precipitation coldest quarter | -0.189 | -0.268 | -0.015 | 0.124 | 0.072 | -0.671 |
| Annual mean tempretaure | -0.280 | 0.153 | 0.057 | -0.059 | 0.094 | 0.035 |
| Mean diurnal range | -0.084 | 0.353 | 0.073 | -0.464 | -0.336 | -0.267 |
| Isothermality | -0.274 | 0.094 | -0.184 | 0.003 | 0.033 | 0.025 |
| Temperature seasonality | 0.270 | -0.022 | 0.277 | 0.047 | -0.200 | -0.153 |
| Maximum temperature warmest period | -0.225 | 0.261 | 0.278 | -0.127 | -0.036 | -0.148 |
| Minimum temperature coldest period | -0.286 | 0.085 | -0.073 | -0.023 | 0.194 | 0.082 |
| Temperature annual range | 0.256 | 0.059 | 0.300 | -0.055 | -0.305 | -0.222 |
| Mean temperature wettest quarter | -0.215 | 0.236 | 0.299 | 0.101 | -0.153 | 0.096 |
| Mean temperature driest quarter | -0.275 | 0.085 | -0.091 | -0.149 | 0.192 | -0.034 |
|  |  |  |  |  |  |  |
| Proportion explained by each PC | 56.802 | 19.551 | 7.656 | 6.199 | 3.679 | 2.755 |
| Accumulated variation proportion | 56.802 | 76.353 | 84.009 | 90.209 | 93.888 | 96.643 |
| Principal components eigenvalues | 11.360 | 3.910 | 1.531 | 1.240 | 0.736 | 0.551 |

**S1B Table**

Environmental variables and their respective PCA axes used as environmental layers for the Asian species (*Python bivittatus*).

| Enviromental variables | Principal components | | |  |  |  |
| --- | --- | --- | --- | --- | --- | --- |
|  | PC1 | PC2 | PC3 | PC4 | PC5 | PC6 |
| Altitude | 0.055 | -0.052 | -0.382 | -0.659 | -0.100 | 0.181 |
| Mean temperature warmest quarter | -0.241 | -0.251 | 0.136 | 0.195 | -0.128 | 0.009 |
| Mean temperature coldest quarter | -0.285 | -0.143 | 0.052 | -0.088 | 0.139 | 0.030 |
| Annual precipitation | -0.246 | 0.259 | -0.166 | 0.081 | -0.081 | -0.027 |
| Precipitation wettest period | -0.230 | 0.123 | -0.385 | 0.208 | 0.027 | -0.237 |
| Precipitation driest period | -0.173 | 0.346 | 0.189 | -0.124 | -0.320 | 0.097 |
| Precipitation seasonality | -0.011 | -0.297 | -0.520 | 0.018 | -0.205 | -0.298 |
| Precipitation wettest quarter | -0.233 | 0.142 | -0.369 | 0.197 | 0.028 | -0.180 |
| Precipitation driest quarter | -0.180 | 0.344 | 0.180 | -0.119 | -0.304 | 0.091 |
| Precipitation warmest quarter | -0.194 | 0.211 | -0.327 | 0.160 | -0.097 | 0.609 |
| Precipitation coldest quarter | -0.185 | 0.300 | 0.144 | -0.127 | -0.247 | -0.532 |
| Annual mean tempretaure | -0.279 | -0.187 | 0.070 | -0.004 | 0.039 | 0.040 |
| Mean diurnal range | 0.028 | -0.384 | -0.009 | -0.240 | -0.540 | 0.017 |
| Isothermality | -0.271 | -0.008 | 0.043 | -0.254 | -0.086 | -0.052 |
| Temperature seasonality | 0.276 | 0.065 | 0.006 | 0.243 | -0.267 | -0.041 |
| Maximum temperature warmest period | -0.212 | -0.293 | 0.169 | 0.202 | -0.202 | -0.050 |
| Minimum temperature coldest period | -0.289 | -0.114 | 0.069 | -0.077 | 0.171 | 0.020 |
| Temperature annual range | 0.275 | -0.005 | -0.002 | 0.213 | -0.339 | -0.053 |
| Mean temperature wettest quarter | -0.224 | -0.202 | 0.034 | 0.272 | -0.232 | 0.299 |
| Mean temperature driest quarter | -0.269 | -0.155 | 0.116 | -0.110 | 0.170 | -0.121 |
|  |  |  |  |  |  |  |
| Proportion explained by each PC | 54.348 | 19.544 | 8.602 | 7.110 | 4.225 | 1.895 |
| Accumulated variation proportion | 54.348 | 73.892 | 82.494 | 89.605 | 93.829 | 95.725 |
| Principal components eigenvalues | 10.870 | 3.909 | 1.720 | 1.422 | 0.845 | 0.379 |

**S1C Table**

Environmental variables and their respective PCA axes used as environmental layers for the African species (*Python regius*).

| Enviromental variables | Principal components | | |  |  |  |  |  |
| --- | --- | --- | --- | --- | --- | --- | --- | --- |
|  | PC1 | PC2 | PC3 | PC4 | PC5 | PC6 | PC7 | PC8 |
| Altitude | 0.002 | -0.329 | 0.279 | 0.000 | 0.238 | -0.336 | -0.285 | 0.293 |
| Mean temperature warmest quarter | 0.171 | 0.363 | -0.184 | 0.010 | 0.154 | 0.103 | 0.039 | -0.002 |
| Mean temperature coldest quarter | -0.217 | 0.337 | 0.108 | -0.045 | -0.033 | -0.104 | 0.048 | 0.142 |
| Annual precipitation | -0.311 | -0.063 | -0.073 | 0.200 | 0.203 | 0.139 | 0.049 | 0.054 |
| Precipitation wettest period | -0.288 | -0.011 | 0.065 | 0.372 | 0.213 | 0.193 | 0.028 | 0.013 |
| Precipitation driest period | -0.210 | -0.078 | -0.347 | -0.404 | 0.253 | 0.002 | -0.280 | -0.139 |
| Precipitation seasonality | 0.055 | 0.233 | 0.376 | 0.205 | 0.090 | -0.024 | -0.707 | -0.420 |
| Precipitation wettest quarter | -0.289 | -0.030 | 0.053 | 0.367 | 0.224 | 0.202 | 0.058 | 0.010 |
| Precipitation driest quarter | -0.227 | -0.078 | -0.349 | -0.374 | 0.228 | -0.003 | -0.238 | -0.090 |
| Precipitation warmest quarter | -0.276 | -0.092 | 0.045 | -0.054 | 0.116 | 0.542 | -0.030 | 0.085 |
| Precipitation coldest quarter | -0.182 | -0.018 | -0.294 | 0.334 | 0.228 | -0.580 | 0.240 | -0.393 |
| Annual mean tempretaure | -0.059 | 0.447 | 0.004 | -0.059 | 0.066 | 0.005 | 0.057 | 0.072 |
| Mean diurnal range | 0.219 | 0.023 | 0.243 | -0.101 | 0.565 | -0.122 | 0.125 | 0.288 |
| Isothermality | -0.299 | 0.047 | 0.136 | -0.177 | 0.041 | -0.257 | -0.011 | 0.340 |
| Temperature seasonality | 0.307 | -0.072 | -0.212 | 0.045 | 0.129 | 0.165 | -0.013 | -0.138 |
| Maximum temperature warmest period | 0.213 | 0.312 | -0.128 | 0.040 | 0.293 | 0.037 | 0.059 | 0.102 |
| Minimum temperature coldest period | -0.243 | 0.304 | -0.016 | -0.037 | -0.175 | -0.110 | 0.001 | 0.063 |
| Temperature annual range | 0.316 | -0.044 | -0.065 | 0.053 | 0.312 | 0.107 | 0.035 | 0.014 |
| Mean temperature wettest quarter | -0.094 | 0.337 | 0.259 | -0.285 | 0.155 | 0.039 | 0.159 | -0.264 |
| Mean temperature driest quarter | 0.063 | 0.229 | -0.427 | 0.303 | -0.113 | -0.055 | -0.407 | 0.466 |
|  |  |  |  |  |  |  |  |  |
| Proportion explained by each PC | 43.446 | 23.528 | 10.304 | 6.286 | 5.278 | 3.142 | 2.743 | 2.242 |
| Accumulated variation proportion | 43.446 | 66.974 | 77.278 | 83.564 | 88.843 | 91.984 | 94.728 | 96.969 |
| Principal components eigenvalues | 8.689 | 4.706 | 2.061 | 1.257 | 1.056 | 0.628 | 0.549 | 0.448 |
